# Site‐Selective Installation of N^*ϵ*^‐Modified Sidechains into Peptide and Protein Scaffolds via Visible‐Light‐Mediated Desulfurative C–C Bond Formation

**DOI:** 10.1002/anie.202110223

**Published:** 2021-12-03

**Authors:** Rhys C. Griffiths, Frances R. Smith, Jed E. Long, Daniel Scott, Huw E. L. Williams, Neil J. Oldham, Robert Layfield, Nicholas J. Mitchell

**Affiliations:** ^1^ School of Chemistry University of Nottingham University Park Nottingham NG7 2RD UK; ^2^ Biodiscovery Institute University of Nottingham University Park Nottingham NG7 2RD UK; ^3^ School of Life Sciences, Queen's Medical Centre University of Nottingham Nottingham NG7 2UH UK

**Keywords:** bioconjugation, cysteine, peptides, photochemistry, site selectivity

## Abstract

Post‐translational modifications (PTMs) enhance the repertoire of protein function and mediate or influence the activity of many cellular processes. The preparation of site‐specifically and homogeneously modified proteins, to apply as tools to understand the biological role of PTMs, is a challenging task. Herein, we describe a visible‐light‐mediated desulfurative C(sp^3^)–C(sp^3^) bond forming reaction that enables the site‐selective installation of N^*ϵ*^‐modified sidechains into peptides and proteins of interest. Rapid, operationally simple, and tolerant to ambient atmosphere, we demonstrate the installation of a range of lysine (Lys) PTMs into model peptide systems and showcase the potential of this technology by site‐selectively installing an N^*ϵ*^Ac sidechain into recombinantly expressed ubiquitin (Ub).

## Graphical abstract

We describe a rapid light‐activated C(sp^3^)–C(sp^3^) bond‐forming reaction that enables installation of *N* ^*ϵ*^‐modified sidechains into peptides and proteins at defined positions. Selective for cysteine residues, the method proceeds via interception of free‐radical‐mediated desulfurization. The chemistry is operationally simple, tolerant to ambient atmosphere, and retains the native stereochemistry at the modified residue.

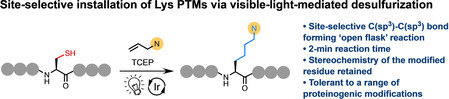

Post‐translational modifications (PTMs) play a crucial role in protein function, controlling a myriad of biological pathways that influence normal cell biology and disease progression.^[1]^ The installation of PTMs, a process largely regulated by enzymes, occurs at many of the canonical amino acids.^[2]^ Such modifications significantly expand the diversity of chemical groups available across the proteome resulting in a considerable variety of protein isoforms that govern precise control over biological systems. The amino acid lysine (Lys) is known to display a particularly broad range of PTMs including methylation,^[3]^ acetylation,^[4]^ propionylation/butyrylation,^[5]^ succinylation,^[6]^ formylation,^[7]^ benzoylation,^[8]^ β‐hydroxybutylation,^[9]^ and several others.^[10]^ These modifications mediate processes such as DNA packaging, gene expression, metabolism, and protein degradation.^[10]^ Despite significant research within this field, the function of the entire library of Lys PTMs remains largely unknown. It is therefore necessary to prepare natively modified proteins to better understand the molecular mechanisms in which they are involved. Thus, techniques that allow the generation of site‐specifically modified proteins are in high demand.

Non‐canonical amino acids can be incorporated into proteins via the technique of amber codon suppression.^[11]^ While this strategy is undeniably powerful, it requires customized tRNA and extensive mutation of the relevant enzymatic machinery. Due to the complexities of this technique, synthetic approaches have also been sought. The total chemical synthesis of modified proteins via peptide ligation using native chemical ligation[^12^, ^13^] (NCL), expressed protein ligation (EPL),[^14^, ^15^] and various advances to these methods,[^16^, ^17^, ^18^, ^19^, ^20^] has significantly contributed to the study of PTMs. However, a more accessible approach involves the preparation of a library of modified proteins from recombinantly expressed material via site‐selective modification of specific residues. Such techniques must be chemoselective in the presence of the diverse chemical functionality displayed by the 20 canonical amino acids. This selectivity must also be effective under mild conditions i.e., in an aqueous environment at neutral pH, under ambient temperature and atmosphere. Owing to the superior nucleophilicity of the thiol group of cysteine (Cys), and its relatively low abundance across the eukaryotic proteome (ca. 2 %), several popular techniques exploit this residue to selectively install PTMs and PTM mimics.

Utilizing appropriate reagents, mimics of several PTMs have been installed at Cys residues via nucleophilic substitution^[21]^ and thiol–ene^[22]^ chemistry (Figure 1 A), including Lys methylation and acetylation. Conversion of Cys residues to dehydroalanine (Dha) has also been explored to install Lys PTM mimics via Michael addition, with loss of native stereochemistry at the modified residue.^[23]^ While such mimics do enable biology mediated by PTMs to be investigated, methods that result in the formation of the native modification are preferred. Recent developments employing free‐radical‐addition to Dha residues enable C(sp^3^)–C(sp^3^) bond forming chemistry to be achieved to install PTMs (Figure 1 B).[^24^, ^25^, ^26^] This technique is versatile and powerful; however, the reaction is not stereoselective, and the chemistry must be carried out under an inert atmosphere, necessitating use of a glove box and degassing of all solutions.

**Figure 1:**
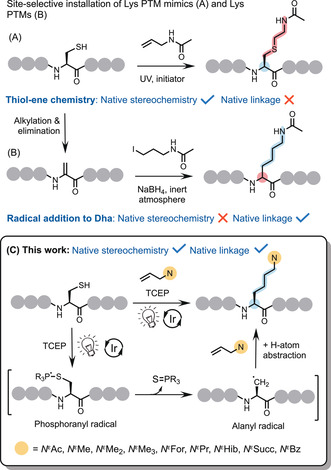
Synthetic approaches to the site‐selective installation of Lys PTM mimics (A) and PTMs (B); *N* ^*ϵ*^Ac shown. Our approach (C): site‐selective installation of *N* ^*ϵ*^‐modified sidechains via interception of visible‐light‐mediated desulfurization.

Previous work within our group describes a site‐selective method for the modification of peptides and proteins via interception of free‐radical‐mediated dechalcogenation.^[27]^ This reaction proceeds via trapping of an alanyl‐radical intermediate produced during phosphine‐mediated desulfurization/deselenization of Cys/selenocysteine (Sec) using a bespoke persistent radical trap with retention of stereochemistry at the modified residue. Further to this study, we postulated that by employing appropriately modified allylamine as the trap, this approach could be exploited to selectively install the *N* ^*ϵ*^‐modified sidechain of Lys via C(sp^3^)–C(sp^3^) bond formation under ambient atmosphere without eliminating the stereochemistry at the modified residue (Figure 1 C). Addition of a primary alkyl radical to an unactivated alkene is a significant synthetic challenge. Free‐radical‐mediated C−C bond formation relies on an initial stabilized radical and/or addition onto an activated alkene.^[28]^ Due to the necessary constraints imposed upon our approach to enable the installation of native chemistry, limiting H‐atom abstraction by the intermediate alanyl radical was identified as our main obstacle. However, within the context of polypeptide bioconjugation at milligram/microgram scale, a large excess of the alkene can practically be employed to push the reaction towards completion in an attempt to out‐compete the undesired pathway.

To explore this concept, peptide **1** (Ac‐CWHISKEY‐NH_2_, a model carrying the majority of proteinogenic chemical functionality) was treated to desulfurization conditions[^29^, ^30^] in the presence of a large excess of *N*‐allyl acetamide. At a concentration of 1 mM (**1** dissolved into 20 % dimethylsulfoxide (DMSO) in 6 M guanidinium hydrochloride (Gdn⋅HCl), 0.1 M Na_2_HPO_4_, pH 7), the peptide was irradiated under blue light in the presence of an iridium(III) photocatalyst ((Ir[dF(CF_3_)ppy]_2_(dtbpy))PF_6_, 1 mol %) to generate a thiyl radical. The water‐soluble phosphine, TCEP (tris(2‐carboxyethyl)‐phosphine; 50 mM), was employed to facilitate desulfurization via a phosphoranyl radical, and *N*‐allyl acetamide (**2**) (500 mM) was introduced to trap the intermediate alanyl radical. Irradiation was achieved using a simple apparatus consisting of inexpensive blue LEDs wrapped around a Pyrex dish on a stirrer plate (photochemistry set up 1, Supporting Information). Encouragingly, we observed full consumption of the starting peptide in under 2 hours to afford product **3 a** with the mass of the desired conjugate at 36 % conversion (Table 1, entry 1). The alanine (Ala)‐containing by‐product, **3 b** (Ac‐AWHISKEY‐NH_2_), produced through trapping of the alanyl radical via H‐atom abstraction, accounted for the majority of the remaining material (with a small percentage of a multiple addition product detected).

**Table 1:** Optimization of visible‐light‐mediated installation of *N* ^*ϵ*^Ac sidechains; iridium(III) catalyst, (Ir[dF(CF_3_)ppy]_2_(dtbpy))PF_6_, shown. 
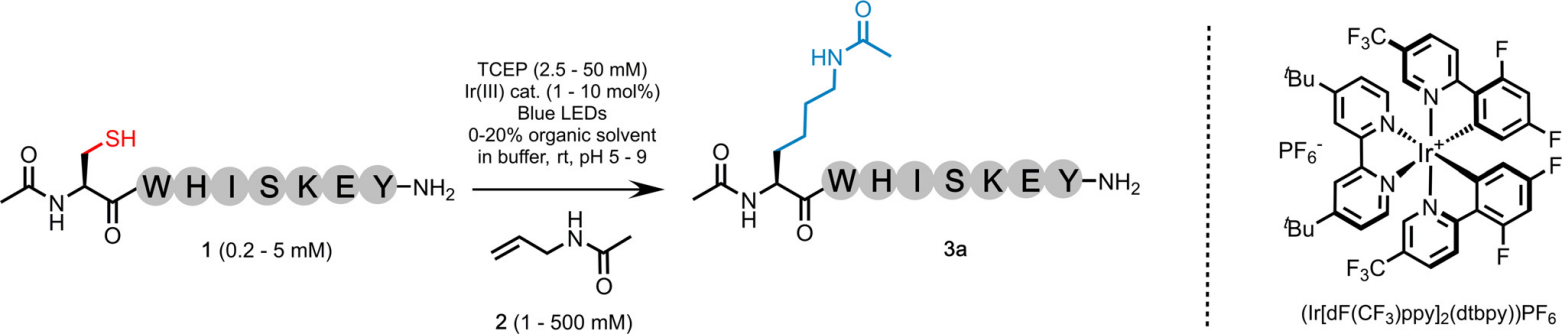

Entry	Peptide [mM]^[a]^	*N*‐allyl acetamide **2** [mM]	Catalyst [mol %]	TCEP [mM]	pH	Conversion [% **1** to **3 a**]^[b]^	Time to completion^[d]^
1	1	500	1	50	7	36	<2 hours
2	0.5	250	1	50	7	43	<2 hours
3	0.5	250	1	2.5	7	42	<2 hours
4	0.5	250	1	2.5	8	52	<2 hours
5	0.5	250	5	2.5	8	51	<1 hour
**6**	**0.5**	**100**	**5**	**2.5**	**8**	**55 [51]^[c]^**	**45 mins**
**7**	**0.5**	**100**	**5**	**2.5**	**8**	**47 [54]^[c]^**	**2 mins^[e]^**
8	0.5	100	10	2.5	8	49	<1 hour

[a] 6 M Gdn⋅HCl, 0.1 M Na_2_HPO_4_, organic component DMSO, MeOH or MeCN. [b] % conversion calculated by analytical HPLC. [c] Isolated yield given in brackets. [d] All reactions conducted under blue LED lights at room temp. (set up 1), except for entry 7. [e] PhotoRedOx Box used for entry 7 (set up 2); 34 mW cm^−2^ LED bulb, 450 nm.

To optimize the reaction, the relevant variables were systematically evaluated (Table 1 and Supporting Information). The reaction proceeds only in the presence of the photocatalyst under irradiation of blue light (450–495 nm). Decreasing the peptide concentration to 0.5 mM improved the conversion (entry 2). Decreasing the TCEP concentration to 2.5 mM was not observed to hinder the reaction (entry 3). Employing an alternative photocatalyst (eosin Y) did not improve the conversion (Supporting Information). The optimal pH was found to be 8 (entry 4); increasing the iridium catalyst to 5 mol % decreased the time to completion to 60 mins with no change in yield (entry 5). Reducing the concentration of *N*‐allyl acetamide to 100 mM did not hinder to the reaction (entry 6); below this threshold the reaction conversion decreased (Supporting Information). The % conversion to product was comparable when the reaction was run in buffer with no organic component (Supporting Information). The optimal conditions for this model appeared to be entry 6 (referred to hereafter as protocol A), affording 55 % conversion by analytical HPLC. A preparative‐scale reaction was run using this protocol and the progress monitored closely by HPLC. The starting peptide was consumed over 45 mins and the desired product **3 a** was isolated in 51 % yield. Due to the use of an unactivated alkene, we were unable to fully out‐complete H‐atom abstraction. Under the optimized conditions, the Ala by‐product accounts for less than 40 % of the remaining material, while multiple addition of *N*‐allyl acetamide accounts for less than 10 %. The reaction did not require degassing and could be run “on the bench” under ambient atmosphere. Performing the reaction using degassed solvents in a glove box did not improve conversion. Protocol A was then repeated in a temperature controlled PhotoRedOx Box (HepatoChem) using a 34 mW cm^−2^ LED bulb at 450 nm (set up 2, Supporting Information). Remarkably, the reaction reached completion within just 2 mins using this apparatus, in comparable conversion and isolated yield (entry 7). Finally, increasing the mol % of the catalyst above 5 mol % did not improve the conversion for this model (entry 8).

To ensure the stereochemistry at the modified residue was not compromised during the reaction, model peptides Ac‐CAY‐NH_2_ (**4 a**) and Ac‐d‐CAY‐NH_2_ (**4 b**) were synthesized and subjected to protocol A, yielding the desired epimers **5 a** and **5 b**. Comparison of the NMR spectra of these products with the NMR of **5 a** synthesized on the solid phase confirmed that the integrity of the α‐stereocentre of the target residue remains intact throughout the modification process (Figure 2). Furthermore, to explore the installation of multiple modifications, peptide **6 a** (Ac‐YECPLAHISCKY‐NH_2_) was prepared and subjected to protocol A using trap **2**. As expected, several products were identified as the various permutations of the *N* ^*ϵ*^Ac sidechain and Ala at the two Cys sites (Supporting Information) indicating that this method is only suitable for the installation of single‐point modifications.

**Figure 2:**
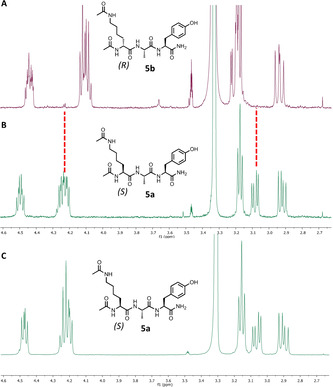
NMR spectra (CHα region) of *N* ^*ϵ*^Ac sidechain installation using model peptides **4 a** and **4 b** to afford epimers **5 a** (B) and **5 b** (A); NMR spectra of **5 a** synthesised on the solid phase (C).

To further probe the effect of the local chemical environment on the reaction, a small selection of tetrapeptides was prepared and subjected to protocol A using trap **2** (Ac‐XCAY‐NH_2_; X=Lys (**7**), Glu (glutamic acid; **8**), Tyr (tyrosine; **9**), Ile (isoleucine; **10**)). These models carry an H‐atom donor (phenol), charged groups at neutral pH (ammonium and carboxylate groups), and steric encumbrance directly adjacent to the target Cys. The extent of Ala by‐product formation and multiple addition varied slightly across this series, however the generation of the desired products (**11**–**14**) remained broadly consistent with the previous model (see Table 2). Co‐elution of product **11** with impurities impeded isolation of the desired product for this example.

**Table 2:** Effect of H‐atom donor, electrostatics, and steric encumbrance on the efficiency of the reaction. 
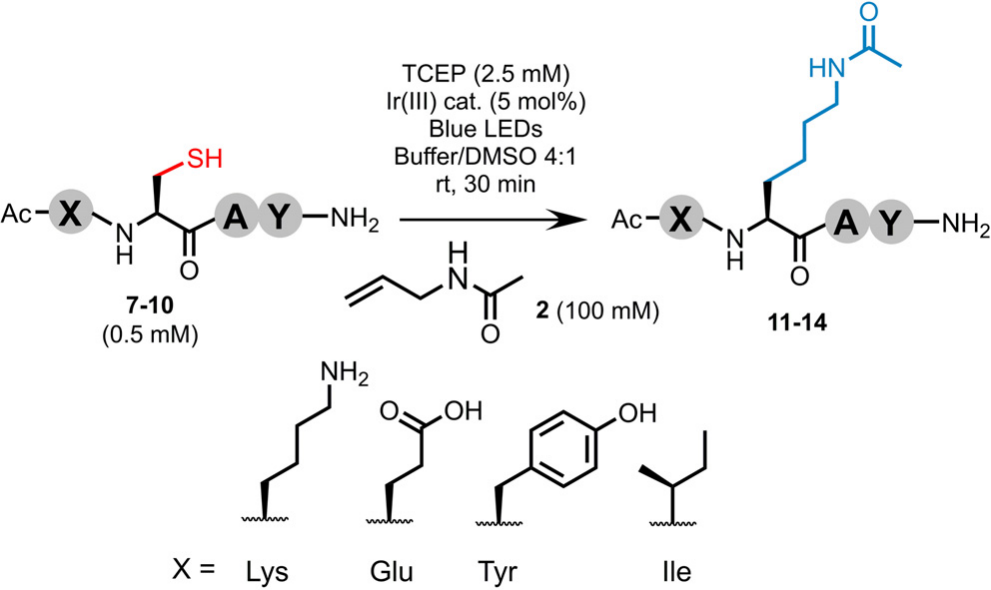

Entry	X=	Desired product [%]^[a]^	Isolated yield [%]
9	Lys (**7**)	34 (**11**)	23 (**11**)^[c]^
10	Glu (**8**)	40 (**12**)	31 (**12**)
11	Tyr (**9**)	46 (**13**)	59 (**13**)
12	Ile (**10**)	53 (**14**)^[b]^	54 (**14**)

[a] % conversion calculated by analytical HPLC. [b] Similar retention times for product **14** and by‐products limited accurate determination of the ratios. [c] Co‐eluting impurities impeded isolation of product **11**.

To investigate the site‐selective installation of a range of *N* ^*ϵ*^‐modified sidechains, several modified allyl compounds were synthesized starting from allyl amine or allyl chloride to afford a small library of traps (**2**, **15**–**22**) (Figure 3; see Supporting Information for experimental details). Using our method, these traps enable installation of a range of Lys PTMs into polypeptides including acetylation (**2**), methylation (**15**–**17**), formylation (**18**), propionylation (**19**), β‐hydroxybutylation (**20**), succinylation (**21**), and benzoylation (**22**). A 10 mer sequence representing residues 32–41 of histone H2A (*homo sapiens*) (**23**) was synthesized as a biologically relevant model to explore the reaction scope using these traps. Protocol A conditions were applied using trap **2** to afford the desired product **24** in 50 % conversion (by analytical HPLC) over 3 hours using set up 1. Slight alterations to this protocol (i.e., an increase in the excess of TCEP from 2.5 mM to 5 mM and the catalyst from 5 mol % to 10 mol %) afforded a higher conversion for this model over 1 hour (62 %—referred to as protocol B). The protocol B conditions were then applied on a preparative scale to model peptide **23** with traps **2**, **15**–**22** in our simple photochemistry set up 1 to afford a selection of peptides bearing an internally modified Lys residue (**24**–**32**) in moderate‐good yield (47–62 %), with full consumption of **23** in under 60 mins (Figure 4). These substrate‐scope reactions were then repeated in the PhotoRedOx Box (set up 2) using protocol B, for comparison. As observed previously, the reactions reached completion within 2 mins to afford the desired products **24**–**32** in comparable isolated yield to those run using set up 1 (48–68 %; Figure 4). Furthermore, to explore the installation of a Lys PTM into a cyclic peptide, model **33** (c[WHISCKEY]) was synthesized and subjected to protocol B (set up 2) using probe **16** to afford peptide **34** in 46 % isolated yield (see Supporting Information).

**Figure 3:**
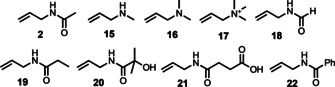
Modified allylamine traps **2**, **15**–**22**.

**Figure 4:**
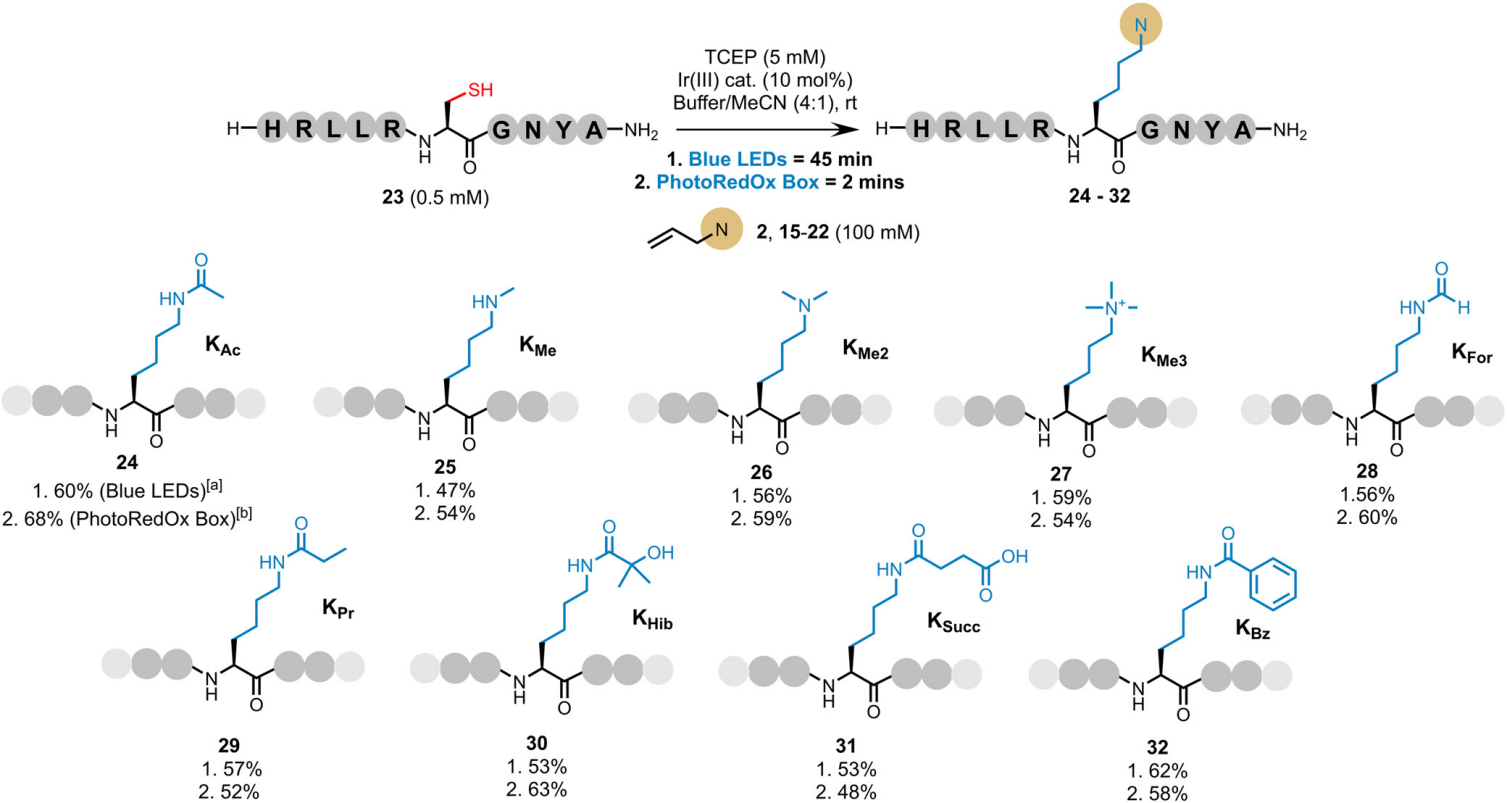
Site‐selective installation of a range of Lys PTMs via visible‐light‐mediated, desulfurative C(sp^3^)–C(sp^3^) bond formation. [a] Photochemical set‐up 1 (blue LEDs). [b] Photochemical set‐up 2 (PhotoRedOx Box); isolated yields shown.

Satisfied that this method enabled effective installation of a range of PTMs in model peptide systems, we applied our reaction to investigate the incorporation of a biologically relevant PTM using recombinantly expressed protein material (Figure 5). Acetylation of ubiquitin (Ub) at Lys48 (K48), regulates polyubiquitination of protein substrates and thus controls proteasome‐mediated protein degradation.^[31]^ Employing a K48C mutant of Ub (**35**) (the native Ub sequence contains no Cys residues, see Supporting Information), produced via recombinant expression in *E. coli*,^[32]^ we subjected the material to protocol B in the presence of *N*‐allyl acetamide (**2**) in set up 1 to demonstrate the accessibility of our technique using inexpensive apparatus. Sub‐optimal conversion to the product prompted us to increase the concentration of TCEP described in protocol B to 50 mM (referred to as protocol C).

**Figure 5:**
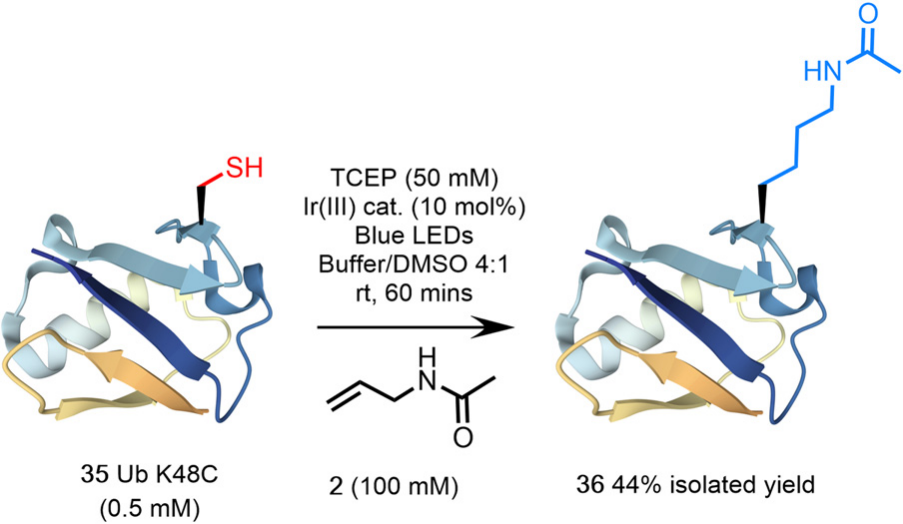
Site‐selective installation of an *N* ^*ϵ*^Ac sidechain into recombinantly expressed Ub **35** (K48C mutant).

Gratifyingly, the reaction proceeded to the expected conversion over 60 mins using these conditions. The desired product **36** was isolated by preparative HPLC in 44 % yield (Figure 5). Considering the size and chemical complexity of Ub, the operational simplicity of the protocol, and the application of this method to the installation of native modifications via C(sp^3^)–C(sp^3^) bond formation, we consider the yield of **36** to be acceptable. To ensure the modified Ub could be re‐folded from the denaturing conditions of the bioconjugation, product **36** was re‐dissolved in 6 M Gdn⋅HCl, 0.1 M Na_2_HPO_4_, pH 7, folded via dialysis into 25 mM Na_2_HPO_4_, 100 mM NaCl, pH 7.0, and analyzed via ^1^H NMR. The extended NH region of the spectra (6.5–9.5 ppm), and upshifted methyl signals indicating hydrophobic packing, were consistent with folded WT Ub.^[33]^ The modified *N* ^*ϵ*^Ac‐Ub **36** protein retained integrity, based on migration on an SDS‐PAGE gel, and comparable recognition to K48C‐Ub was observed via western blot using an anti‐ubiquitin antibody (Supporting Information).

In conclusion, we have developed a rapid and operationally simple reaction that enables the site‐selective installation of Lys PTMs into complex polypeptides via C(sp^3^)–C(sp^3^) bond formation. The reaction proceeds via visible‐light‐mediated desulfurization under ambient conditions in the presence of an unactivated alkene and is tolerant to a range of modifications. The optimized protocol translates well to a small protein system, enabling isolation of modified material in acceptable yield. To our knowledge, this report represents the first example of direct, late‐stage installation of Lys PTMs via chemical conversion of a canonical residue without disrupting the stereochemistry of the target amino acid. Due to the operational simplicity of the reaction, and inexpensive experimental set‐up, the described protocols are accessible to researchers without extensive training in synthetic chemistry to enable the production of biochemical tools to further expand our knowledge of the chemically modified proteome.

## Conflict of interest

The authors declare no conflict of interest.

## Supporting information

As a service to our authors and readers, this journal provides supporting information supplied by the authors. Such materials are peer reviewed and may be re‐organized for online delivery, but are not copy‐edited or typeset. Technical support issues arising from supporting information (other than missing files) should be addressed to the authors.

Supporting Information
