# Supplementary material for: Site‐Selective Installation of N ϵ ‐Modified Sidechains into Peptide and Protein Scaffolds via Visible‐Light‐Mediated Desulfurative C–C Bond Formation
Source: Angew Chem Int Ed Engl. 2021 Dec 3;61(2):e202110223. doi: 10.1002/anie.202110223 (PMC9299887; doi:10.1002/anie.202110223)
Supplement: Supplementary file 1 — Supporting Information [file ANIE-61-0-s001.pdf]

Angewandte Chemie
Eine Zeitschrift der Gesellschaft Deutscher Chemiker

Supporting Information

Site-Selective Installation of N$^{\varepsilon}$-Modified Sidechains into Peptide and Protein Scaffolds via Visible-Light-Mediated Desulfurative C–C Bond Formation

*Rhys C. Griffiths+, Frances R. Smith+, Jed E. Long, Daniel Scott, Huw E. L. Williams, Neil J. Oldham, Robert Layfield, and Nicholas J. Mitchell**

anie_202110223_sm_miscellaneous_information.pdf

Contents

General Methods 2

Materials 3

Solid-phase peptide synthesis (SPPS) 3

Manual Fmoc-SPPS 3

Automated Fmoc-SPPS 4

General conjugation protocol A 4

General conjugation protocol B 5

General conjugation protocol C 5

Photochemistry apparatus 6

Compound synthesis 7

Model peptides 11

Initial optimization of visible-light-mediated desulfurative C(sp^3)-C(sp^3) bond formation 22

Investigation of retention of stereochemistry at the modified residue 31

Modification of multiple Cys residues 36

Exploration of local chemical environment 37

Installation of native N^{ε}-modifications into a histone model peptide 45

Substrate scope on histone model peptide 47

Site-selective modification of a cyclic peptide 71

Site-selective installation of N^{ε}Ac Lys into K48C ubiquitin 72

^{1}H NMR analysis of folded N^{ε}Ac-Ub 76

Appendix 78

General Methods

NMR samples were analysed on Bruker AVIII 400 MHz (^{1}H-NMR frequency 400 MHz; ^{13}C-NMR frequency 100 MHz) or Bruker AVIII 500 MHz (^{1}H-NMR frequency 500 MHz; ^{13}C-NMR frequency 125 MHz) NMR systems. Folded ubiquitin samples were analysed on a Bruker 600 MHz Avance III (^{1}H-NMR frequency 600 MHz). Chemical shifts are reported in parts per million (ppm) and are referenced to solvent residual signals: $CDCl_3$ (δ 7.26 [^{1}H]), DMSO (δ 2.50 [^{1}H]), MeOD (δ 3.31 [^{1}H]). ^{1}H-NMR data is reported as chemical shift (δ), multiplicity (s = singlet, d = doublet, t = triplet, q = quartet, dd = doublet of doublets, ddd = doublet of doublet of doublets or combinations of these multiples; m = unassigned multiplet), relative integral, coupling constant (J Hz) and assignment where possible.

High-resolution mass spectra were recorded on a Bruker MicroTOF Focus II MS (ESI) operating in positive or negative ionisation mode. Analytical HPLC was performed on a Thermo Ultimate 3000 uHPLC system equipped with PDA eλ detector (λ = 210 – 400 nm). Peptides were analyzed using a Waters Sunfire 5 µm, 2.1 x 150 mm column (C-18) at a flow rate of 0.6 mL min^{-1}. The mobile phase composed of 0.1% trifluoroacetic acid in H_2O (Solvent A) and 0.1% trifluoroacetic acid in acetonitrile (Solvent B). The analysis of the chromatograms was conducted using Chromeleon 7 software.

ESI-MS/MS analysis was performed on a ThermoFisher LTQFTUltra mass spectrometer using the standard electrospray source operated at 4.5 kV. A 1 mg mL^{-1} sample of *N*Ac-Ub (**36**) was diluted 100-fold in a mixture of 1:1 water:acetonitrile containing 0.1% formic acid and infused directly into the mass spectrometer using a syringe pump. Collision induced dissociation (CID) was performed on the $[M+11H]^{11+}$ ion of **36** (*m/z* 782) in the ion trap of the instrument, using helium buffer gas and an activation energy of 20 (rel. units). Product ions were transferred to the FTICR cell and the spectrum was recorded using a resolving power of 100,000 (*m/z* 400). The MS/MS spectrum was viewed in Xcalibur (ThermoFisher Ltd.) and deconvoluted using the Xtract tool.

Preparative reverse-phase HPLC was performed using a Waters 1525 binary pump HPLC equipped with a dual wavelength UV detector set to 210 nm and 280 nm. Peptides were purified on a Waters Sunfire 5 µm (C-18) preparative column with 5-µm particle size, 19 x 150 mm, operating at a flow rate of 6 mL min^{-1} using a mobile phase of 0.1% trifluoroacetic acid in water (Solvent A) and 0.1% trifluoroacetic acid in acetonitrile (Solvent B) using the gradient specified in the experimental section. Semi-preparative reverse-phase HPLC was performed using the same HPLC and solvent system. The column used was a Waters Sunfire 5 µm (C-18) preparative column, 10 x 250 mm, operating at a flow rate of 5 mL min^{-1} using the gradient specified in the experimental section.

Materials

Commercial materials were used as received unless otherwise noted. Amino acids, coupling reagents and resins were obtained from Novabiochem, Fluorochem or GL Biochem. Compounds **15** and **16** were commercially available and used as received. Reagents that were not commercially available were synthesized as outlined in the experimental section. Solvents were obtained as reagent grade from Merck or Fisher.

Solid-phase peptide synthesis (SPPS)

Manual Fmoc-SPPS

Preloading Rink Amide resin: Rink amide resin was initially washed with DCM (5 × 3 mL) followed by removal of the Fmoc group by treatment with 20% piperidine/DMF (2 × 5 min). The resin was washed with DMF (5 × 3 mL), DCM (5 × 3 mL) and DMF (5 × 3 mL). Oxyma pure (4 eq.) and DIC (4 eq.) were added to a solution of Fmoc-AA-OH (4 eq.) in DMF. After 5 min of pre-activation, the mixture was added to the resin. After 2 h the resin was washed with DMF (5 × 3 mL), DCM (5 × 3 mL) and DMF (5 × 3 mL), capped with acetic anhydride/pyridine (1:9 v/v) (2 × 3 min) and washed with DMF (5 × 3 mL), DCM (5 × 3 mL) and DMF (5 × 3 mL).

General amino acid coupling: A solution of protected amino acid (4 eq.), DIC (4 eq.) and Oxyma pure (4 eq.) in DMF (final concentration 0.1 M) was added

to the resin. After 1 h, the resin was washed with DMF (5 × 3 mL), DCM (5 × 3 mL) and DMF (5 × 3 mL).

Deprotection: The resin was treated with 20% piperidine/DMF (2 x 3 mL, 3 min) and washed with DMF (5 × 3 mL), DCM (5 × 3 mL) and DMF (5 × 3 mL).

Capping: Acetic anhydride/pyridine (1:9 v/v) was added to the resin (3 mL). After 3 min the resin was washed with DMF (5 × 3 mL), DCM (5 × 3 mL) and DMF (5 × 3 mL).

Cleavage: A mixture of TFA, thioanisole, tri*iso*propylsilane (TIS) and water (90:4:4:2 v/v/v/v) was added to the resin. After 3 h, the resin was washed with TFA (3 × 2 mL).

Work-up: The combined solutions were concentrated under a stream of nitrogen to < 5 mL. 40 mL of diethyl ether was added to precipitate the peptide and the suspension centrifuged. The pellet was then dissolved in water containing 0.1% TFA, filtered and purified by preparative HPLC and analyzed by LC-MS and ESI mass spectrometry.

Automated Fmoc-SPPS

Automated Fmoc-SPPS was carried out on a Biotage Initiator^{+} Alstra microwave peptide synthesizer. General synthetic procedures for Fmoc-deprotection and capping were carried out in accordance with the manufacturer's specifications. Standardized amino acid couplings were performed for 15 min at 50 ^{o}C under microwave irradiation in the presence of amino acid (0.5 M in DMF, 4 eq.), Oxyma (0.5 M in DMF, 4 eq.) and di*iso*propylcarbodiimide (0.5 M in DMF, 4 eq.). Peptide cleavage and work-up were carried out as described above for manual SPPS.

General conjugation protocol A

To peptide dissolved in 20% acetonitrile (ACN) in 6 M Gdn•HCl, 0.1 M Na_2HPO_4, pH 7 to a concentration of 1 mM was added a solution of TCEP (0.5 M stock solution in LB pH adjusted to 7, 5 eq.), allyl compound (200 eq.) and $(Ir[dF(CF_3)ppy]_2(dtbpy))PF_6$ (1 mM stock solution in ACN, 0.05 eq.). The pH of

the reaction mixture was checked to be 8 then the reaction mixture diluted to the final peptide concentration of 0.5 mM. The reaction vessel was then placed into blue LEDs (photochemistry set up 1), or into a PhotoRedOx Box (HepatoChem) (photochemistry set up 2); once the starting material was shown to be fully consumed by analytical HPLC, the reaction mixture was purified by semi-preparative HPLC.

General conjugation protocol B

To peptide dissolved in 20% acetonitrile (ACN) in 6 M Gdn•HCl, 0.1 M Na_2HPO_4, pH 7 to a concentration of 1 mM was added a solution of TCEP (0.5 M stock solution in LB pH adjusted to 7, 10 eq.), allyl compound (200 eq.) and $(Ir[dF(CF_3)ppy]_2(dtbpy))PF_6$ (1 mM stock solution in ACN, 0.1 eq.). The pH of the reaction mixture was checked to be 8 then the reaction mixture diluted to the final peptide concentration of 0.5 mM. The reaction vessel was then placed into blue LEDs (photochemistry set up 1), or into a PhotoRedOx Box (HepatoChem) (photochemistry set up 2); once the starting material was shown to be fully consumed by analytical HPLC, the reaction mixture was purified by semi-preparative HPLC

General conjugation protocol C

To peptide dissolved in 20% acetonitrile (ACN) in 6 M Gdn•HCl, 0.1 M Na_2HPO_4, pH 7 to a concentration of 1 mM was added a solution of TCEP (0.5 M stock solution in LB pH adjusted to 7, 100 eq.), allyl compound (200 eq.) and $(Ir[dF(CF_3)ppy]_2(dtbpy))PF_6$ (1 mM stock solution in ACN, 0.1 eq.). The pH of the reaction mixture was checked to be 8 then the reaction mixture diluted to the final peptide concentration of 0.5 mM. The reaction vessel was then placed into blue LEDs (photochemistry set up 1); once the starting material was shown to be fully consumed by analytical HPLC, the reaction mixture was purified by semi-preparative HPLC

Photochemistry apparatus

Set up 1

A blue LED light strip wrapped around a pyrex dish, placed on top of a stirrer plate. To ensure consistency, places for up to 4 vials were marked on the plate. The temperature was monitored and observed to reach no higher than 30 °C.

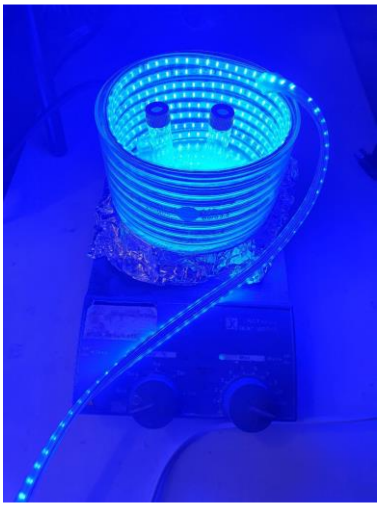

Set up 2

To compare set up 1 with specialized equipment, all reactions were also carried out in a temperature controlled PhotoRedOx Box (HCK1006-01-016, HepatoChem) operated with a 450 nm, 34 mW/cm^2 bulb (450PF, HCK1012-01-002, Hapatochem) at room temp.

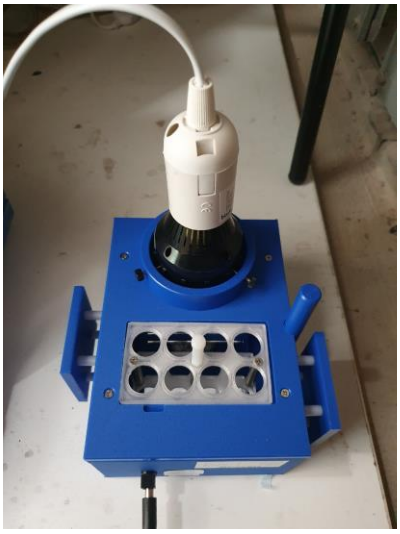

Compound synthesis

N-allylacetamide (2)

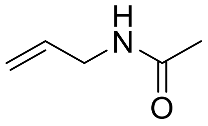

To a solution of allyl amine (10.0 ml, 133.2 mmol) and triethylamine (11.1 ml, 80.0 mmol) in THF (100 ml) at 0 ^{o}C was added acetyl chloride (9.48 ml, 133.2 mmol) dropwise. The reaction was left to stir at room temperature for 2 hours before being purified by vacuum distillation (15 mbar). The desired product eluted at 90 ^{o}C as a colourless oil (12.59 g, 127.2 mmol, 95% yield). HRMS Calc: 100.0762 $[M+H]^+$; Obs: 100.0764 $[M+H]^+$. IR (ATR, cm^{-1}) 3277brm, 3081m, 2986m, 1634s, 1544s, 1422m, 1372m, 1281m, 1037m, 991m, 918m. ^{1}H NMR (400 MHz, $CDCl_3$) δ 6.11 (s, 1H, N*H*), 5.82 (ddt, *J* = 17.2, 10.2, 5.7 Hz, 1H, allyl C*H*), 5.30 – 5.02 (m, 2H, allyl CH_2), 3.86 – 3.83 (m, 2H, CH_2), 1.99 (s, 3H, CH_3). ^{13}C NMR (100 MHz, $CDCl_3$) δ 170.2 (C), 134.2 (CH_2), 116.3 (CH), 42.0 (CH_3), 23.1 (CH_2).

Allyltrimethylammonium chloride (17)

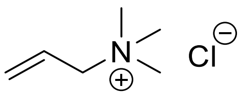

To trimethylamine (12.0 ml, 33% wt solution in ethanol, 92.7 mmol) was slowly added allyl chloride (1.50 ml, 18.5 mmol). The reaction was left to stir at room temperature for 2 hours then concentrated *in vacuo* to give a colourless solid. The solid was then redissolved in chloroform and crystallised by slow addition of acetone. The crystals were then filtered and washed with acetone before drying under a high vacuum to give the desired product as colourless crystals (1.12 g, 8.33 mmol, 45%). HRMS Calc: 100.1121 $[M+H]^+$; Obs: 100.1128 $[M+H]^+$. IR (ATR, cm^{-1}) 3464brw, 3008m, 2943m, 1640w, 1473s, 1404m, 919s. ^{1}H NMR (400 MHz, D_2O) δ 6.14 – 6.02 (m, 1H), 5.79 – 5.69 (m, 2H), 3.96 (d, *J* = 7.4 Hz, 2H), 3.13 (s, 9H). ^{13}C NMR (100 MHz, D_2O) δ 129.2 (CH_2), 124.5 (CH), 68.4 (CH_2), 52.5 (CH_3), 52.4 (CH_3), 52.3 (CH_3).

N-allylformamide (18)

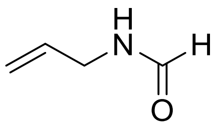

To allylamine (9.86 ml, 131.4 mmol) at 0 °C was added ethyl formate (21.2 ml, 262.8 mmol) dropwise. The reaction mixture was allowed to warm to room temperature and stirred overnight. The solution was concentrated *in vacuo* and purified by vacuum distillation (15 mbar). The product eluted at 85-90 °C as a colourless oil (9.17 g, 107.9 mmol, 82% yield). HRMS Calc: 86.0528 $[M+H]^+$; Obs: 86.0586 $[M+H]^+$. IR (ATR, cm^{-1}) 3285brm, 3051w, 2986w, 2865w, 1656s, 1650s, 1526s, 1382s, 1231m, 991m, 918m. ^1H NMR (400 MHz, $CDCl_3$) δ 8.17 (s, 1H), 6.46 (s, 1H), 5.81 (ddt, J = 17.1, 10.3, 5.6 Hz, 2H), 5.40 – 4.89 (m, 4H), 3.93 – 3.84 (m, 2H). ^{13}C NMR (100 MHz, $CDCl_3$) δ 165.0 +161.4 (C), 134.4 + 133.6 (CH_2), 116.8 + 116.5 (CH), 44.2 + 40.4 (CH_2). NMR showing a mixture of rotamers in a 1:0.2 ratio.

N-Allylpropionamide (19)

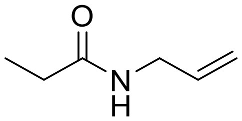

To propionic acid (1.00 ml, 13.4 mmol) in anhydrous DCM (5 ml) 0 °C was added N-allylamine (1.11 ml, 14.8 mmol) and EDC (2.82 g, 14.8 mmol). The reaction mixture was allowed to warm to room temperature and stirred for 16 hours. The reaction mixture was concentrated *in vacuo* to yield a pale-yellow oil. This was purified by column chromatography (pentane:EtOAc 3:1) to yield the title compound as a pale yellow oil (1.11 g, 9.81 mmol, 73%). HRMS Calc: 114.0913 $[M+H]^+$; Obs: 114.0933 $[M+H]^+$. IR (ATR, cm^{-1}) 3284 brm, 3079w, 2978w, 1639s, 1541s. ^1H NMR (400 MHz, $CDCl_3$) δ 5.98 (s, 1H), 5.78 (ddt, J = 17.4, 10.7, 5.6 Hz, 1H), 5.31 – 4.81 (m, 2H), 3.83 (td, J = 4.9, 4.1, 1.7 Hz, 2H), 2.20 (q, J = 7.6 Hz, 2H), 1.12 (t, J = 7.6 Hz, 3H). ^{13}C NMR (100 MHz, $CDCl_3$) δ 173.9 (C), 134.5 (CH_2), 116.2 (CH), 41.9 (CH_2), 29.7 (CH_2), 10.0 (CH_3).

N-Allyl-2-hydroxy-2-methylpropanamide (20)

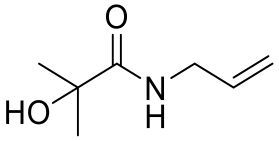

α-Hydroxybutyric acid (300 mg, 2.88 mmol) and N-allylamine (0.24 ml, 3.17 mmol) were dissolved in anhydrous DMF (3 ml). NMM (0.95 ml, 8.65 mmol) and DMTMM (1.19 g, 4.33 mmol) were added. The reaction mixture was stirred at room temperature for 16 hours before being diluted with H_2O (30 ml) and extracted with EtOAc (3 x 30 ml). The combined organic layers were washed with 1 M HCl (3 x 50 ml), saturated $NaHCO_3$ (3 x 50 ml) and brine (50 ml), then dried with $MgSO_4$, filtered and concentrated *in vacuo* to yield a pale orange oil. This was purified by column chromatography (DCM:MeOH 95:5) to yield the title compound as a pale yellow oil (129 mg, 0.90 mmol, 31%). HRMS Calc: 144.1019 $[M+H]^+$; Obs: 144.1021 $[M+H]^+$. IR (ATR, cm^{-1}) 3341brw, 2975w, 2930w, 1639s, 1522s. ^1H NMR (400 MHz, $CDCl_3$) δ 6.98 (s, 1H), 5.82 (ddt, J = 17.3, 10.6, 5.6 Hz, 1H), 5.25 – 5.03 (m, 2H), 3.86 (td, J = 4.9, 4.1, 2.8 Hz, 2H), 1.44 (s, 6H). ^{13}C NMR (100 MHz, $CDCl_3$) δ 176.6 (C), 134.2 (CH_2), 116.4 (CH), 73.7 (C), 41.7 (CH_2), 28.0 (CH_3).

N-Allylsuccinimide (21)

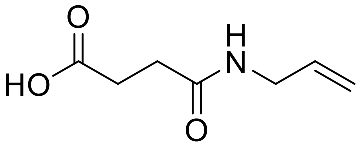

To succinic anhydride (250 mg, 2.50 mmol) in acetone (2 ml) was added N-allylamine (0.23 ml, 3.13 mmol) dropwise. The reaction mixture was stirred at room temperature for 4 hours then concentrated *in vacuo* to give a pale-yellow solid. This was purified by column chromatography (DCM:MeOH:AcOH 90:9:1) to yield the title compound as a pale yellow solid (203 mg, 1.29 mmol, 51%). HRMS Calc: 156.0666 $[M+H]^-$; Obs: 156.0664 $[M+H]^-$. IR (ATR, cm^{-1}) 3297m, 3006brw, 1688s, 1637s, 1539s, 1416m. ^1H NMR (400 MHz, DMSO) δ 12.0 (s, 1H), 7.99 (t, J= 5.8 Hz, 1H), 5.77 (ddt, J = 17.3, 10.3, 5.1 Hz, 1H), 5.34 – 4.77 (m, 2H), 4.00 – 3.37 (m, 2H), 2.47 – 2.28 (m, 4H). ^{13}C NMR (100 MHz, DMSO)

δ 173.9 (C), 170.8 (C), 135.4 (CH_2), 114.9 (CH), 40.8 (CH_2), 29.9 (CH_2), 29.1 (CH_2).

_N_-allylbenzamide (22)

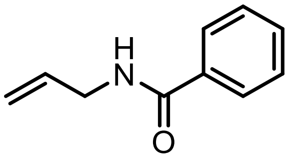

To N-allylamine (0.64 ml, 8.54 mmol) and triethylamine (2.97 ml, 21.3 mmol) in DCM (25 ml) at 0 ^{o}C was added benzoyl chloride (0.83 ml, 7.11 mmol) dropwise. The reaction mixture was allowed to warm to room temperature and stir for 16 hours before being diluted with DCM (25 ml) and washed with 1 M HCl (3 x 10 ml), sat. $NaHCO_3$ (3 x 10 ml) and brine (10 ml). The organic layer was then dried over $MgSO_4$, filtered and concentrated to product a pale-yellow oil. This was purified by column chromatography (DCM) to yield the title compound as a colourless oil, which formed colourless crystals when stored at 4 ^{o}C (0.96 g, 5.96 mmol, 84% yield). HRMS Calc: 162.0919 $[M+H]^+$; Obs: 162.0920 $[M+H]^+$. IR (ATR, cm^{-1}) 3305 brm, 3065w, 2985w, 2916w, 1636s, 1532s, 1420w, 1294s, 1028w, 992s. ^{1}H NMR (400 MHz, DMSO) δ 8.67 (s, 1H), 7.88 (d, J = 7.0 Hz, 2H), 7.59 – 7.39 (m, 3H), 5.91 (ddt, J = 17.3, 10.3, 5.2 Hz, 1H), 5.23 – 5.03 (m, 2H), 4.00 – 3.83 (m, 2H). ^{13}C NMR (100 MHz, DMSO) δ 166.8 (C), 135.9 (Ar), 134.9 (Ar-H), 131.6 (Ar-H), 128.7 (Ar-H), 127.7 (CH_2), 115.6 (CH), 42.0 (CH_2).

Model peptides

Ac-CWHISKEY-NH2 (1)

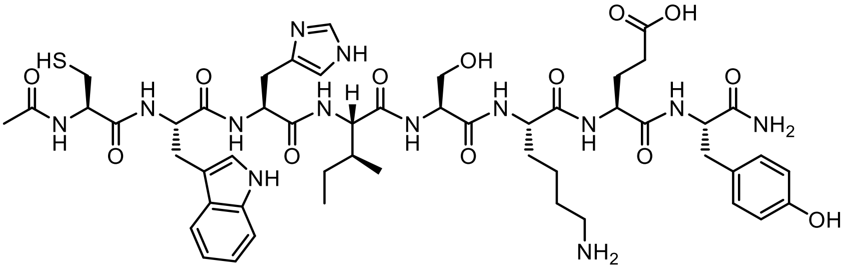

Peptide **1** was synthesized using general automated synthesizer protocol with microwave assistance on rink amide resin (0.2 mmol). The crude peptide was purified by preparative RP-HPLC (15-80% B over 30 minutes) and lyophilized to produce the desired peptide (**1**, 89 mg, 0.08 mmol, 40% yield).

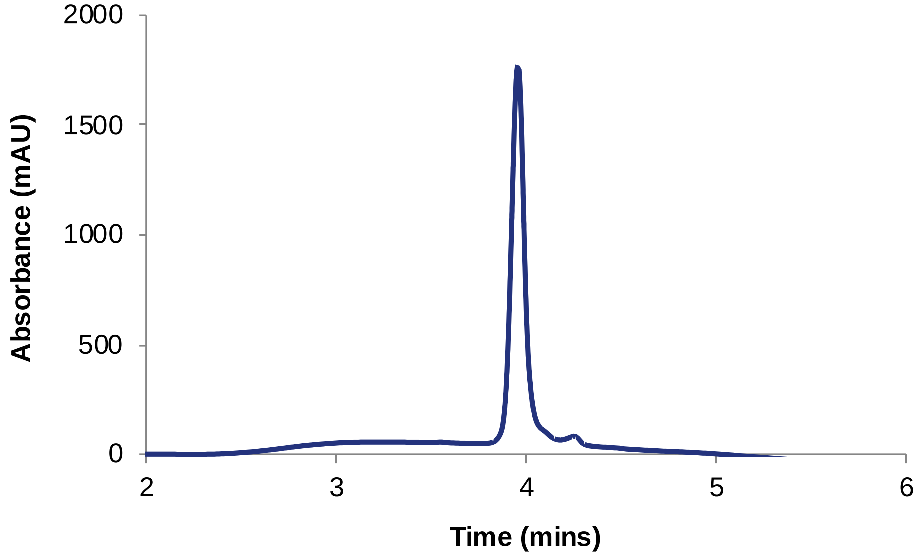

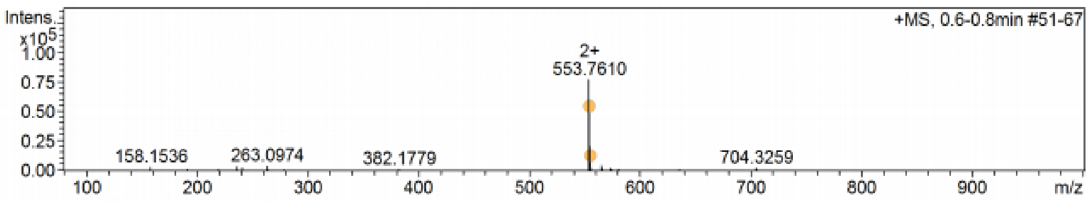

Figure S1. Analytical HPLC trace of purified Ac-CWHISKEY-NH2 (**1**). Analytical gradient 10-100% B over 5 min, 210 nm. Calculated Mass $[M+2H]^{2+}$: 553.76. Observed Mass $[M+2H]^{2+}$: 553.76.

Ac-CAY-NH2 (4a)

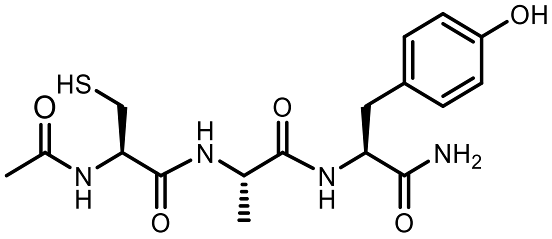

Peptide **4a** was synthesized using general automated synthesizer protocol with microwave assistance on rink amide resin (0.2 mmol). The crude peptide was purified by preparative RP-HPLC (5-60% B over 30 minutes) and lyophilized to produce the desired peptide (**4a**, 38 mg, 0.096 mmol, 48% yield).

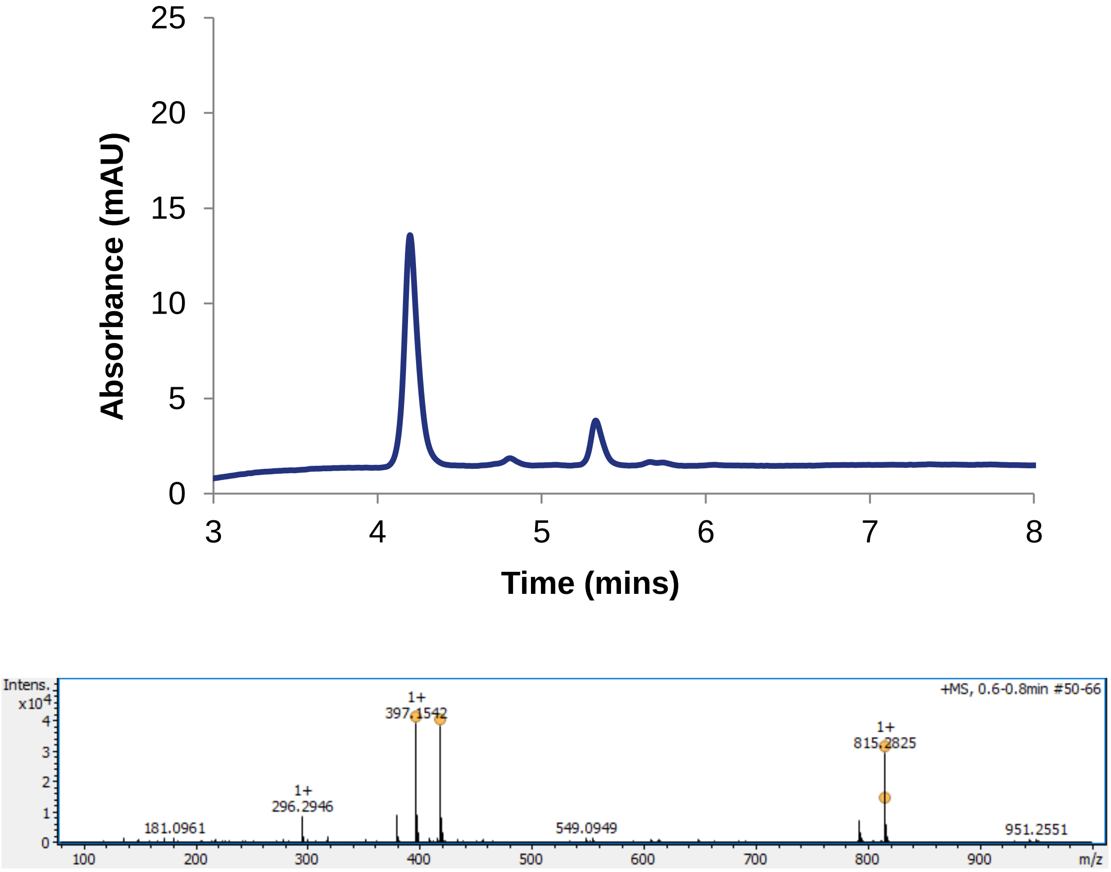

Figure S2 - Analytical HPLC trace of pure Ac-CAY-NH2 (**4a**). Analytical gradient 5-60% B over 10 min, 210 nm. Calculated Mass $[M+H+TFA]^+$: 397.15. Observed Mass $[M+H+TFA]^+$: 397.15.

Ac-D-CAY-NH2 (4b)

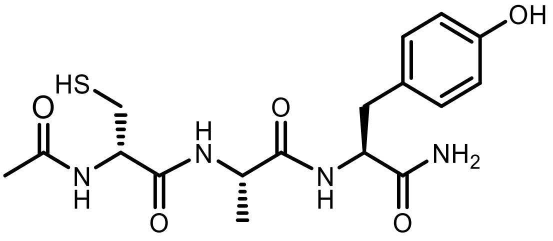

Peptide **4b** was synthesized using general automated synthesizer protocol with microwave assistance on rink amide resin (0.2 mmol). The crude peptide was purified by preparative RP-HPLC (5-60% B over 30 minutes) and lyophilized to produce the desired peptide (**4b**, 32 mg, 0.081 mmol, 41% yield).

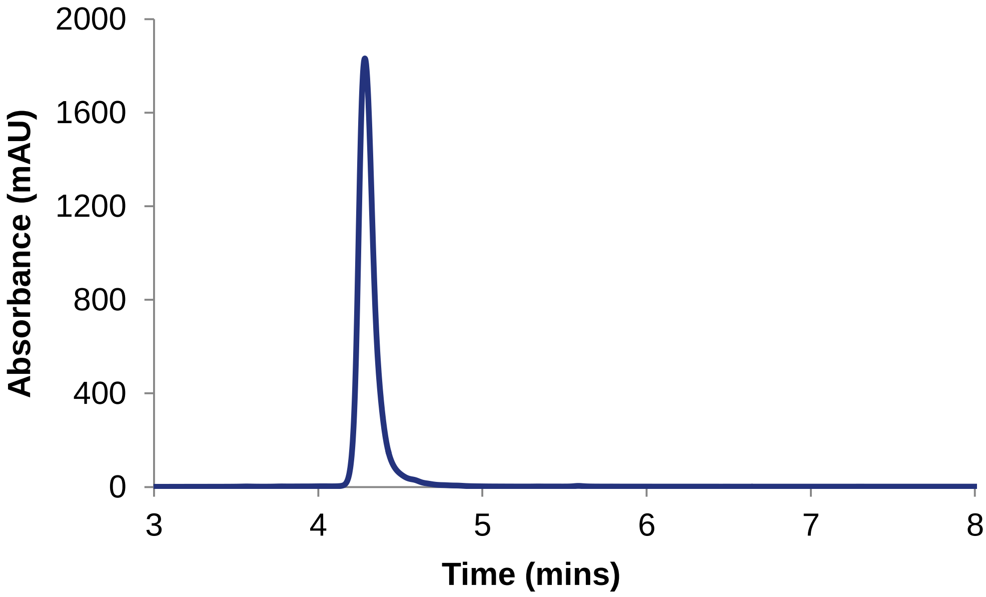

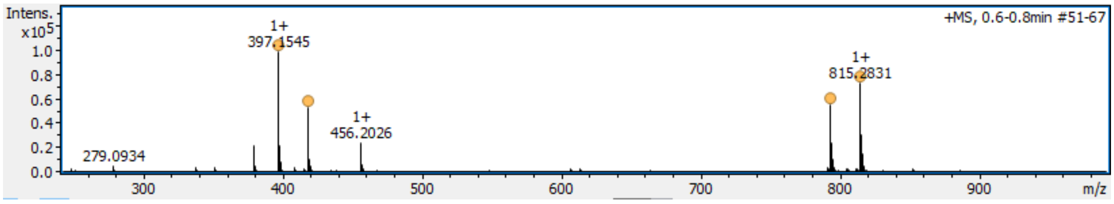

Figure S3 - Analytical HPLC trace of pure Ac-D-CAY-NH2 (**4b**). Analytical gradient 5-60% B over 10 min, 210 nm. Calculated Mass $[M+H]^+$: 397.15. Observed Mass $[M+H]^+$: 397.15.

Ac-L-K^{Ac}AY-NH$_2$ (5a)

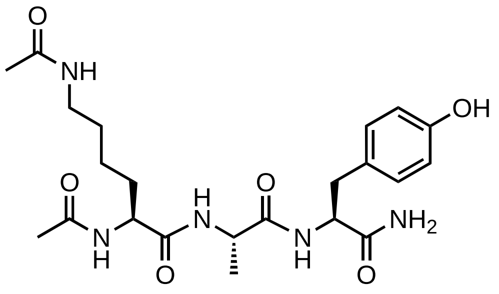

Peptide **5a** was synthesized using general automated synthesizer protocol with microwave assistance on rink amide resin (0.125 mmol). The crude peptide was purified by preparative RP-HPLC (5-60% B over 30 minutes) and lyophilized to produce the desired peptide (**5a**, 37.8 mg, 0.08 mmol, 81% yield).

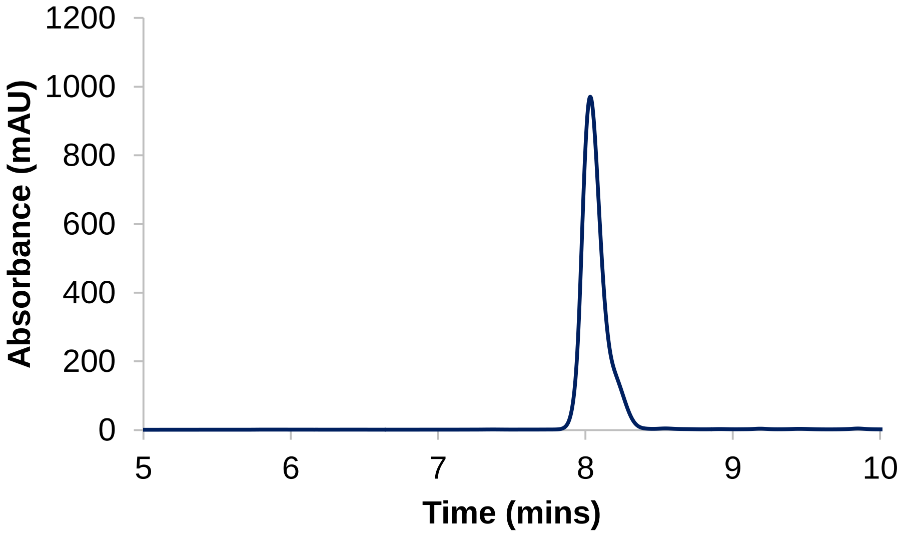

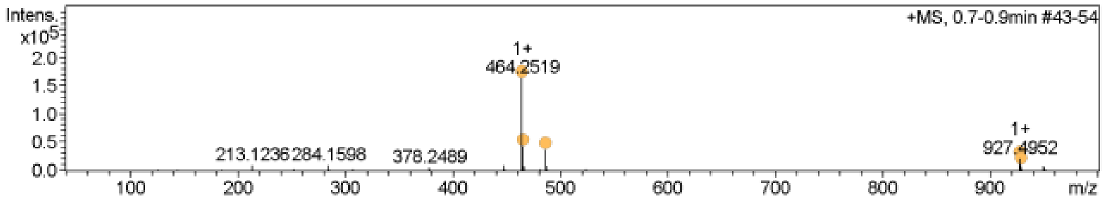

Figure S4 - Analytical HPLC trace of pure Ac-L-K^{Ac}AY-NH$_2$ (**5a**). Analytical gradient 2-25% B over 10 min, 280 nm. Calculated Mass [M+H]$^+$: 464.24. Observed Mass [M+H]$^+$: 464.25.

Ac-YECPLAHISCKY-NH_2 (6a)

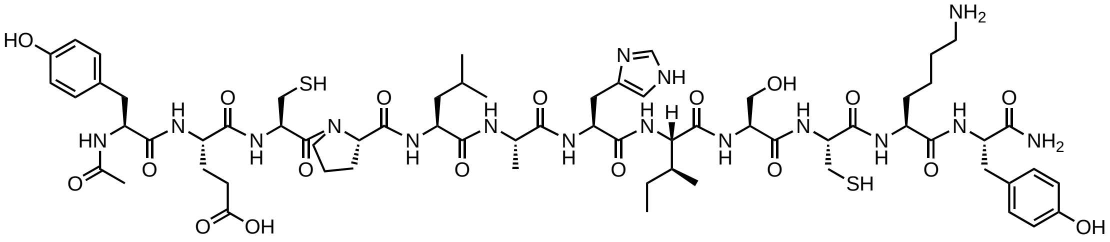

Peptide **6a** was synthesized using general automated synthesizer protocol with microwave assistance on rink amide resin (0.2 mmol). The crude peptide was purified by preparative RP-HPLC (15-80% B over 30 minutes) and lyophilized to produce the desired peptide (**6a**, 76.2 mg, 0.05 mmol, 52% yield).

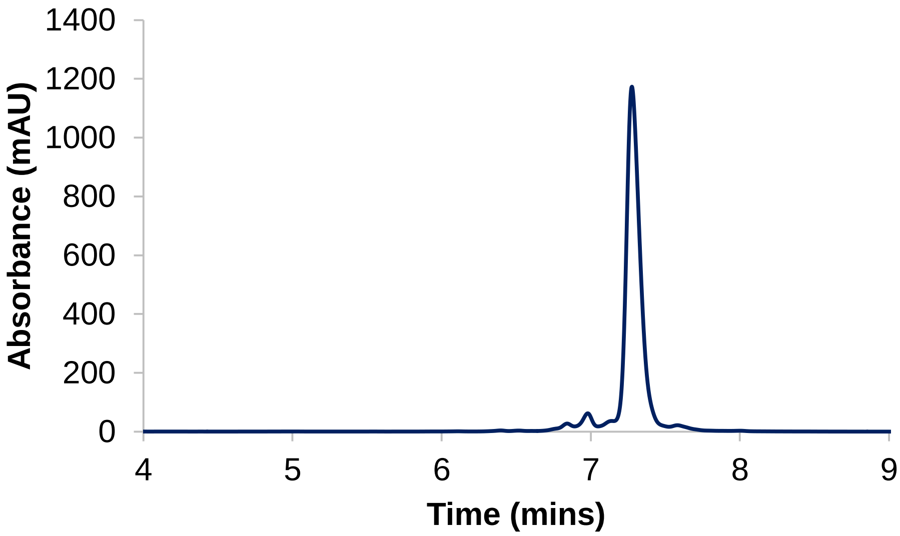

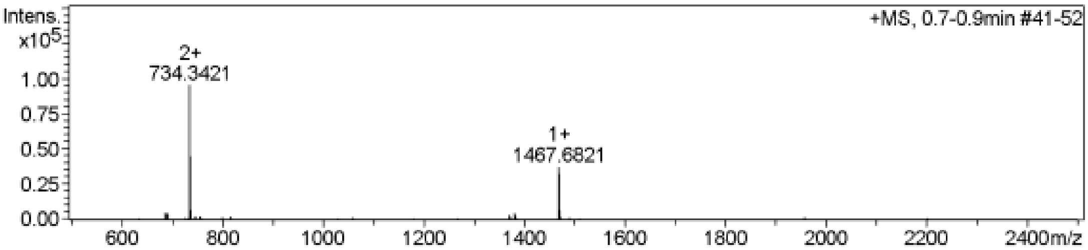

Figure S5 - Analytical HPLC trace of purified Ac-YECPLAHISCKEY-NH_2 (**6a**). Analytical gradient 5-60% B over 10 min, 280 nm. Calculated Mass $[M+H]^+$: 1467.67; Observed Mass $[M+H]^+$: 1467.68.

Ac-KCAY-NH2 (7)

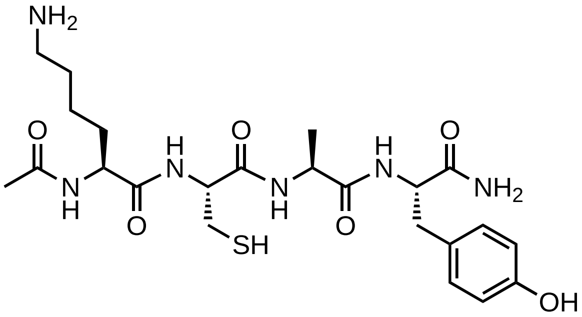

Peptide **7** was synthesized using general automated synthesizer protocol with microwave assistance on rink amide resin (0.125 mmol). The crude peptide was purified by preparative RP-HPLC (5-60% B over 30 minutes) and lyophilized to produce the desired peptide (**7**, 32.1 mg, 0.06 mmol, 49% yield).

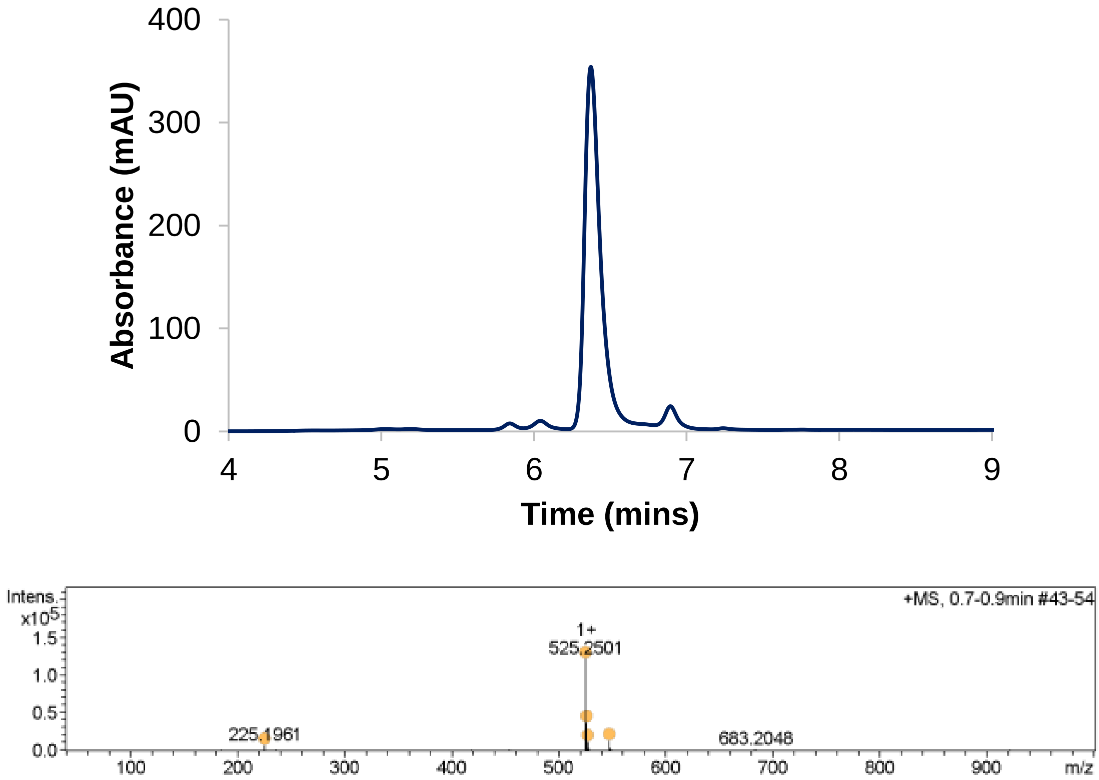

Figure S6 - Analytical HPLC trace of purified Ac-KCAY-NH2 (**7**). Analytical gradient 2-40% B over 10 min, 280 nm. Calculated Mass $[M+H]^+$: 525.24 Observed Mass $[M+H]^+$: 525.25.

Ac-ECAY-NH2 (8)

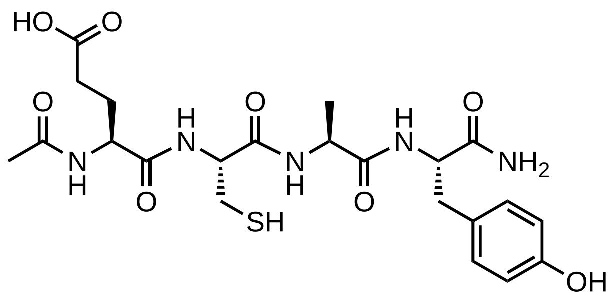

Peptide **8** was synthesized using general automated synthesizer protocol with microwave assistance on rink amide resin (0.125 mmol). The crude peptide was purified by preparative RP-HPLC (5-60% B over 30 minutes) and lyophilized to produce the desired peptide (**8**, 50.1 mg, 0.09 mmol, 76% yield).

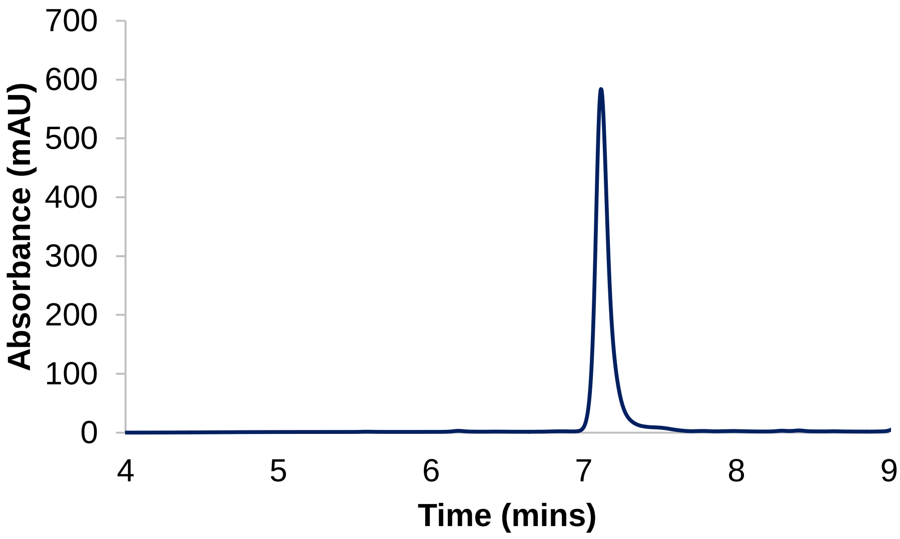

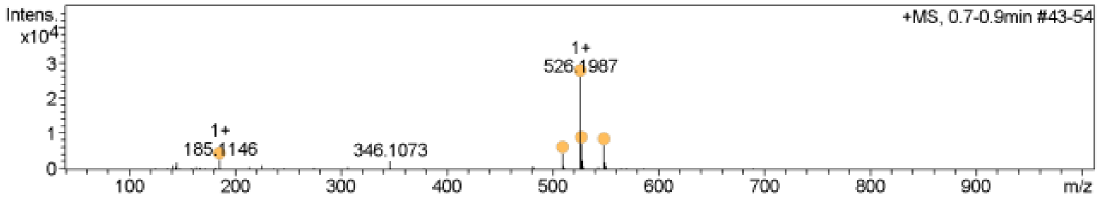

Figure S7 - Analytical HPLC trace of purified Ac-ECAY-NH2 (**8**). Analytical gradient 2-40% B over 10 min, 280 nm. Calculated Mass $[M+H]^+$: 526.19; Observed Mass $[M+H]^+$: 526.19.

Ac-YCAY-NH2 (9)

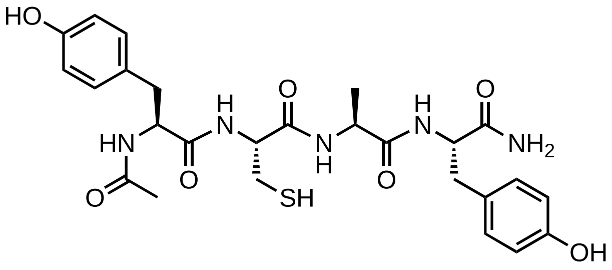

Peptide **9** was synthesized using general automated synthesizer protocol with microwave assistance on rink amide resin (0.125 mmol). The crude peptide was purified by preparative RP-HPLC (5-60% B over 30 minutes) and lyophilized to produce the desired peptide (**9**, 51.0 mg, 0.09 mmol, 73% yield).

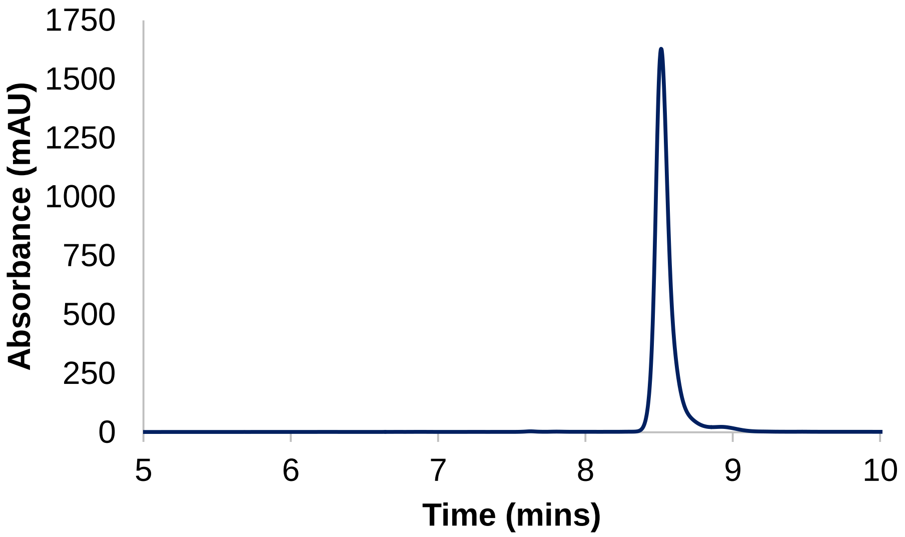

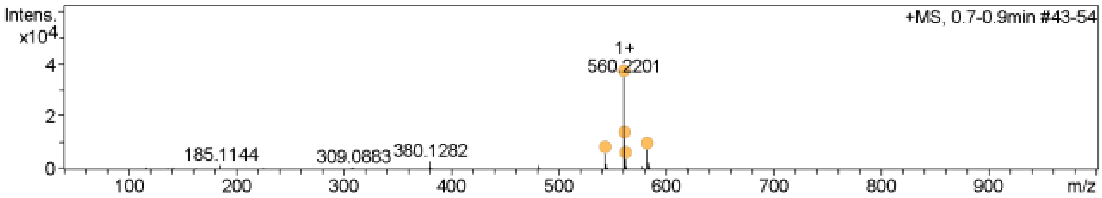

Figure S8 - Analytical HPLC trace of purified Ac-YCAY-NH2 (**9**). Analytical gradient 2-40% B over 10 min, 280 nm. Calculated Mass $[M+H]^+$: 560.21; Observed Mass $[M+H]^+$: 560.22.

Ac-ICAY-NH2 (10)

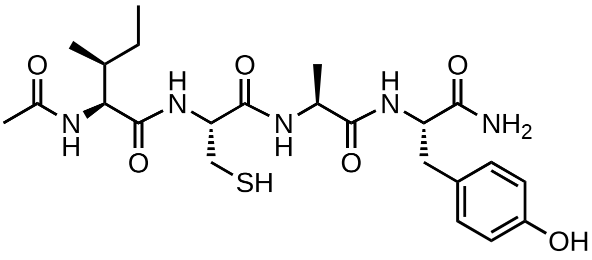

Peptide **10** was synthesized using general automated synthesizer protocol with microwave assistance on rink amide resin (0.125 mmol). The crude peptide was purified by preparative RP-HPLC (5-60% B over 30 minutes) and lyophilized to produce the desired peptide (**10**, 43 mg, 0.08 mmol, 68% yield).

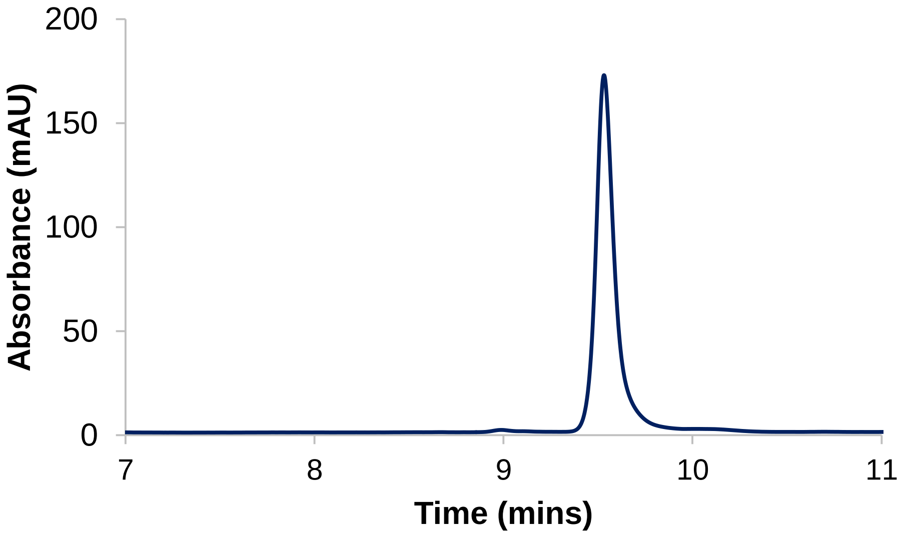

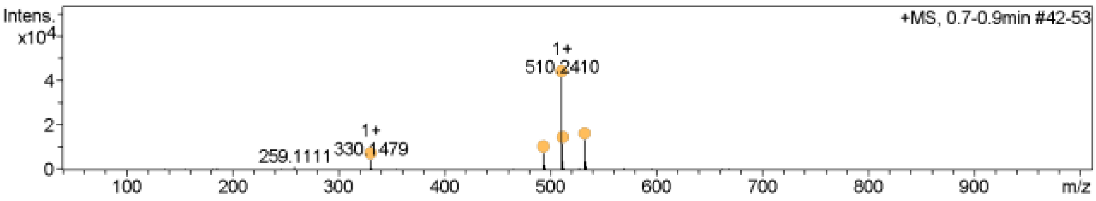

Figure S9 - Analytical HPLC trace of purified Ac-ICAY-NH2 (**10**). Analytical gradient 2-40% B over 10 min, 280 nm. Calculated Mass [M+H]$^+$: 510.25; Observed Mass [M+H]$^+$: 510.24.

H-HRLLRCGNYA-NH2 (23)

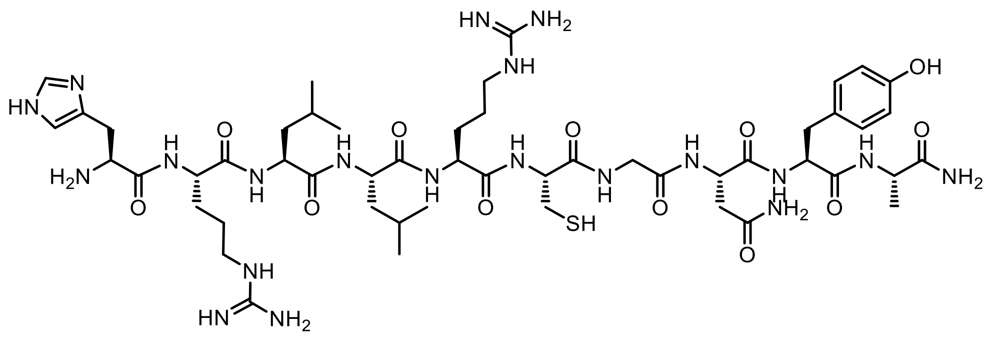

Peptide **23** was synthesized using general automated synthesizer protocol with microwave assistance on rink amide resin (0.2 mmol). The crude peptide was purified by preparative RP-HPLC (5-40% B over 30 minutes) and lyophilized to produce the desired peptide (**23**, 50 mg, 0.042 mmol, 21% yield).

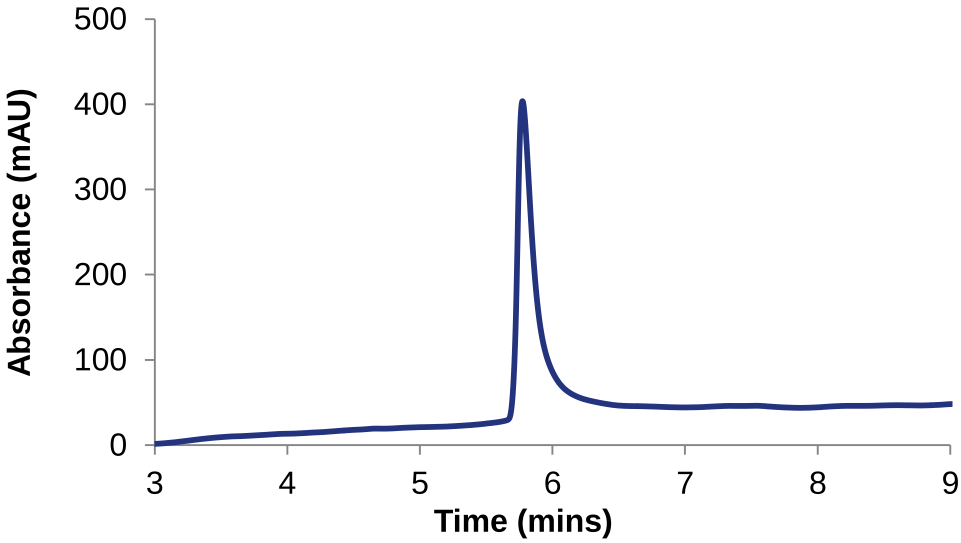

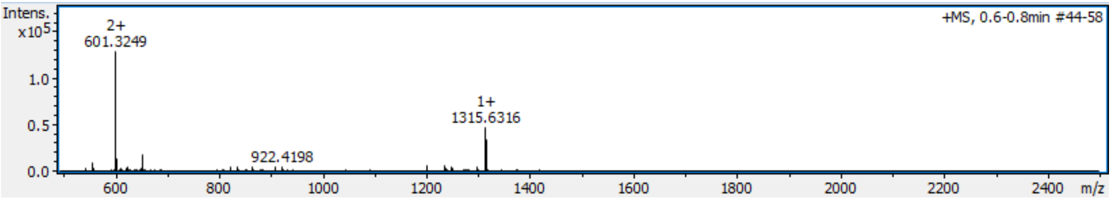

Figure S10 - Analytical HPLC trace of pure H-HRLLRCGNYA-NH_2 (**23**). Analytical gradient 10-30% B over 10 min, 210 nm. Calculated Mass $[M+H+TFA]^+$: 1315.63, $[M+2H]^{2+}$: 601.32. Observed Mass $[M+H+TFA]^+$: 1315.63, $[M+2H]^{2+}$: 401.22.

c[WHISCKEY] (33)

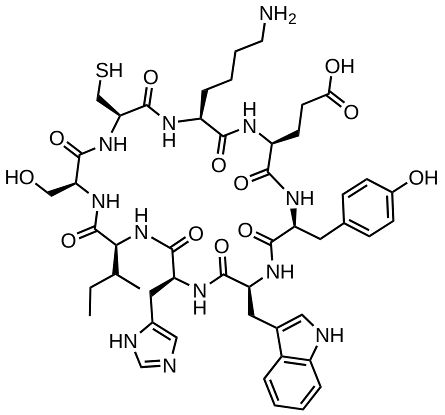

Peptide **33** was synthesized using general automated synthesizer protocol with microwave assistance on CTC resin (0.2 mmol). The peptide was then cleaved from the resin using 30% HFIP in DCM and cyclized using DMTMM, DIPEA in DMF. The crude peptide was purified by preparative RP-HPLC (15-60% B over 30 minutes) and lyophilized to produce the desired peptide (**33**, 21.8 mg, 0.02 mmol, 19% yield).

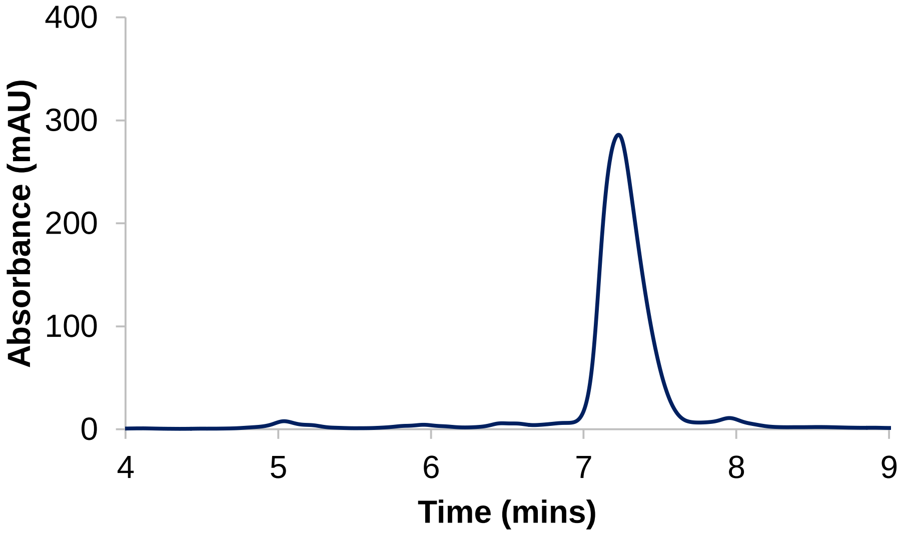

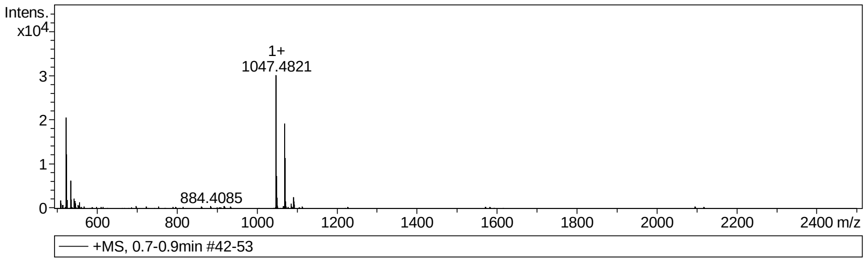

Figure S11 - Analytical HPLC trace of purified c[WHISCKEY] (**33**). Analytical gradient 15-25% B over 10 min, 280 nm. Calculated Mass $[M+H]^+$: 1047.47; Observed Mass $[M+H]^+$: 1047.48.

Initial optimization of visible-light-mediated desulfurative C(sp^3)-C(sp^3) bond formation

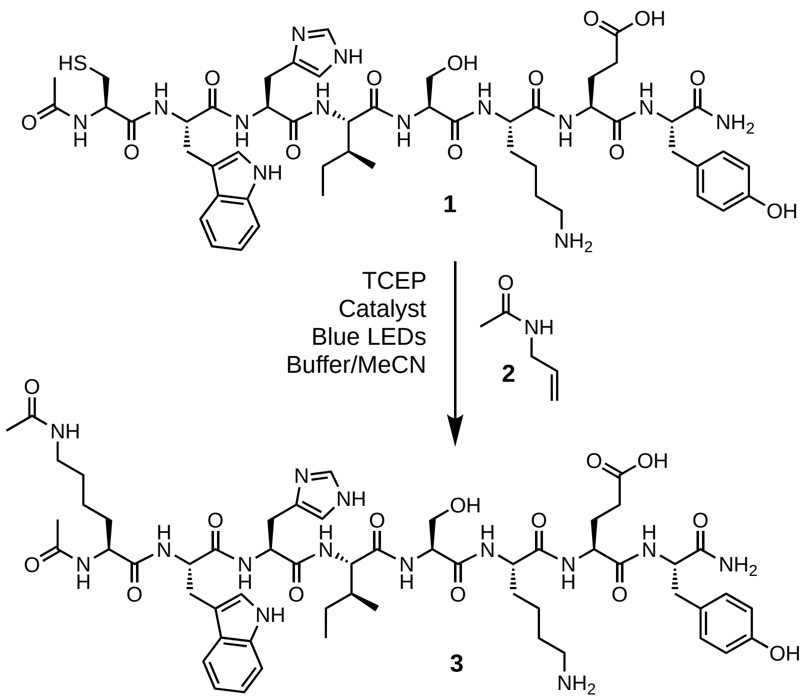

Table 1 - Initial allyl conjugation reactions using the model peptide Ac-CWHISKEY-NH_2. Peptide – 1 mM, TCEP – 50 mM, 20% DMSO in LB at pH 7 for 2 hours.

Trial	N-allyl acetamide eq.	Initiator (eq.)	Product %conv.	Ala %conv.	SM %rem.	MA %conv.
1	500	Ir (0.01)	36	54	0	10
2	500	EY (0.1)	37	49	0	13
3	10	EY (0.1)	0	100	0	0
4	500	Mn (5)	28	72	0	0
5	10	Mn (5)	0	100	0	0
6	2	Mn (5)	0	100	0	0

Table 2 – Peptide concentration optimisation using 50 eq. TCEP and 500 eq. N-allyl acetamide in 20% DMSO in LB at pH 7 for 2 hours.

Trial	Peptide conc. mM	Initiator (eq.)	Product %conv.	Ala %conv.	SM %rem.	MA %conv.
7	5	EY (0.1)	7	23	0	70
8	2.5	EY (0.1)	28	48	0	24
9	1	EY (0.1)	37	49	0	13
10	0.5	EY (0.1)	38	45	0	16
11	0.2	EY (0.1)	35	56	0	9
12	5	Ir (0.01)	17	30	0	53
13	2.5	Ir (0.01)	27	38	0	35
14	1	Ir (0.01)	30	40	0	30
15	0.5	Ir (0.01)	43	49	0	9
16	0.2	Ir (0.01)	39	51	0	10

Table 3 - TCEP optimisation using 0.5 mM peptide, 500 eq. N-allyl acetamide and either 10 mol% eosin Y or 1 mol% Ir(III) in 20% DMSO in LB at pH 7 for 2 hours.

Trial	TCEP eq.	Initiator (eq.)	Product %conv.	Ala %conv.	SM %rem.	MA %conv.
17	100	EY (0.1)	33	58	0	9
18	50	EY (0.1)	38	45	0	16
19	10	EY (0.1)	42	37	0	21
20	5	EY (0.1)	44	35	0	21
21	100	Ir (0.01)	0	14	0	86
22	50	Ir (0.01)	30	40	0	30
23	10	Ir (0.01)	42	39	0	19
24	5	Ir (0.01)	42	40	0	17

Table 4 - pH optimisation using 0.5 mM peptide, 5 eq. TCEP, 500 eq. N-allyl acetamide and either 10 mol% eosin Y or 1 mol% Ir in 20% DMSO in LB at pH 7 for 4 hours.

Trial	pH	Initiator (eq.)	Product %conv.	Ala %conv.	SM %rem.	MA %conv.
25	5	EY (0.1)	27	40	0	33
26	6	EY (0.1)	33	47	0	20
27	7	EY (0.1)	41	43	0	17
28	8	EY (0.1)	42	41	0	17
29	9	EY (0.1)	35	48	0	17
30	5	Ir (0.01)	0	0	100	0
31	6	Ir (0.01)	15	38	46	0
32	7	Ir (0.01)	42	40	0	17
33	8	Ir (0.01)	52	35	0	13
34	9	Ir (0.01)	40	27	0	33

Table 5 - Initiator equivalents optimisation. Peptide 0.5 mM, 5 eq. TCEP, 500 eq. N-allyl acetamide. Eosin Y reactions at pH 7 and Ir reactions at pH 8. Solvent used was 20% DMSO in LB. Reactions analysed after 1 and 4 hours.

Trial	Initiator (eq.)	Product %conv.	Ala %conv.	SM %rem.	MA %conv.	Completion time
35	EY (0.1)	48	36	0	17	4 hours
36	EY (0.5)	50	32	0	18	1 hour
37	EY (0.02)	38	36	0	26	4 hours
38	Ir (0.01)	48	33	0	19	4 hours
39	Ir (0.05)	51	29	0	20	1 hour
40	Ir (0.001)	20	21	59	0	>4 hours

Table 6 - N-allyl acetamide eq. optimisation. 0.5 mM peptide, 5 eq. TCEP. Either 50 mol% eosin Y at pH 7 or 5 mol% Ir at pH 8. Reactions analysed after 1 hour.

Trial	N-allyl acetamide eq.	Initiator (eq.)	Product %conv.	Ala %conv.	SM %rem.	MA %conv.
41	1000	EY (0.5)	38	24	14	24
42	500	EY (0.5)	48	32	0	20
43	200	EY (0.5)	45	44	0	12
44	100	EY (0.5)	38	55	0	7
45	1000	Ir (0.05)	41	28	8	24
46	500	Ir (0.05)	51	30	0	19
47	200	Ir (0.05)	49	38	0	12
48	200	Ir (0.1)	49	43	0	7
49	100	Ir (0.05)	43	51	0	6

Table 7 - Trials at reducing the organic solvent composition. Conditions - 0.5 mM peptide, 5 eq. TCEP and 200 eq. N-allyl acetamide. First two trials used 5 mol% Ir catalyst at pH 8. Last trial using 50 mol% eosin Y at pH 7 for 1 hour.

Trial	DMSO %	Product %conv.	Ala %conv.	SM %rem.	MA %conv.
50	5	27	32	34	7
51	10	45	41	0	14
52	0	45	45	0	10

The optimal reaction conditions, taking into account percentage conversion and reagent equivalents, were designated as follows using photochemistry set up 1 (blue LEDs) – 0.5 mM peptide, 5 eq. TCEP, 200 eq. allyl, 5 mol% Ir catalyst at pH 8 for 1 hour; referred to as protocol A.

Installation of N^{ε}-acetyl on an isolatable scale

Ac-CWHISKEY-NH2 (1) + *N*-allyl acetamide (2) – photochemistry set up 1

The optimized conditions (protocol A) were applied to an isolatable scale reaction on Ac-CWHISKEY-NH2 (**1**) with *N*-allyl acetamide (**2**) using set up 1 to ascertain the overall isolated yield and reaction rate. The reaction was monitored at 15-minute intervals and was shown to reach completion after 45 minutes. At this time, the conversion of starting material to product was 55%. Isolated of the desired product by preparative HPLC afforded peptide **3a** in 51% yield.

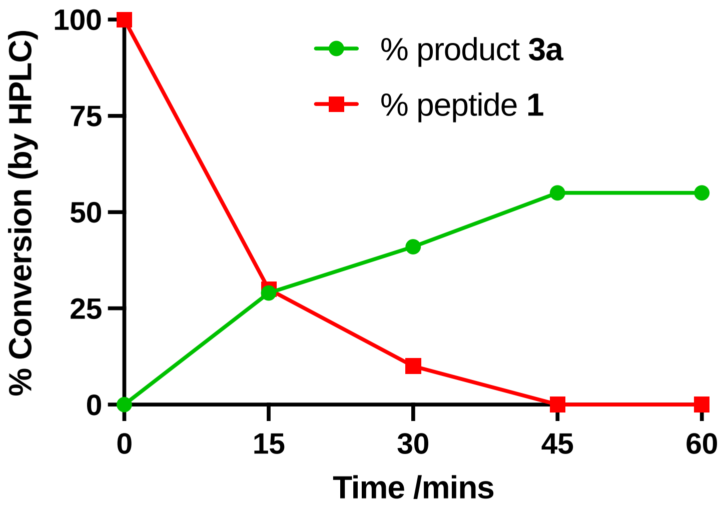

Figure S12 – Rate analysis for the conjugation of Ac-CWHISKEY-NH2 **1** with N-allyl acetamide **2** using general protocol A, set up 1.

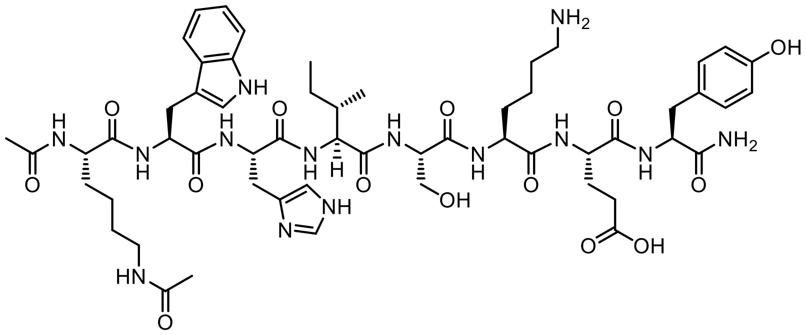

Peptide **3a** was synthesised following general conjugation protocol A (set up 1) using Ac-CWHISKEY-NH2 (**1**, 2 mg, 1.66 µmol) and *N*-allyl acetamide (**2**, 33 mg, 0.33 mmol), purified by semi preparative HPLC (15-80%B over 30 minutes) to produce the desired conjugated peptide (**3a**, 1.0 mg, 0.852 µmol, 51% yield).

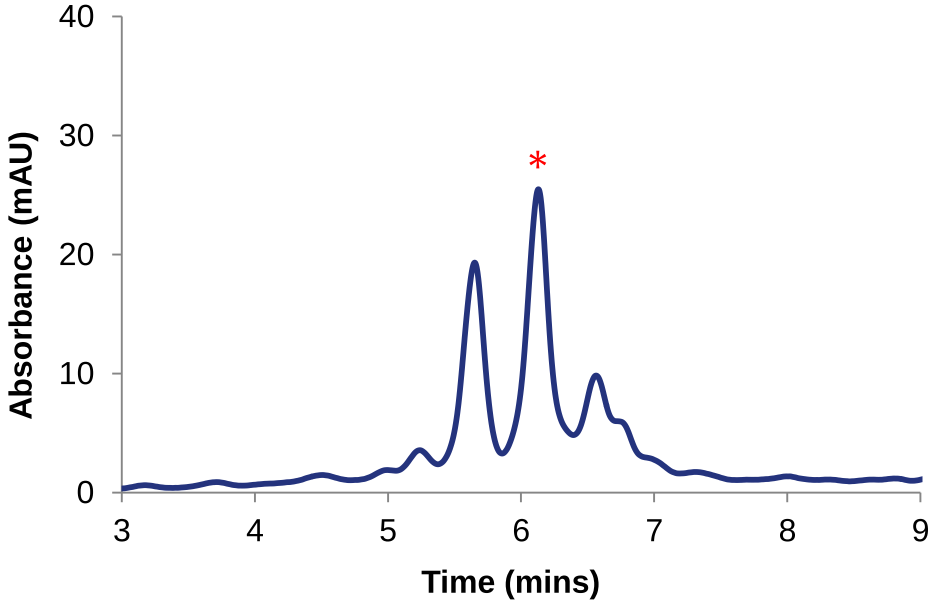

Figure S13 - Analytical HPLC trace of the crude reaction between Ac-CWHISKEY-NH2 (**1**) + *N*-allyl acetamide (**2**) using protocol A under blue LEDs (photochemical set up 1); desired product identified. Analytical gradient 15-25% B over 10 min, 280 nm.

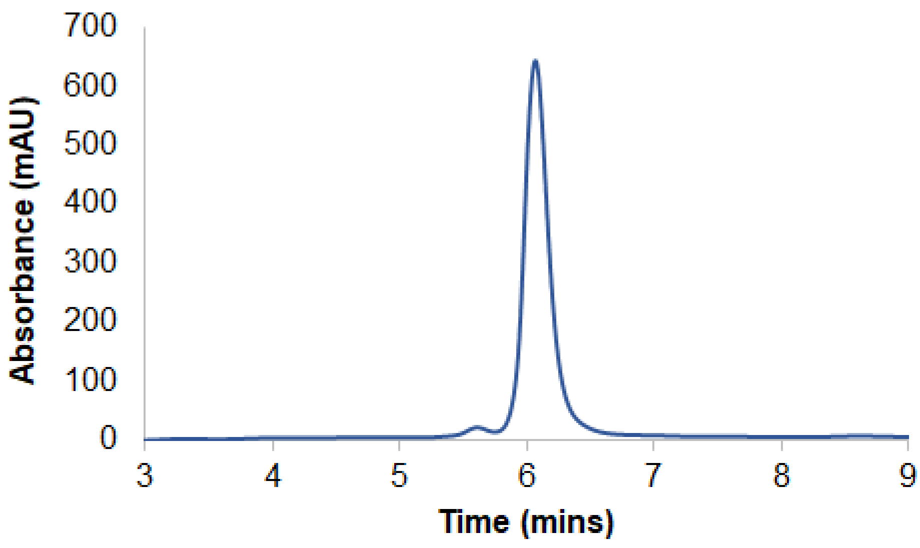

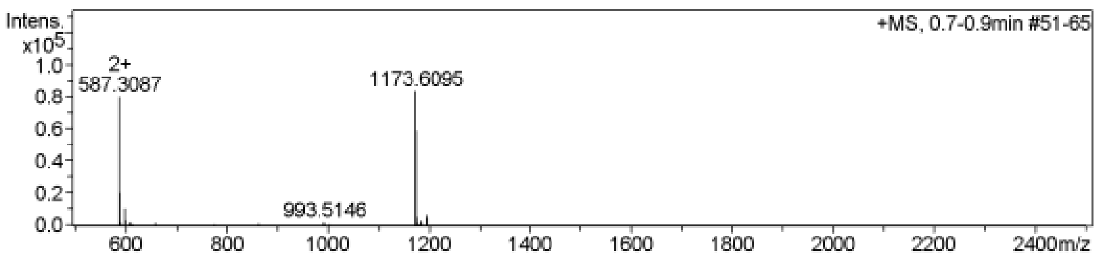

Figure S14 - Analytical HPLC trace of pure product **3a**. Analytical gradient 15-25% B over 10 min, 280 nm. Calculated Mass $[M+H]^{+}$: 1173.61, $[M+2H]^{2+}$: 587.31. Observed Mass $[M+H]^{+}$: 1173.61, $[M+2H]^{2+}$: 587.31.

Ac-CWHISKEY-NH2 (1) + *N*-allyl acetamide (2) – photochemistry set up 2

Protocol A was then applied to Ac-CWHISKEY-NH2 (**1**) with *N*-allyl acetamide (**2**) on an analytical scale using set up 2 (PhotoRedOx Box) to ascertain the reaction rate using this specialized equipment. Aliquots were taken in one-minute intervals and analyzed by analytical HPLC. The reaction was observed to reach full consumption of the starting peptide **1** after just 2 minutes in comparable conversion to the reaction run using set up 1 (blue LEDs). To confirm the endpoint, a 2-minute irradiation was repeated, and the sample immediately injected into the HPLC. This reaction was repeated on a preparative scale to determine the isolated yield.

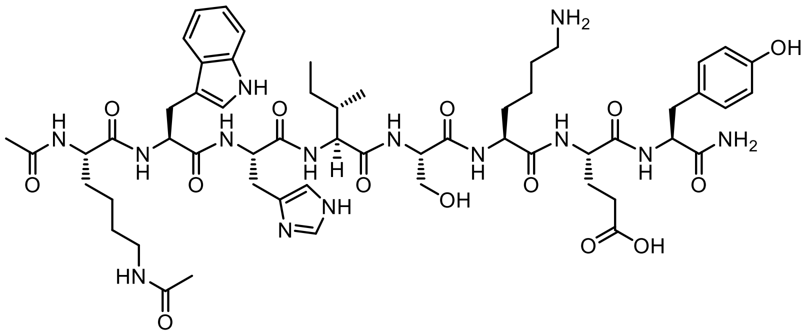

Peptide **3a** was synthesised following general conjugation protocol A (set up 2) using Ac-CWHISKEY-NH2 (**1**, 2 mg, 1.81 µmol) and *N*-allyl acetamide (**2**, 36 mg, 0.36 mmol), purified by semi preparative HPLC (15-60%B over 60 minutes) to produce the desired conjugated peptide (**3a**, 0.9 mg, 0.77 µmol, 54% yield).

This reaction was repeated in degassed solvents and also in a glove box with degassed solvents – no change in the ratio of product:by-products was observed.

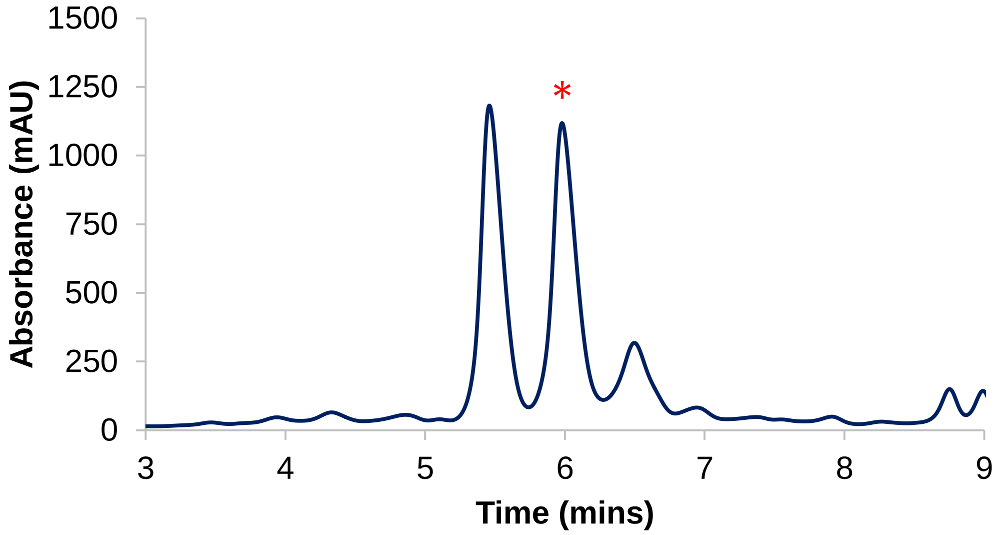

Figure S15 - Analytical HPLC trace of the crude reaction between Ac-CWHISKEY-NH_2 (**1**) + *N*-allyl acetamide (**2**) using protocol A in the PhotoRedOx Box (photochemical set up 2); desired product identified. Analytical gradient 15-25% B over 10 min, 280 nm.

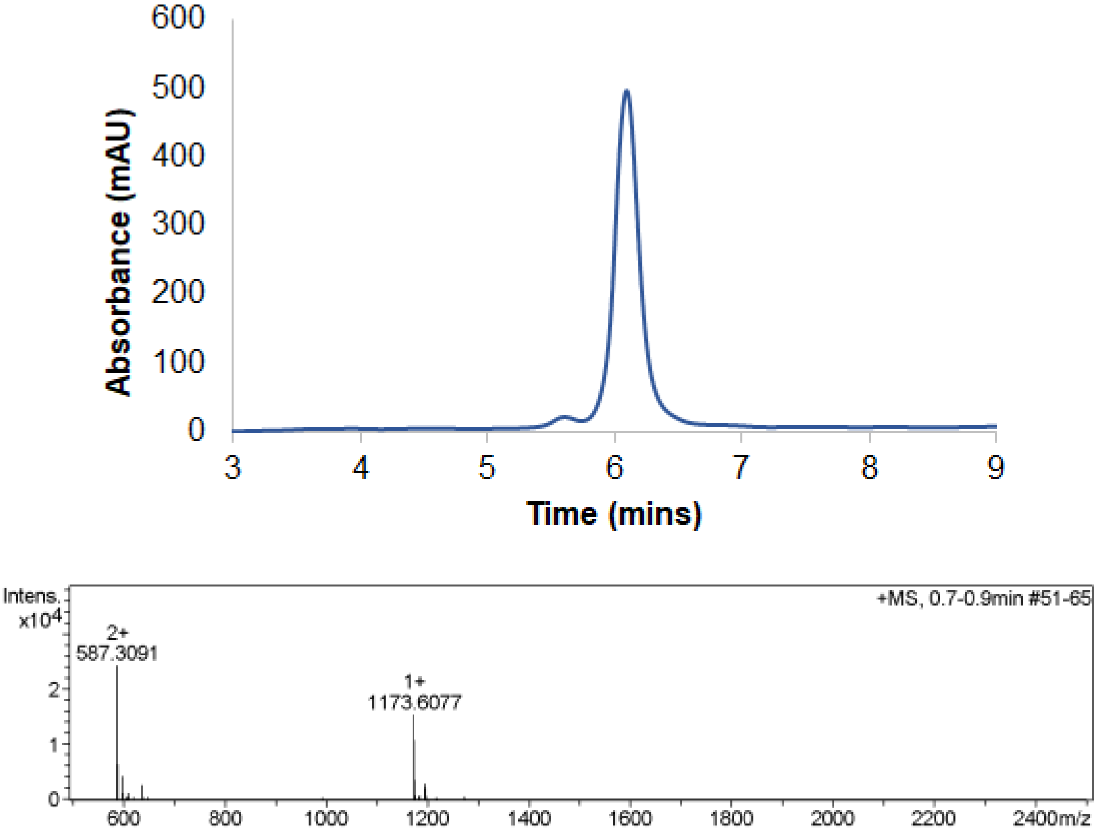

Figure S16 - Analytical HPLC trace of pure product **3a**. Analytical gradient 15-25% B over 10 min, 210 nm. Calculated Mass $[M+H]^{+}$: 1173.61, $[M+2H]^{2+}$: 587.31. Observed Mass $[M+H]^{+}$: 1173.61, $[M+2H]^{2+}$: 587.31.

Investigation of retention of stereochemistry at the modified residue

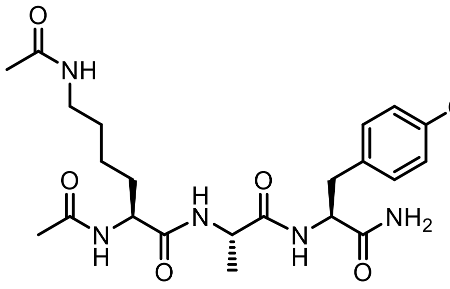

Peptide **5a** was synthesized following the general conjugation protocol A (set up 1) using Ac-CAY-NH_2 (**4a**, 4 mg, 10.1 µmol) and *N*-allyl acetamide (**2**, 200 mg, 2 mmol), purified using semi-preparative HPLC (2-60% B over 30 minutes) to yield the desired conjugated peptide (**5a**, 2.6 mg, 5.61 µmol, 56% yield). ^{1}H NMR (500 MHz, MeOD) δ 8.25 (d, J = 6.1 Hz, 1H), 7.78 (d, J = 8.0 Hz, 1H), 7.07 (d, J = 8.5 Hz, 2H), 6.71 (d, J = 8.5 Hz, 2H), 4.52 – 4.47 (m, 1H), 4.28 – 4.20 (m, 2H), 3.19 – 3.16 (m, 2H), 3.08 (dd, J = 14.0, 5.6 Hz, 1H), 2.92 (dd, J = 14.0, 8.5 Hz, 1H), 2.02 (s, 3H), 1.95 (s, 3H), 1.79 – 1.60 (m, 2H), 1.53 (q, J = 7.2 Hz, 2H), 1.39 (m, 2H), 1.29 (d, J = 7.2 Hz, 3H).^{13}C NMR (125 MHz, MeOD) δ 174.7 (C), 173.4 (C), 173.3 (C), 172.3 (C), 171.9 (C), 155.9 (Ar), 129.9 (Ar), 127.7 (Ar-H), 114.8 (Ar-H), 54.5 (CH), 53.8 (CH), 49.5 (CH), 38.8 (CH_2), 36.4 (CH_2), 31.0 (CH_3), 28.5 (CH_3), 22.8 (CH_2), 21.2 (CH_2), 21.1 (CH_2), 16.2 (CH_3).

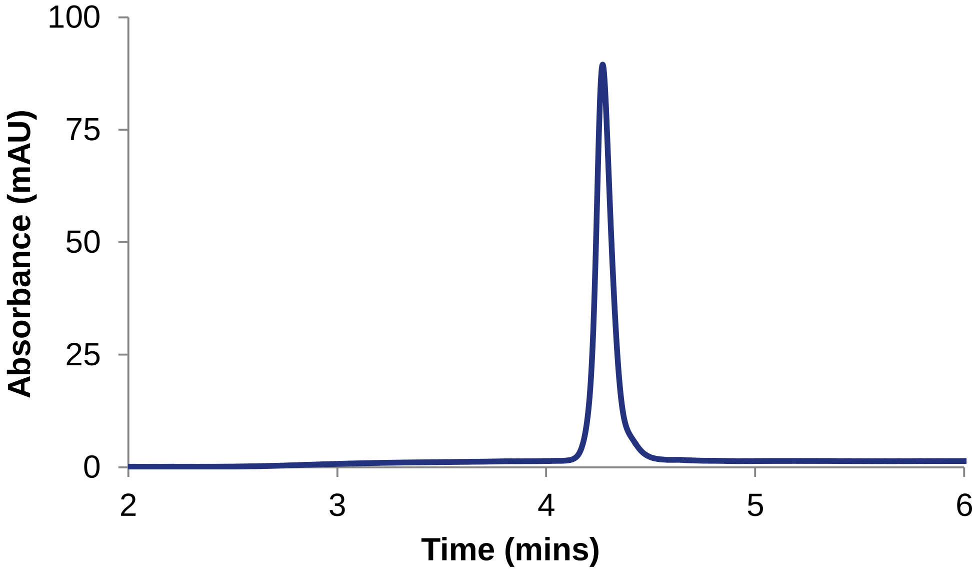

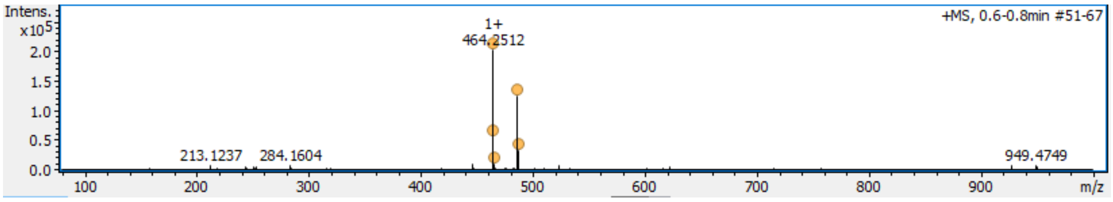

Figure S17 - Analytical HPLC trace of pure product **5a**. Analytical gradient 5-60% B over 10 min, 280 nm. Calculated Mass $[M+H]^+$: 464.25. Observed Mass $[M+H]^+$: 464.25.

Ac-D-CAY-NH2 modified with *N*-allyl acetamide

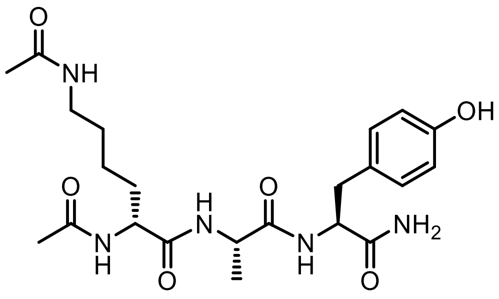

Peptide **5b** was synthesized following the general conjugation protocol A (set up 1) Ac-D-CAY-NH2 (**4b**, 4 mg, 10.1 µmol) and *N*-allyl acetamide (**2**, 200 mg, 2 mmol), purified using semi-preparative HPLC (2-60% B over 30 minutes) to yield the desired conjugated peptide (**5b**, 2.4 mg, 5.18 µmol, 51% yield). ^{1}H NMR (500 MHz, MeOD) δ 7.13 (d, J = 8.5 Hz, 2H), 6.71 (d, J = 8.5 Hz, 2H), 4.48 – 4.40 (m, 1H), 4.16 – 4.05 (m, 2H), 3.20 – 3.15 (m, 3H), 2.94 (dd, J = 13.9, 11.3 Hz, 1H), 2.00 (s, 3H), 1.94 (s, 3H), 1.73 – 1.70 (m, 1H), 1.55 – 1.53 (m, 2H), 1.49 – 1.28 (m, 3H), 1.20 (d, J = 7.3 Hz, 3H). ^{13}C NMR (125 MHz, MeOD) δ 175.4 (C), 175.0 (C), 173.6 (C), 172.5 (C), 171.9 (C), 155.8 (Ar), 129.8 (Ar), 128.4 (Ar-H), 114.7 (Ar-H), 55.0 (CH), 54.5 (CH), 50.3 (CH), 38.7 (CH_2), 35.8 (CH_2), 30.2 (CH_3), 28.7 (CH_3), 22.8 (CH_2), 21.1 (CH_2), 20.9 (CH_2), 15.6 (CH_3).

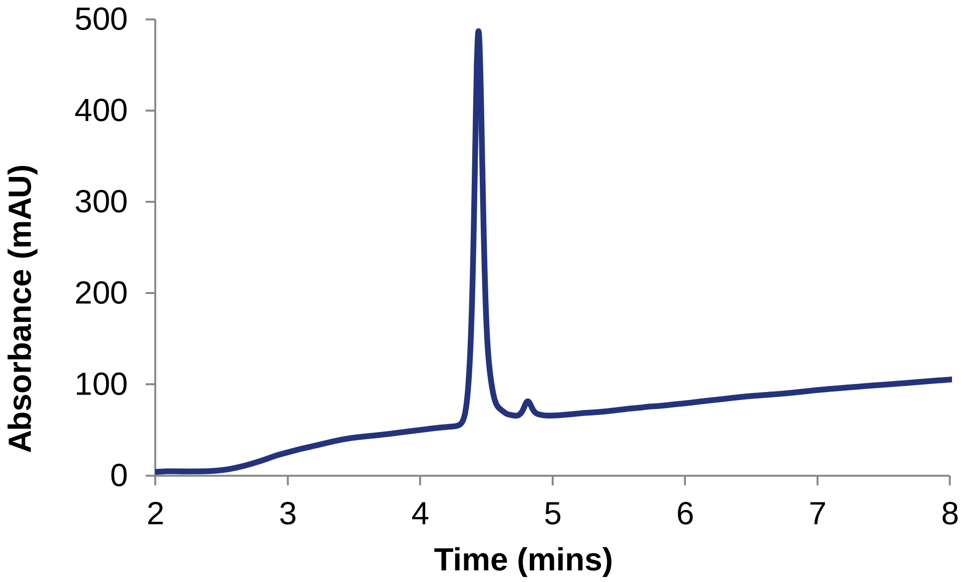

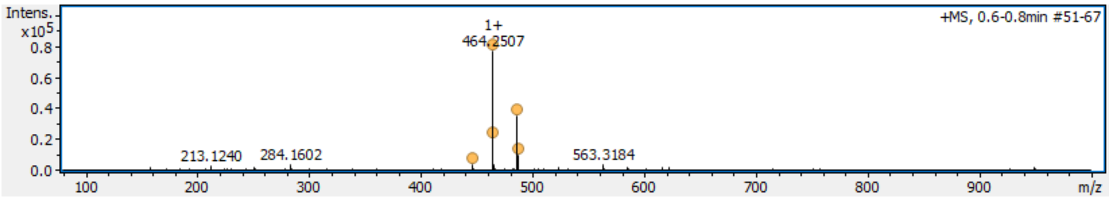

Figure S18 - Analytical HPLC trace of pure product **5b**. Analytical gradient 5-60% B over 10 min, 280 nm. Calculated Mass $[M+H]^+$: 464.25. Observed Mass $[M+H]^+$: 464.25.

NMR comparison of *N*-allyl acetamide modified Ac-CAY-NH_2 and Ac-D-CAY-NH_2 with chemically synthesized Ac-K(Ac)AY-NH_2

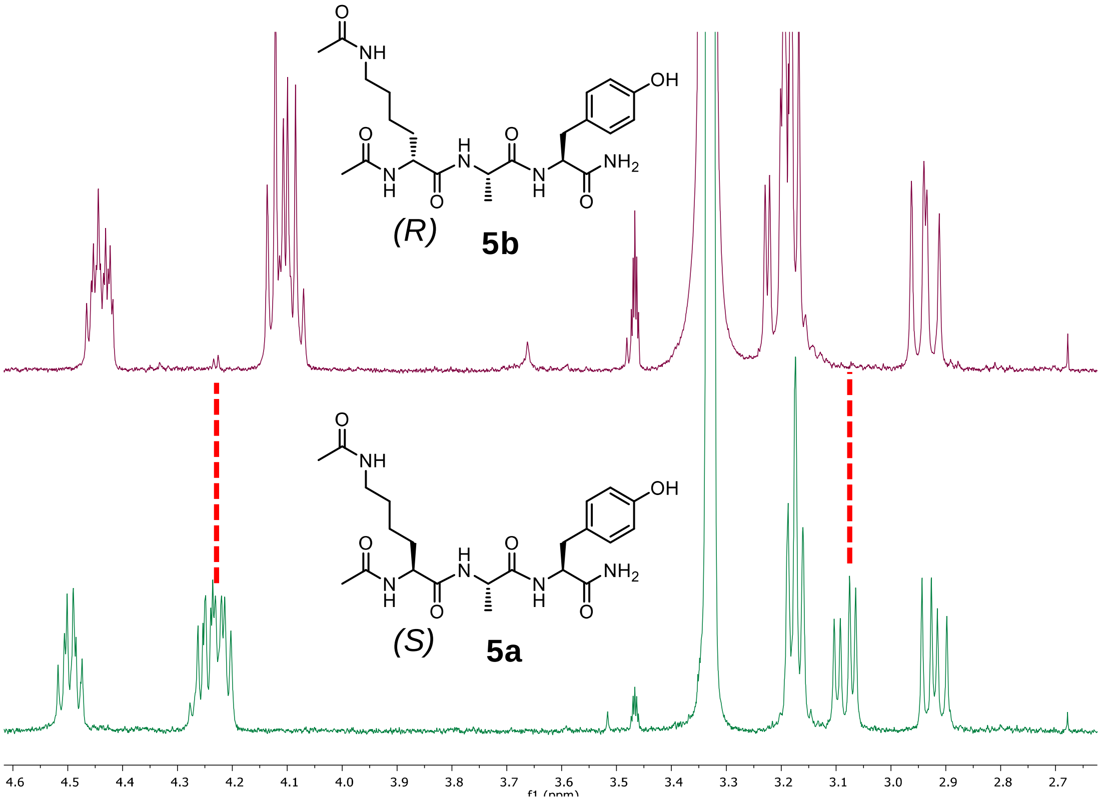

Figure S19 - NMR comparison of L (**5a**, green spectra) and D (**5b**, red spectra) modified peptides.

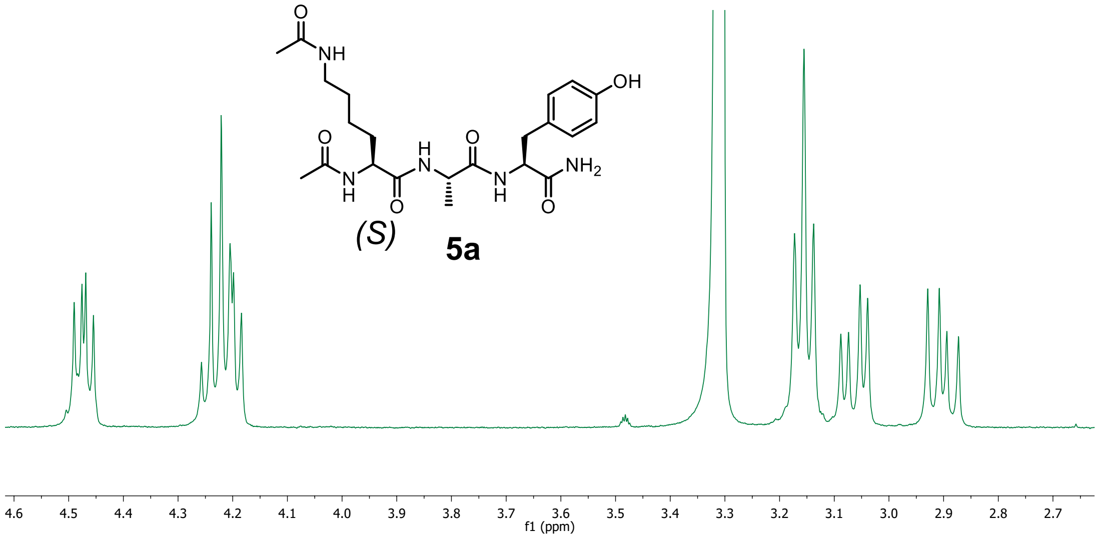

Figure S20 - NMR comparison of **5a** synthesized on the solid phase using Fmoc-L-Lys(Ac)-OH.

Modification of multiple Cys residues

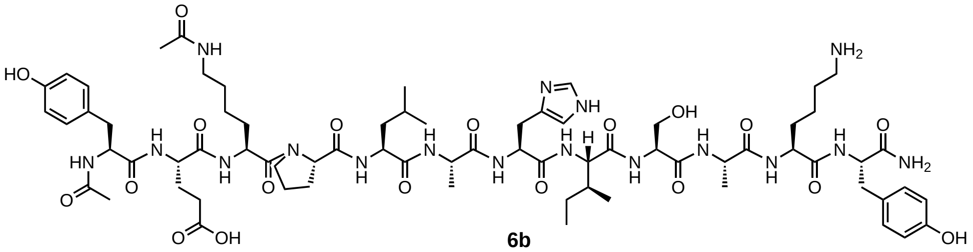

6b

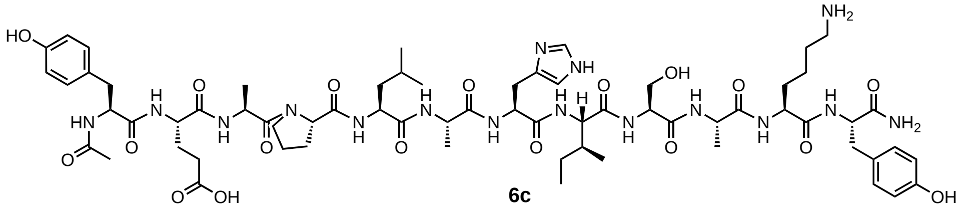

6c

Peptide **6a** (3 mg, 0.002 mmol) was subjected to protocol A (set up 2) using *N*-allyl acetamide (**2**, 33 mg, 0.33 mmol). The reaction yielded a crude mixture of products, primarily consisting of an inseparable mixture of peptides **6b** and **6c**.

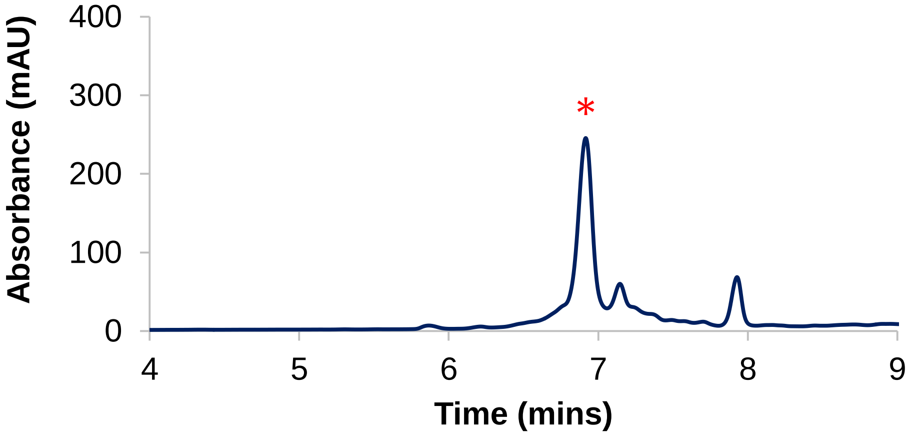

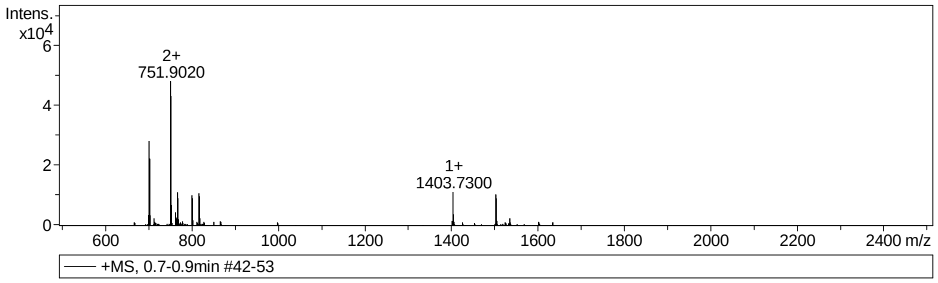

Figure S21 – Crude HPLC trace of modified Ac-YECPLAHISCKY-NH_2 (**6a**); analytical gradient 5-60% B over 10 min, 280 nm; MS of identified peak contains both peptides **6b** and **6c**; **6b** Calculated Mass $[M+H]^+$: 1502.79; Observed Mass $[M+H]^+$: 1502.81; **6c** Calculated Mass $[M+H]^+$: 1403.72; Observed Mass $[M+H]^+$: 1403.73.

Exploration of local chemical environment

Modification of Ac-KCAY-NH2 (7) with *N*-allyl acetamide (2)

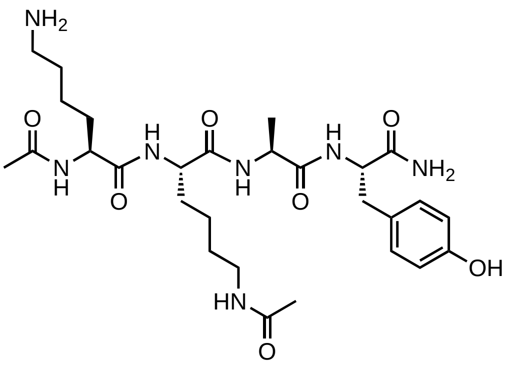

Peptide **11** was synthesized following the general conjugation protocol A (set up 2) using Ac-KCAY-NH2 (**7**, 2 mg, 3.82 µmol) and *N*-allyl acetamide (**2**, 75 mg, 0.76 mmol). The % conversion to the desired product **11** was calculated from the analytical HPLC of the crude reaction mixture. After analysis the remainder of material (3.76 µmol) was purified using purified using semi-preparative HPLC (5-60% B over 30 minutes) to yield the desired conjugated peptide (**11**, 0.5 mg, 0.85 µmol, 23% yield).

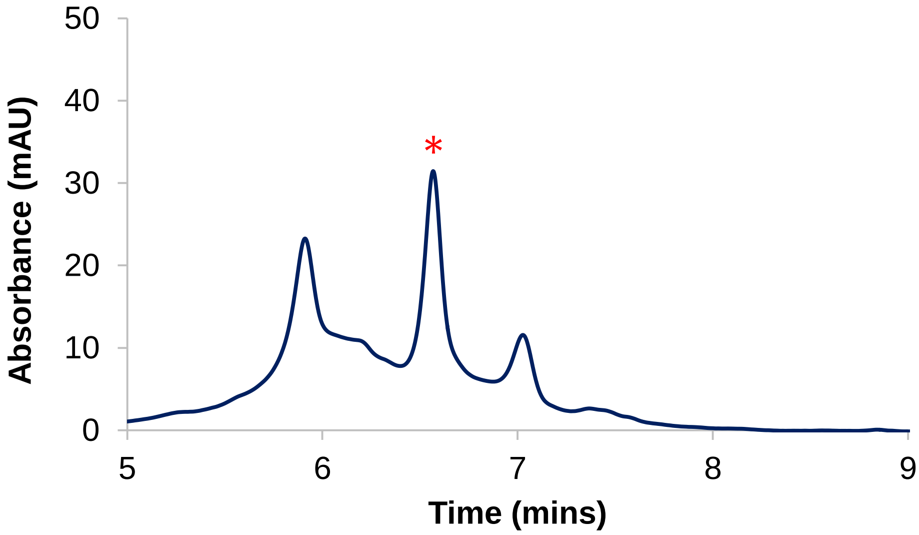

Figure S22 – Crude analytical HPLC of the reaction of model **7** with trap **2;** analytical gradient 2-40% B over 10 min, 280 nm. Peak corresponding to product **11** identified.

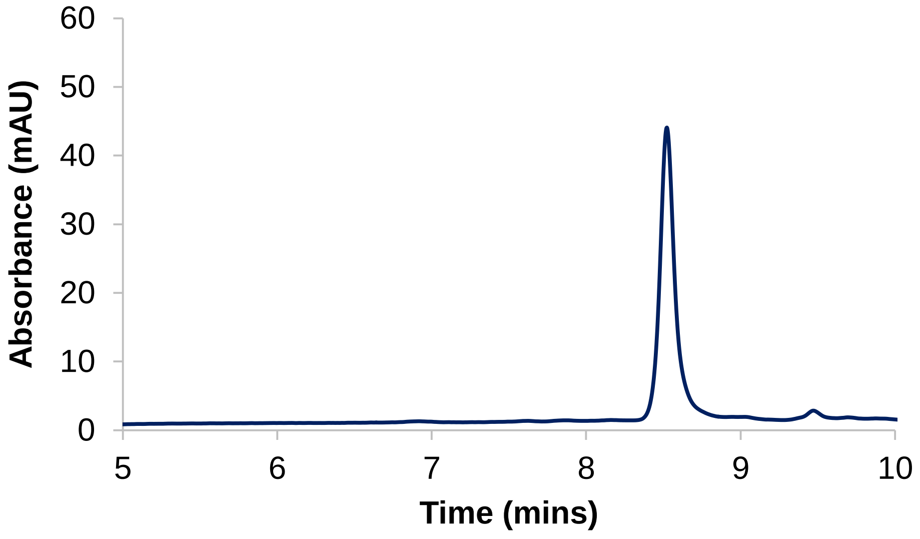

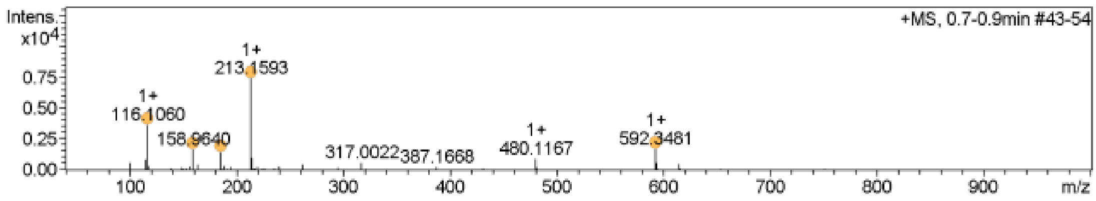

Figure S23 – Analytical HPLC trace of product **11**; analytical gradient 2-25% B over 10 min, 280 nm. Calculated Mass $[M+H]^+$: 592.33; Observed Mass $[M+H]^+$: 592.34.

Modification of Ac-ECAY-NH2 (8) with *N*-allyl acetamide (2)

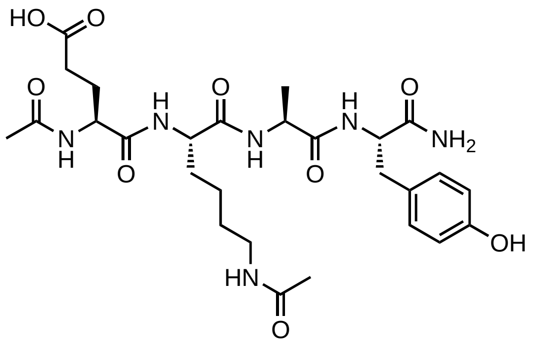

Peptide **12** was synthesized following the general conjugation protocol A (set up 2) using Ac-ECAY-NH2 (**8**, 3 mg, 3.81 µmol) and *N*-allyl acetamide (**2**, 75 mg, 0.76 mmol). The % conversion to the desired product **12** was calculated from the analytical HPLC of the crude reaction mixture. After analysis the remainder of material (3.76 µmol) was purified using purified using semi-preparative HPLC (5-60% B over 30 minutes) to yield the desired conjugated peptide (**12**, 0.7 mg, 1.18 µmol, 31% yield).

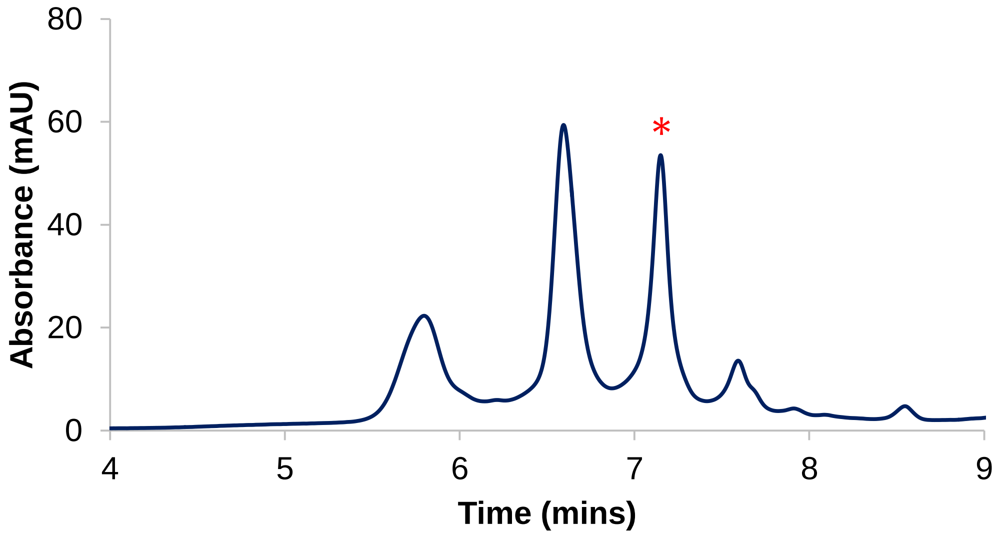

Figure S24 – Crude analytical HPLC of the reaction of model **8** with trap **2;** analytical gradient 2-40% B over 10 min, 280 nm. Peak corresponding to product **12** identified.

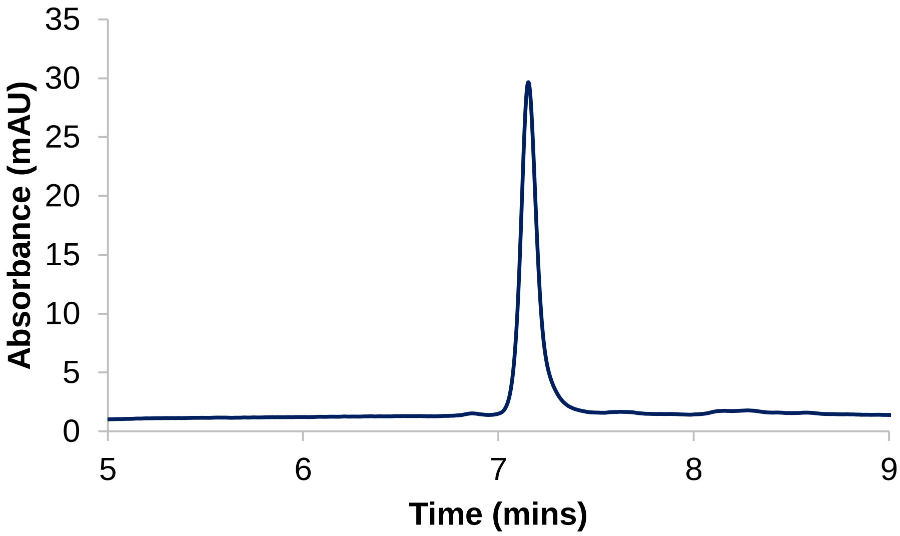

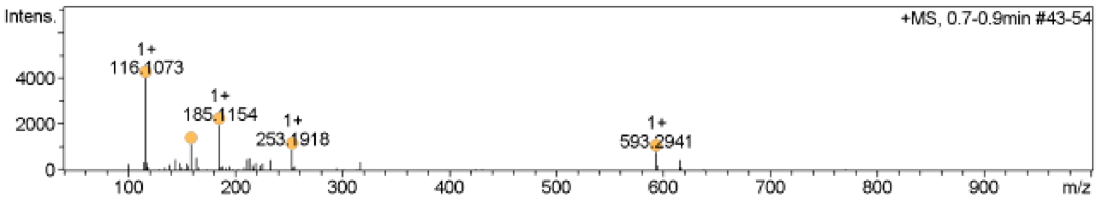

Figure S25 – Analytical HPLC trace of product **12**; analytical gradient 2-40% B over 10 min, 280 nm. Calculated Mass $[M+H]^+$: 593.29; Observed Mass $[M+H]^+$: 593.29.

Modification of Ac-YCAY-NH2 (9) with *N*-allyl acetamide (2)

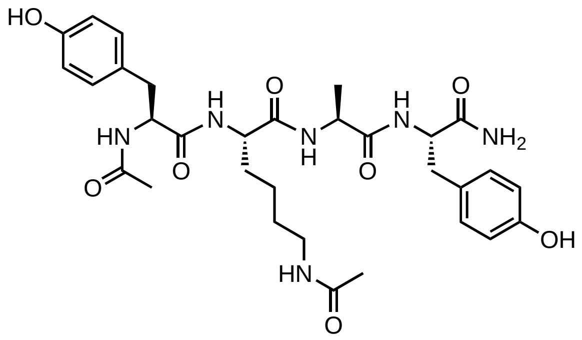

Peptide **13** was synthesized following the general conjugation protocol A (set up 2) using Ac-YCAY-NH2 (**9**, 2 mg, 3.58 µmol) and *N*-allyl acetamide (**2**, 70 mg, 0.71 mmol). The % conversion to the desired product **13** was calculated from the analytical HPLC of the crude reaction mixture. After analysis the remainder of material (3.53 µmol) was purified using purified using semi-preparative HPLC (5-60% B over 30 minutes) to yield the desired conjugated peptide (**13**, 1.3 mg, 2.08 µmol, 59% yield).

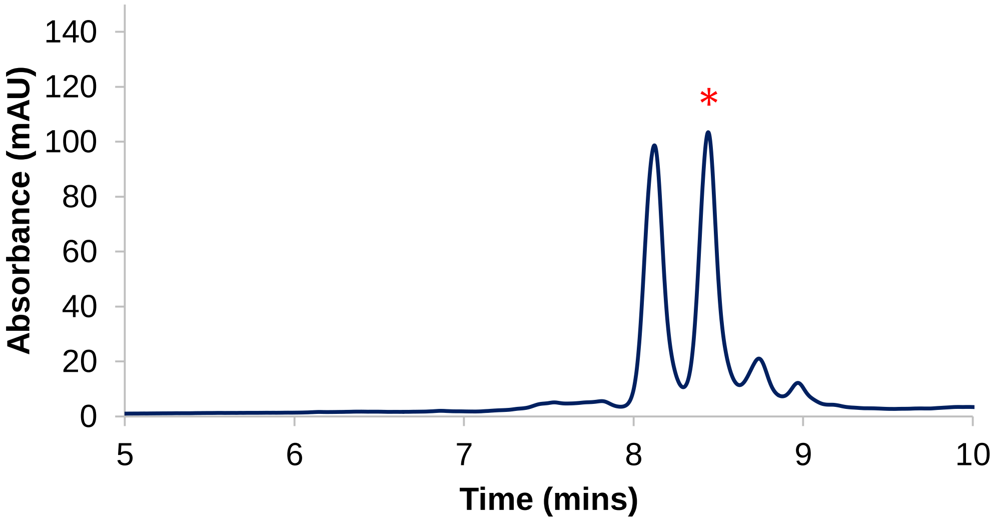

Figure S26 – Crude analytical HPLC of the reaction of model **9** with trap **2;** analytical gradient 2-40% B over 10 min, 280 nm. Peak corresponding to product **13** identified.

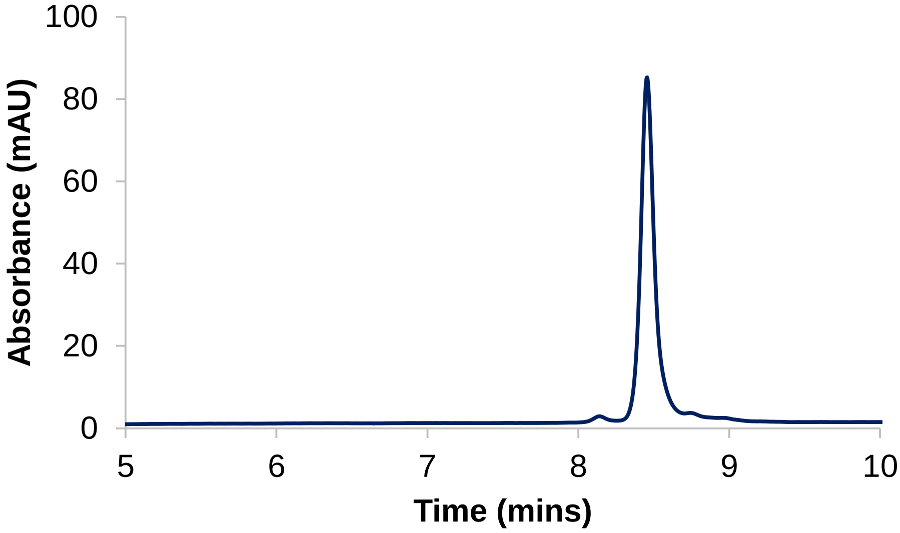

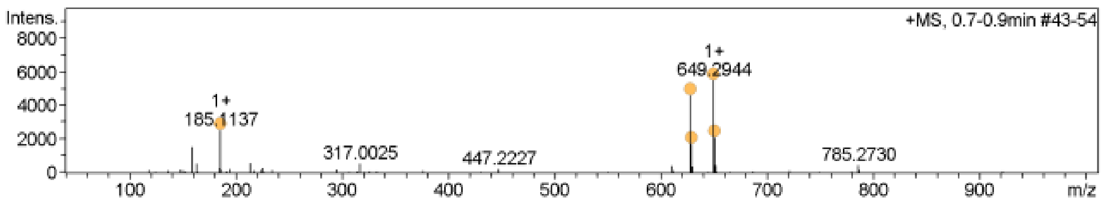

Figure S27 Analytical HPLC trace product **13**; analytical gradient 2-40% B over 10 min, 280 nm. Calculated Mass [M+Na]$^{+}$: 649.30; Observed Mass [M+H]$^{+}$: 649.29.

Modification of Ac-ICAY-NH2 (10) with *N*-allyl acetamide (2)

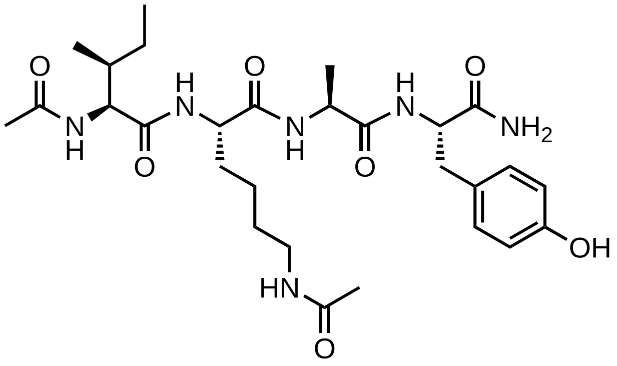

Peptide **14** was synthesized following the general conjugation protocol A (set up 2) using Ac-ICAY-NH2 (**10**, 3 mg, 3.81 µmol) and *N*-allyl acetamide (**2**, 75 mg, 0.76 mmol). The % conversion to the desired product **14** was calculated from the analytical HPLC of the crude reaction mixture. After analysis the remainder of material (3.88 µmol) was purified using purified using semi-preparative HPLC (5-60% B over 30 minutes) to yield the desired conjugated peptide (**14**, 1.2 mg, 2.08 µmol, 54% yield).

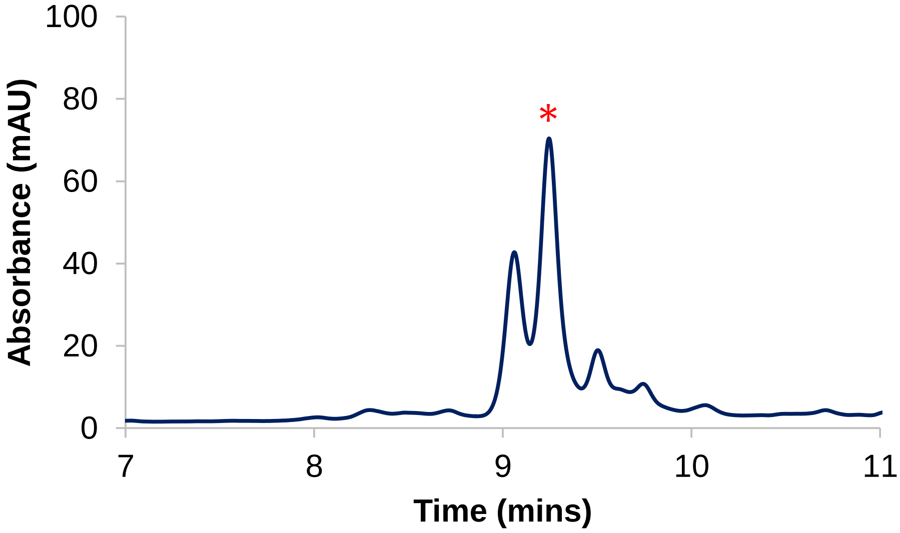

Figure S28 – Crude analytical HPLC of the reaction of model **10** with trap **2;** analytical gradient 2-40% B over 10 min, 280 nm. Peak corresponding to product **14** identified.

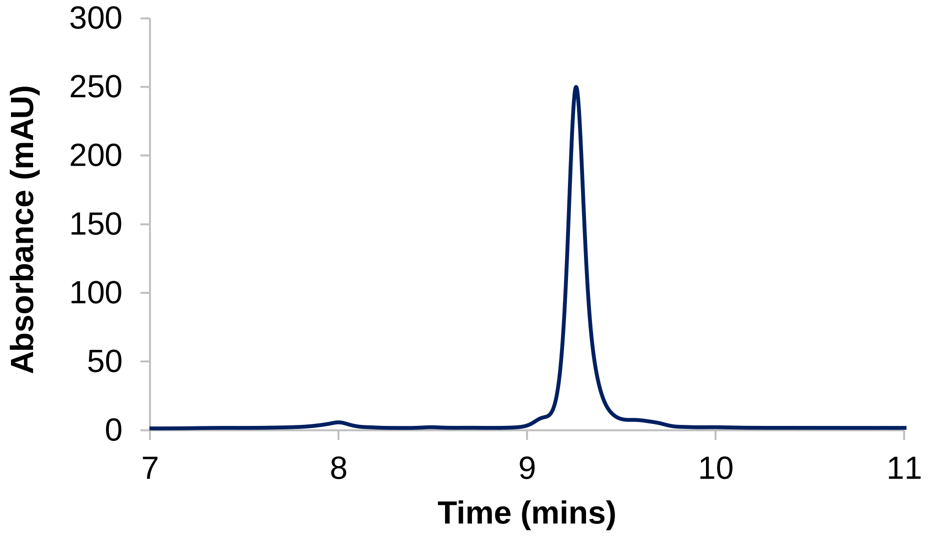

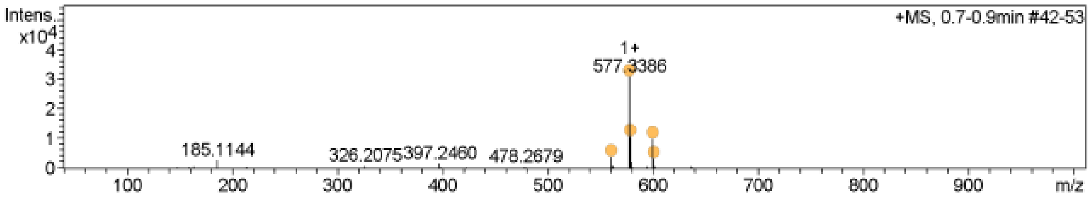

Figure S29 – Analytical HPLC trace of product **14**; analytical gradient 2-40% B over 10 min, 280 nm. Calculated Mass $[M+H]^+$: 577.33; Observed Mass $[M+H]^+$: 577.34.

Installation of native N^{ε}-modifications into a histone model peptide

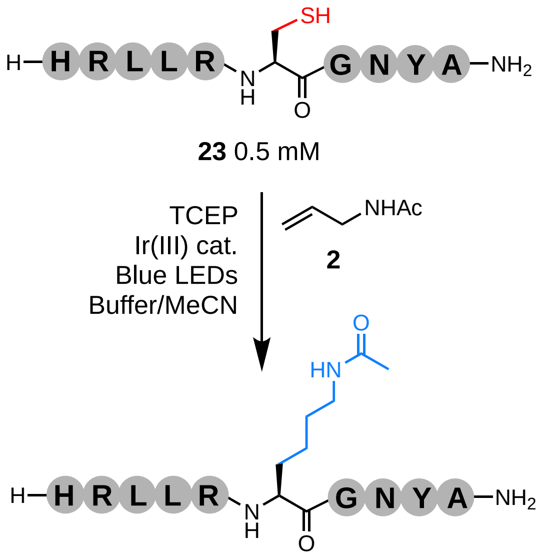

23 0.5 mM

24

Table 8 - Initial analytical scale reactions on histone model using 0.5 mM peptide and 200 eq. N-allyl acetamide in 20% DMSO in LB at pH 8. Values given are percentage conversion to desired to product. Value in brackets is the remaining starting material. The amount not accounted for is either Ala or multiple addition by-products.

Trial	TCEP eq.	Ir eq.	1 hour % conv.	2 hours % conv.	3 hours % conv.
53	5	0.05	33 (54)	46 (30)	50 (6)
54	10	0.05	47 (33)	56 (13)	61 (0)
55	5	0.1	26 (63)	40 (39)	48 (0)
56	10	0.1	47 (28)	62 (0)	63 (0)

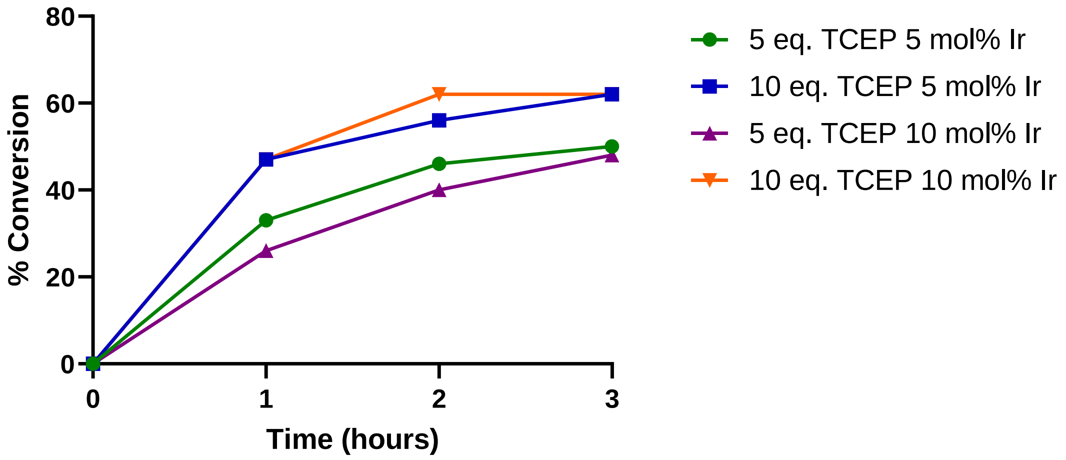

Table 9 - Organic co-solvent compatibility was tested to offer alternatives to DMSO. The reactions were performed on the histone model at 0.5 mM with 10 eq. TCEP, 200 eq. N-allyl acetamide, 10 mol% Ir(III) cat. in LB at pH 8 with the specified organic solvent. Reactions were monitored after 2 hours.

Trial	Solvent	Product %conv.	SM % rem.
57	20% Methanol	63	0
58	20% Acetonitrile	63	0

Based on these results, the conditions from trial 58 were taken forward for preparative scale reactions on this model, using % as the organic co-solvent using photochemistry set up 1.

Repeating these conditions in the PhotoRedOx Box (set up 2), we observed comparable conversion to the desired product in 2 mins.

Substrate scope on histone model peptide

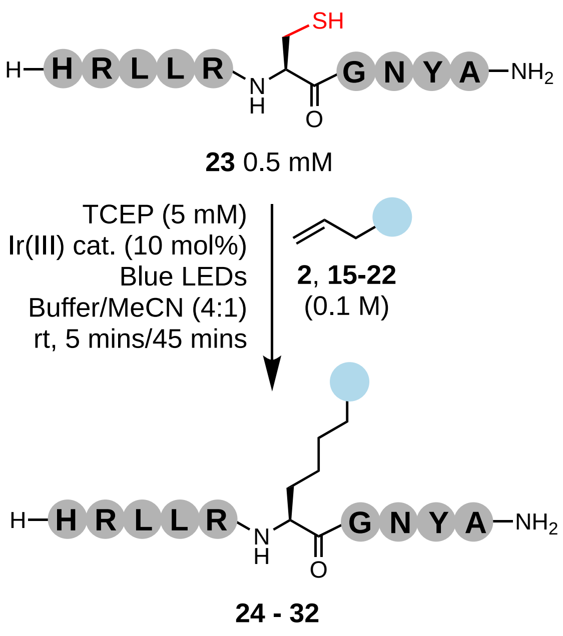

H-HRLLRCGNYA-NH2 (23) + *N*-allyl acetamide (2) – photochemistry set up 1

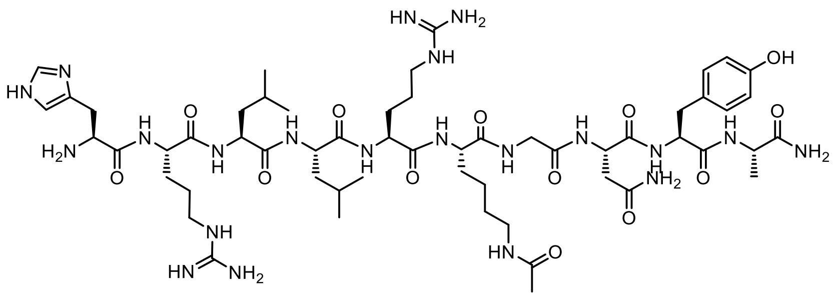

Peptide **24** was synthesized following the general conjugation protocol B (set up 1) using H-HRLLRCGNYA-NH2 (**23**) (2 mg, 1.66 µmol) and *N*-allyl acetamide (**2**, 33 mg, 0.33 mmol). After analysis the remainder of material (1.565 µmol) was purified using semi-preparative HPLC (5-40% B over 30 minutes) to yield the desired conjugated peptide (**24**, 1.2 mg, 0.946 µmol, 60% yield).

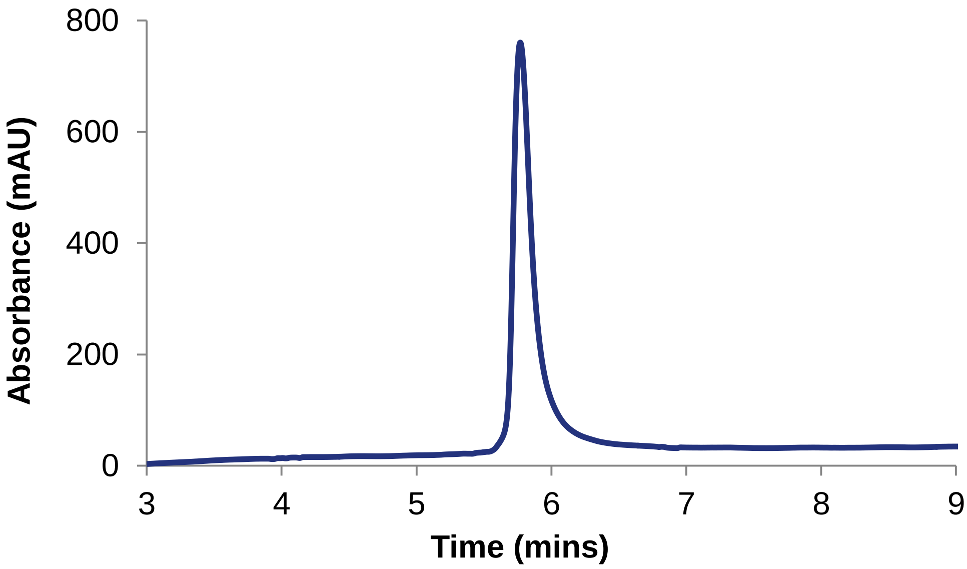

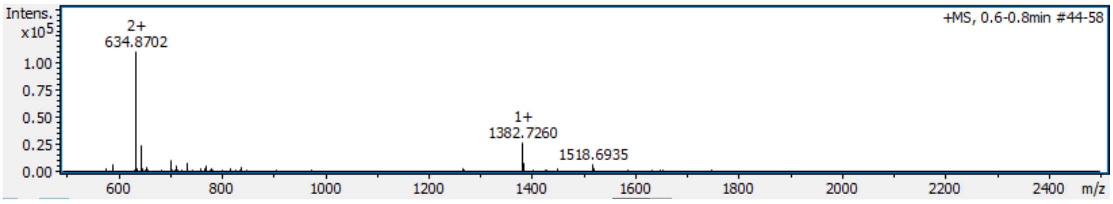

Figure S30 - Analytical HPLC trace of pure product **24**. Analytical gradient 10-30% B over 10 min, 210 nm. Calculated Mass [M+H+TFA]$^+$: 1382.73, [M+2H]$^{2+}$: 634.87. Observed Mass [M+H+TFA]$^+$: 1382.73, [M+2H]$^{2+}$: 634.87.

H-HRLLRCGNYA-NH2 (23) + *N*-allyl acetamide (2) – photochemistry set up 2

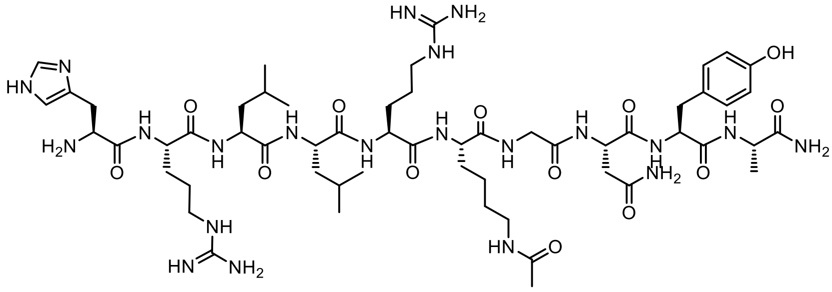

Peptide **24** was synthesized following the general conjugation protocol B (set up 2) using H-HRLLRCGNYA-NH2 (**23**, 2 mg, 1.66 µmol) and *N*-allyl acetamide (**2**, 33 mg, 0.33 mmol). After analysis the remainder of material (1.64 µmol) was purified using semi-preparative HPLC (5-40% B over 30 minutes) to yield the desired conjugated peptide (**24**, 1.4 mg, 1.10 µmol, 68% yield).

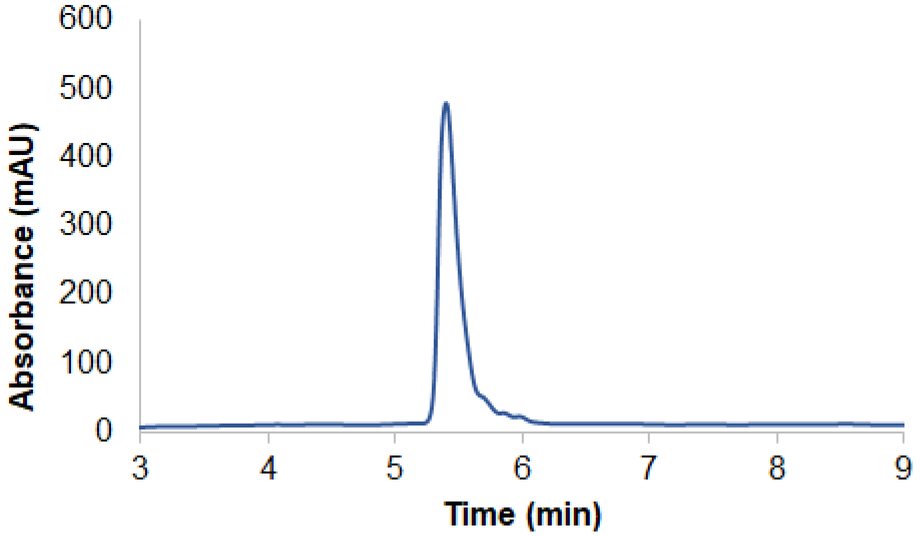

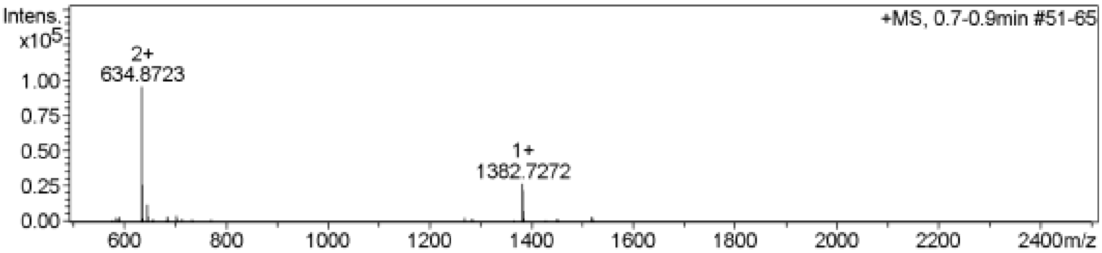

Figure S31 - Analytical HPLC trace of pure product **24**. Analytical gradient 10-30% B over 10 min, 210 nm. Calculated Mass $[M+H+TFA]^{+}$: 1382.73, $[M+2H]^{2+}$: 634.87. Observed Mass $[M+H+TFA]^{+}$: 1382.73, $[M+2H]^{2+}$: 634.87.

H-HRLLRCGNYA-NH2 (23) + *N*-methylallylamine (15) - photochemistry set up 1

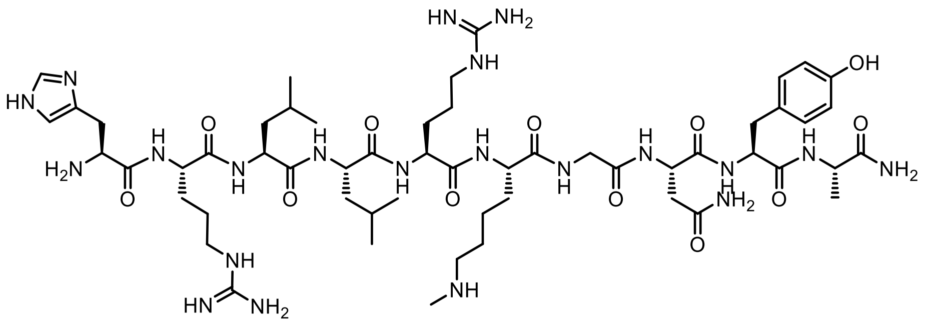

Peptide **25** was synthesised following the general conjugation protocol B (set up 1) using H-HRLLRCGNYA-NH2 (**23**, 4 mg, 3.32 µmol) and *N*-methyl allylamine (**15**, 47 mg, 0.66 mmol). After analysis the remainder of material (3.23 µmol) was purified using purified using semi-preparative HPLC (5-40% B over 30 minutes) to yield the desired conjugated peptide (**25**, 1.9 mg, 1.53 µmol, 47% yield).

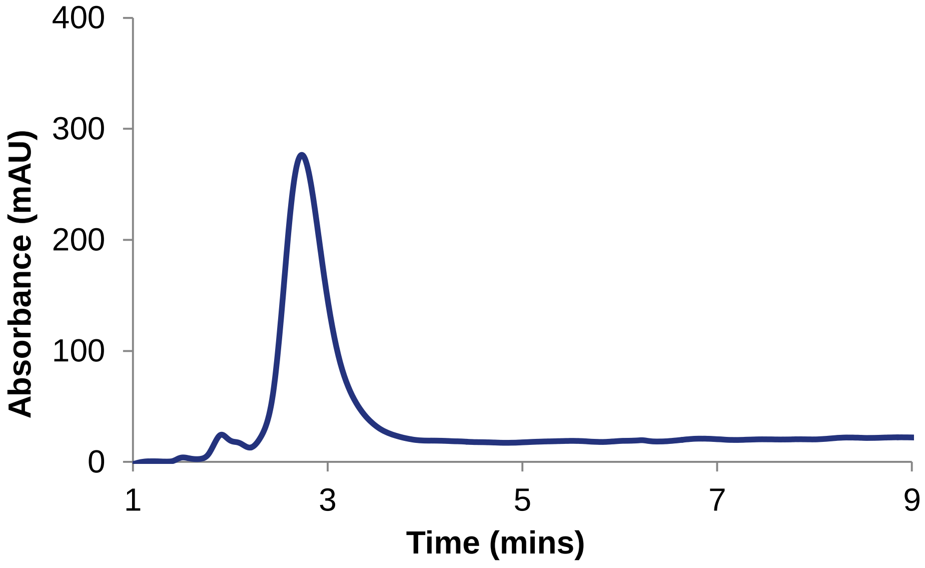

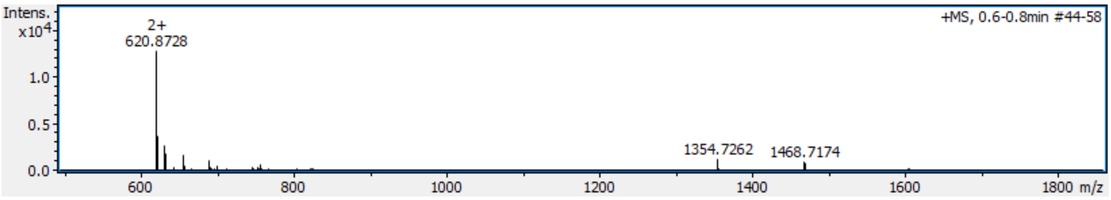

Figure S32 - Analytical HPLC trace of pure product **25**. Analytical gradient 10-30% B over 10 min, 210 nm. Calculated Mass [M+H+TFA]$^+$: 1354.73, [M+2H]$^{2+}$: 620.87. Observed Mass [M+H+TFA]$^+$: 1354.73, [M+2H]$^{2+}$: 620.87.

H-HRLLRCGNYA-NH2 (23) + *N*-methylallylamine (15) – photochemistry set up 2

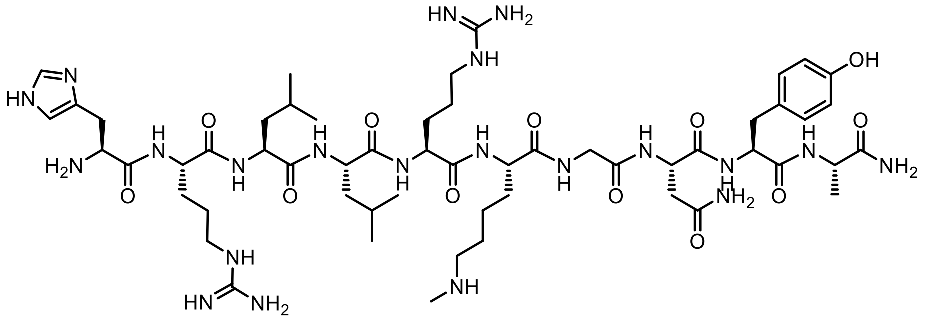

Peptide **25** was synthesised following the general conjugation protocol B (set up 2) using H-HRLLRCGNYA-NH2 (**23**, 2 mg, 1.66 µmol) and N-methyl allylamine (**15**, 24 mg, 0.33 mmol). After analysis the remainder of material (1.64 µmol) was purified using purified using semi-preparative HPLC (5-40% B over 30 minutes) to yield the desired conjugated peptide (**25**, 1.1 mg, 0.89 µmol, 54% yield).

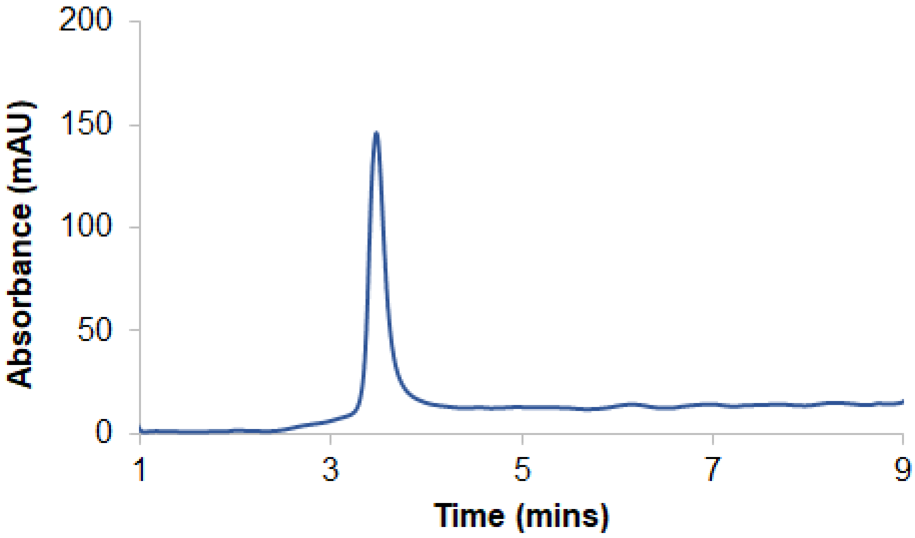

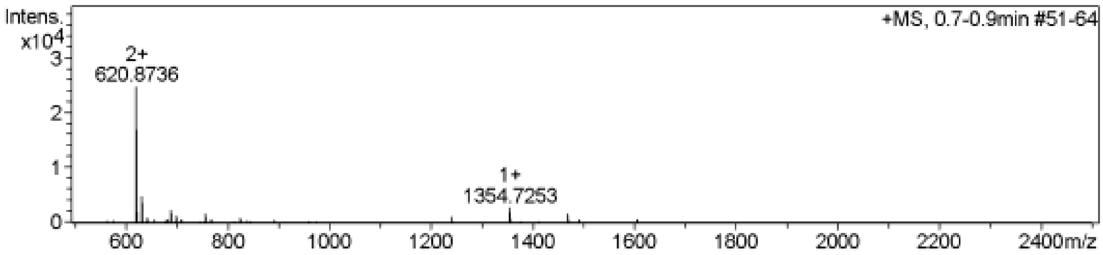

Figure S33 - Analytical HPLC trace of pure product **25**. Analytical gradient 10-30% B over 10 min, 210 nm. Calculated Mass [M+H+TFA]$^{+}$: 1354.73, [M+2H]$^{2+}$: 620.87. Observed Mass [M+H+TFA]$^{+}$: 1354.73, [M+2H]$^{2+}$: 620.87.

H-HRLLRCGNYA-NH2 (23) + *N,N*-dimethylallylamine (16) – photochemistry set up 1

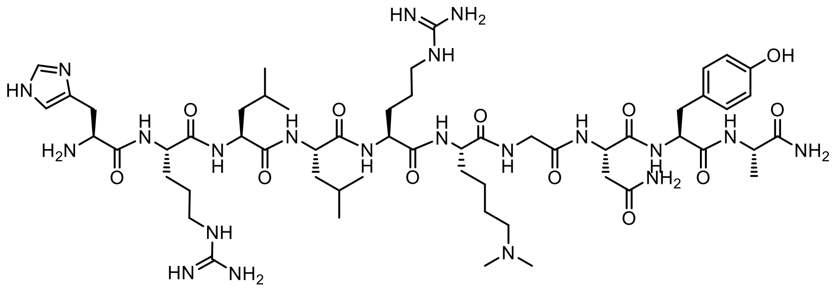

Peptide **26** was synthesised following the general conjugation protocol B (set up 1) using H-HRLLRCGNYA-NH2 (**23**, 2 mg, 1.66 µmol) and *N,N*-dimethyl allylamine (**16**, 28 mg, 0.33 mmol). After analysis the remainder of material (1.565 µmol) was purified using semi-preparative HPLC (5-40% B over 30 minutes) to yield the desired conjugated peptide (**26**, 1.1 mg, 0.877 µmol, 56% yield).

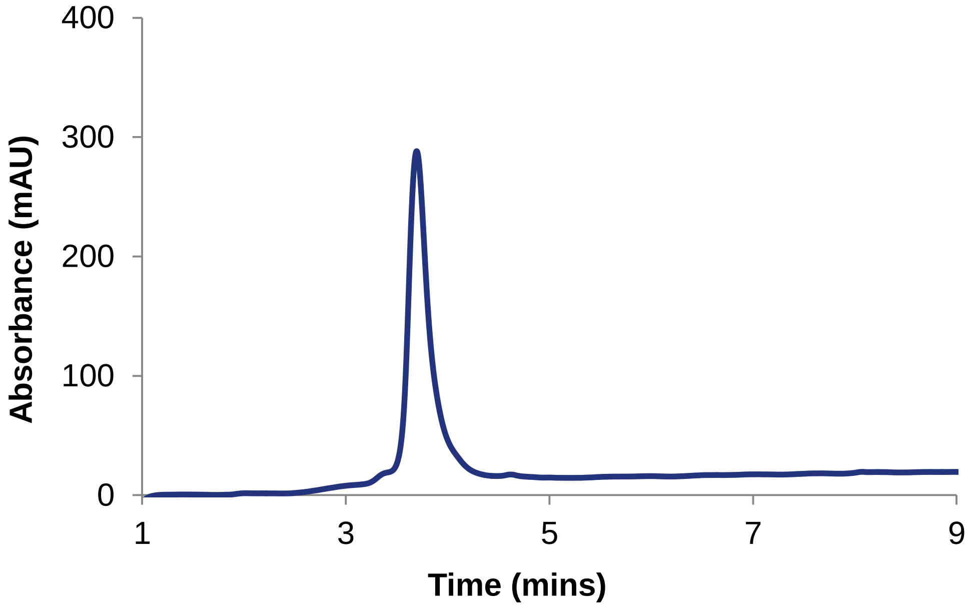

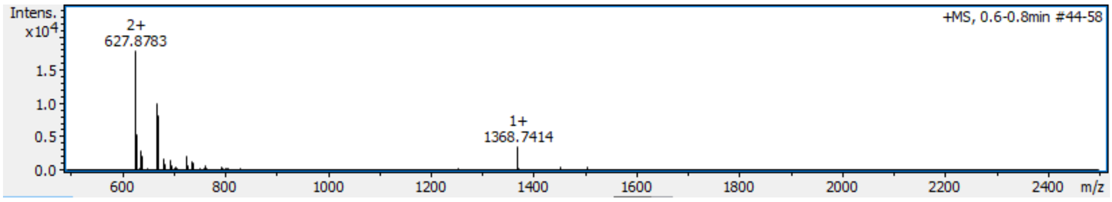

Figure S34 - Analytical HPLC trace of pure product **26**. Analytical gradient 10-30% B over 10 min, 210 nm. Calculated Mass $[M+H+TFA]^{+}$: 1368.75, $[M+2H]^{2+}$: 627.88. Observed Mass $[M+H+TFA]^{+}$: 1368.74, $[M+2H]^{2+}$: 627.88.

H-HRLLRCGNYA-NH2 (23) + *N,N*-dimethyl allylamine (16) – photochemistry set up 2

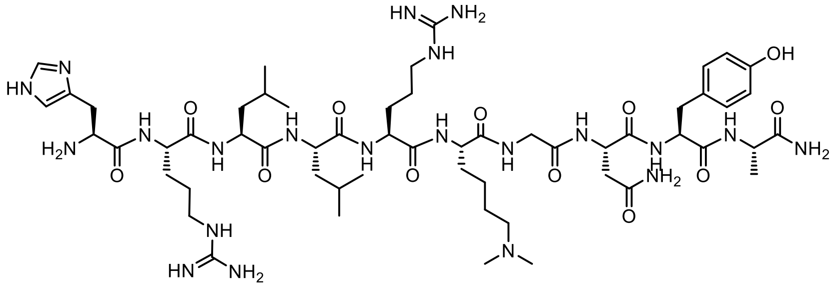

Peptide **26** was synthesised following the general conjugation protocol B (set up 2) using H-HRLLRCGNYA-NH2 (**23**, 2 mg, 1.66 µmol) and *N,N*-dimethyl allylamine (**16**, 28 mg, 0.33 mmol). After analysis the remainder of material (1.565 µmol) was purified using semi-preparative HPLC (5-40% B over 30 minutes) to yield the desired conjugated peptide (**26**, 1.17 mg, 0.933 µmol, 59% yield).

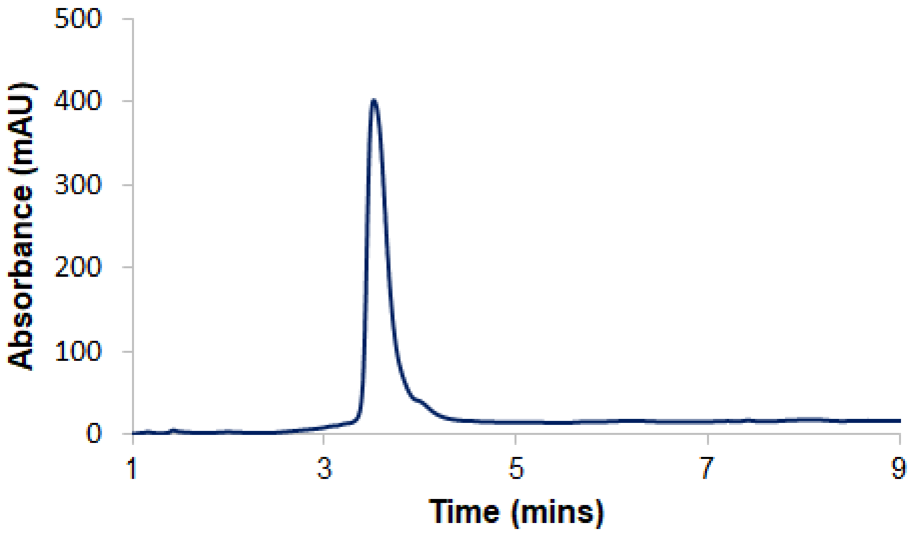

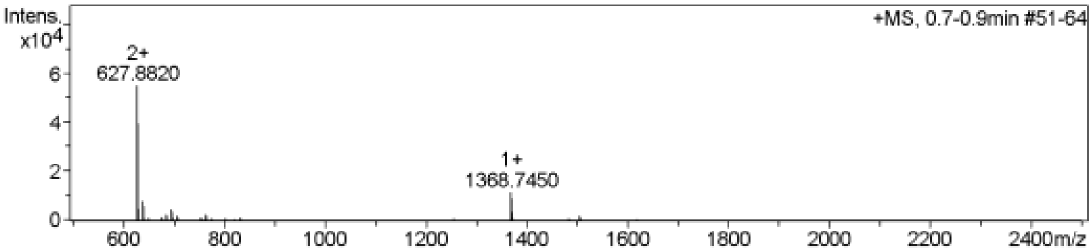

Figure S35 - Analytical HPLC trace of pure product **26**. Analytical gradient 10-30% B over 10 min, 210 nm. Calculated Mass $[M+H+TFA]^+$: 1368.75, $[M+2H]^{2+}$: 627.88. Observed Mass $[M+H+TFA]^+$: 1368.75, $[M+2H]^{2+}$: 627.88.

H-HRLLRCGNYA-NH2 (23) + allyltrimethylammonium chloride (17) – photochemistry set up 1

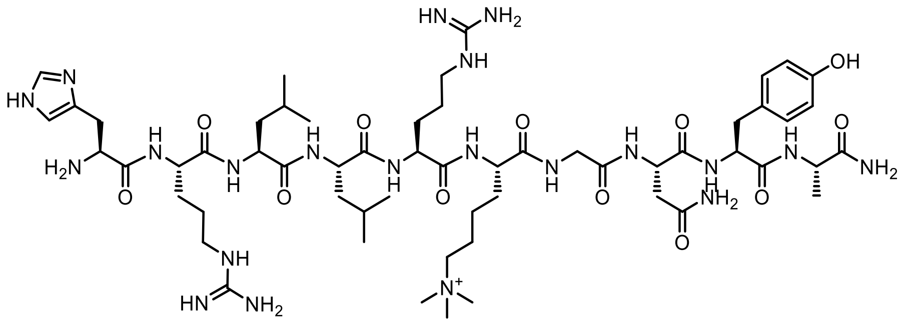

Peptide **27** was synthesised following the general conjugation protocol B (set up 1) using H-HRLLRCGNYA-NH2 (**23**, 2 mg, 1.66 µmol) and allyltrimethylammonium chloride (**17**, 45 mg, 0.33 mmol). After analysis the remainder of material (1.565 µmol) was purified using purified using semi-preparative HPLC (5-40% B over 30 minutes) to yield the desired conjugated peptide (**27**, 1.2 mg, 0.920 µmol, 59% yield).

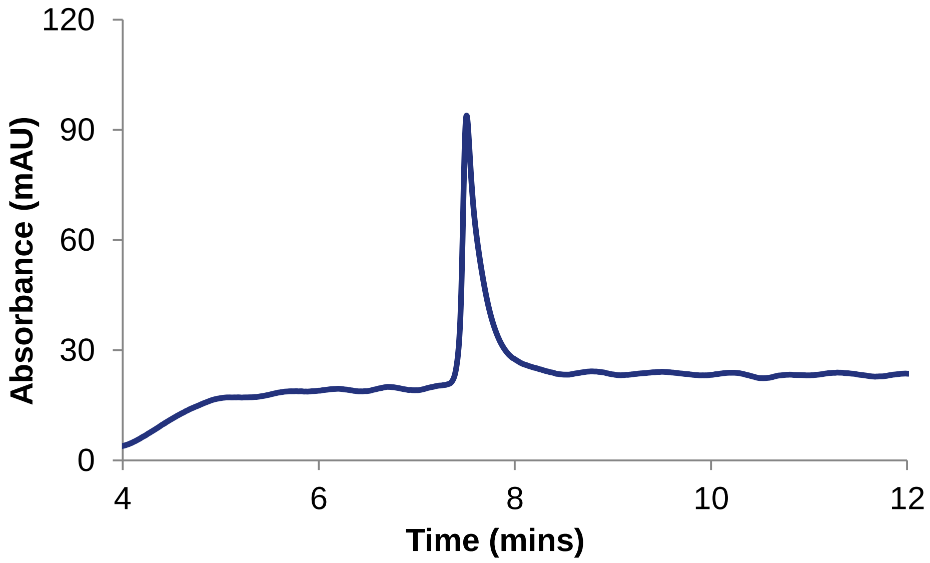

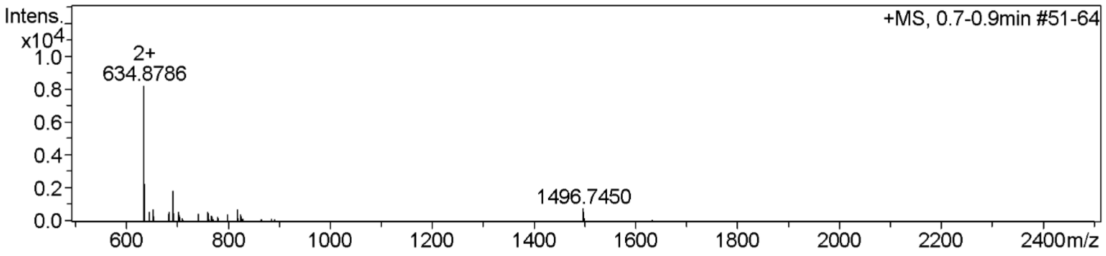

Figure S36 - Analytical HPLC trace of pure product **27**. Analytical gradient 5-30% B over 10 min, 210 nm. Calculated Mass $[M+H]^{2+}$: 634.89. Observed Mass $[M+H]^{2+}$: 634.88.

H-HRLLRCGNYA-NH2 (23) + allyltrimethylammonium chloride (17) – photochemistry set up 2

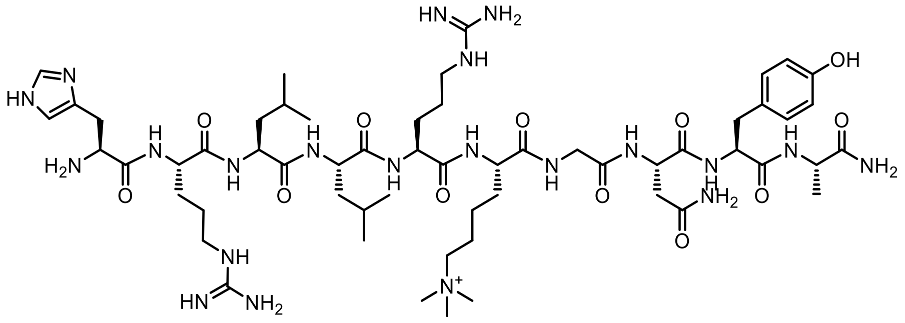

Peptide **27** was synthesised following the general conjugation protocol B (set up 2) using H-HRLLRCGNYA-NH2 (**23**, 2 mg, 1.66 μmol) and allyltrimethylammonium chloride (**17**, 45 mg, 0.33 mmol). After analysis the remainder of material (1.565 μmol) was purified using purified using semi-preparative HPLC (5-40% B over 30 minutes) to yield the desired conjugated peptide (**27**, 1.13 mg, 0.890 μmol, 54% yield).

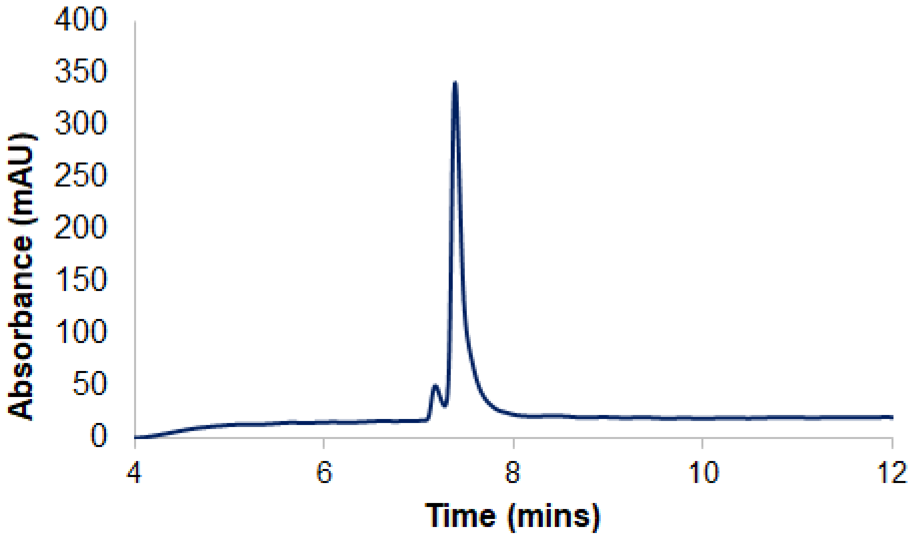

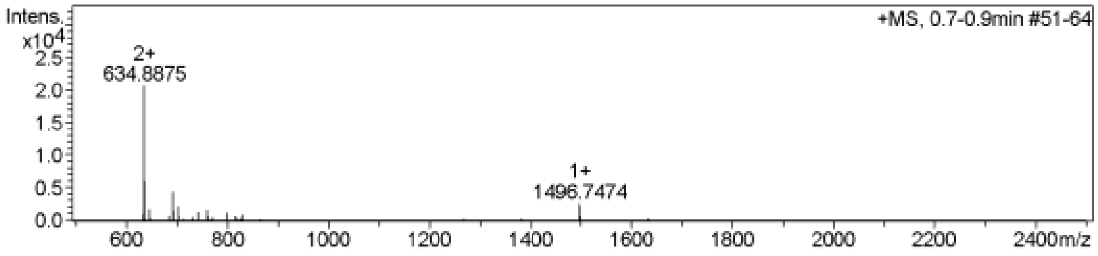

Figure S37 - Analytical HPLC trace of pure product **27**. Analytical gradient 5-30% B over 10 min, 210 nm. Calculated Mass $[M+H]^{2+}$: 634.89. Observed Mass $[M+H]^{2+}$: 634.89.

H-HRLLRCGNYA-NH2 (23) + *N*-allyl formamide (18) – photochemistry set up 1

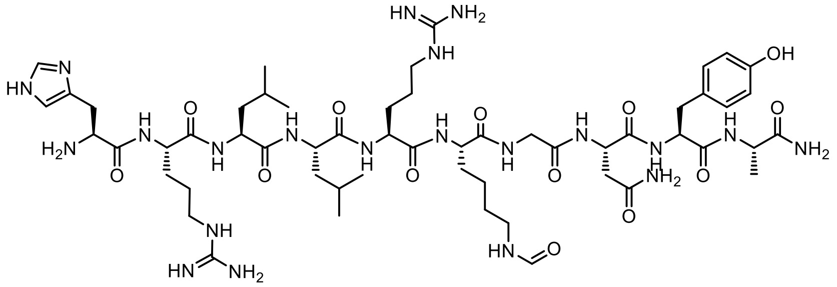

Peptide **28** was synthesised following the general conjugation protocol B (set up 1) using H-HRLLRCGNYA-NH2 (**23**, 2 mg, 1.66 µmol) and *N*-allyl formamide (**18**, 28 mg, 0.33 mmol). After analysis the remainder of material (1.565 µmol) was purified using purified using semi-preparative HPLC (5-40% B over 30 minutes) to yield the desired conjugated peptide (**28**, 1.1 mg, 0.877 µmol, 56% yield).

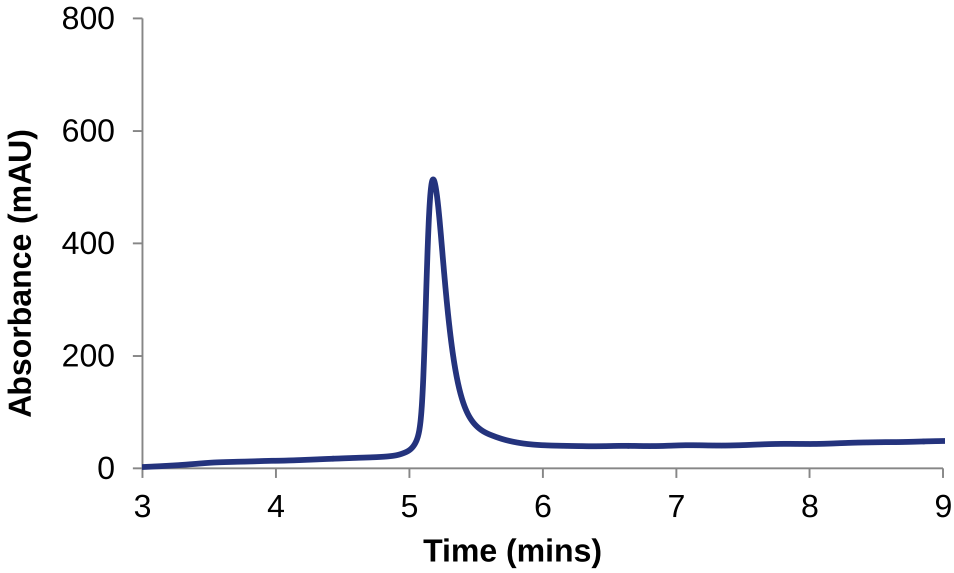

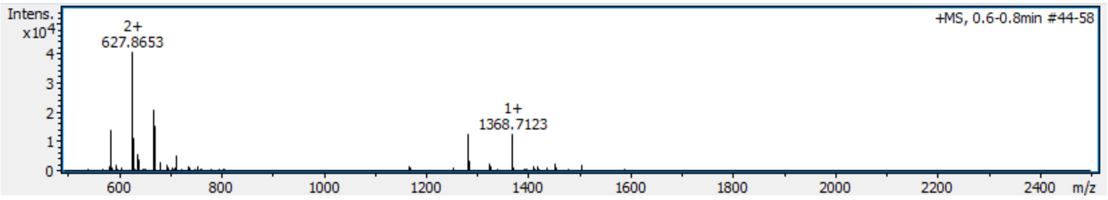

Figure S38 - Analytical HPLC trace of pure product **28**. Analytical gradient 10-30% B over 10 min, 210 nm. Calculated Mass $[M+H+TFA]^{+}$: 1368.71, $[M+2H]^{2+}$: 627.86. Observed Mass $[M+H+TFA]^{+}$: 1368.71, $[M+2H]^{2+}$: 627.87.

H-HRLLRCGNYA-NH2 (23) + *N*-allyl formamide (18) – photochemistry set up 2

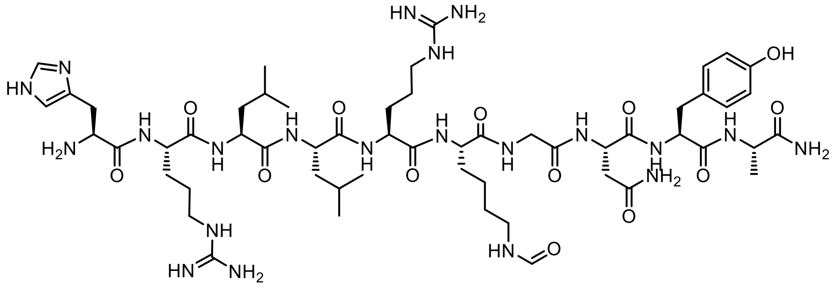

Peptide **28** was synthesised following the general conjugation protocol B (set up 2) using H-HRLLRCGNYA-NH2 (**23**, 2 mg, 1.66 µmol) and *N*-allyl formamide (**18**, 28 mg, 0.33 mmol). After analysis the remainder of material (1.64 µmol) was purified using purified using semi-preparative HPLC (5-40% B over 30 minutes) to yield the desired conjugated peptide (**28**, 1.24 mg, 0.988 µmol, 60% yield).

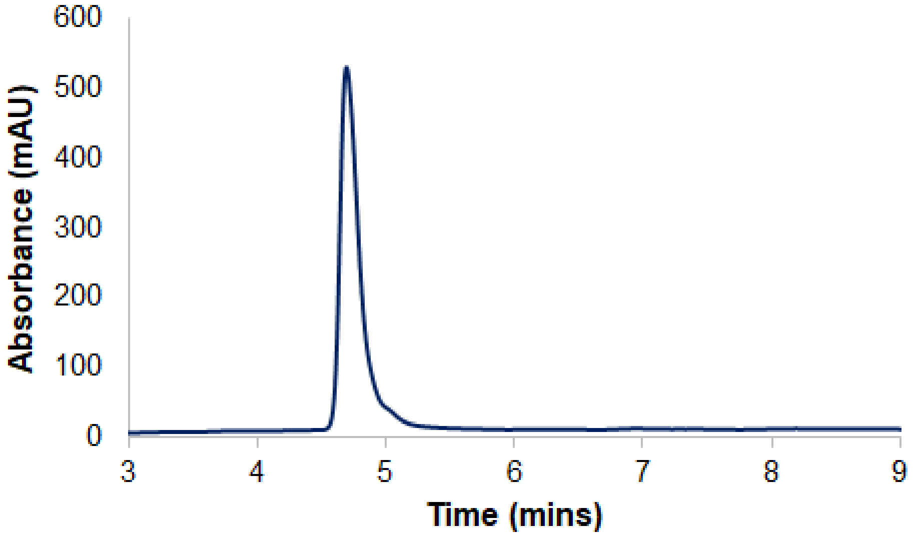

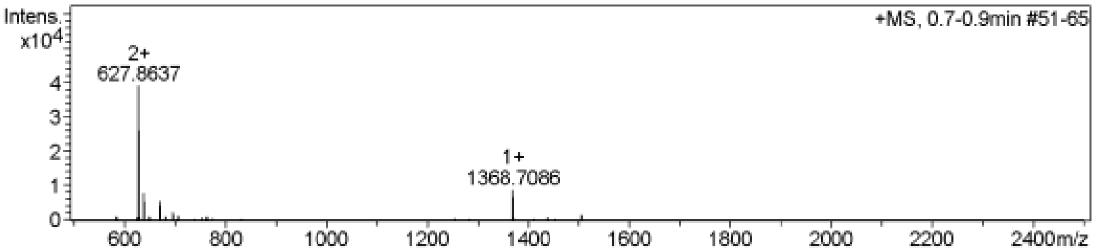

Figure S39 - Analytical HPLC trace of pure product **28**. Analytical gradient 10-30% B over 10 min, 210 nm. Calculated Mass [M+H+TFA]$^{+}$: 1368.71, [M+2H]$^{2+}$: 627.86. Observed Mass [M+H+TFA]$^{+}$: 1368.71, [M+2H]$^{2+}$: 627.86.

H-HRLLRCGNYA-NH2 (23) + *N*-allylpropionamide (19) – photochemistry set up 1

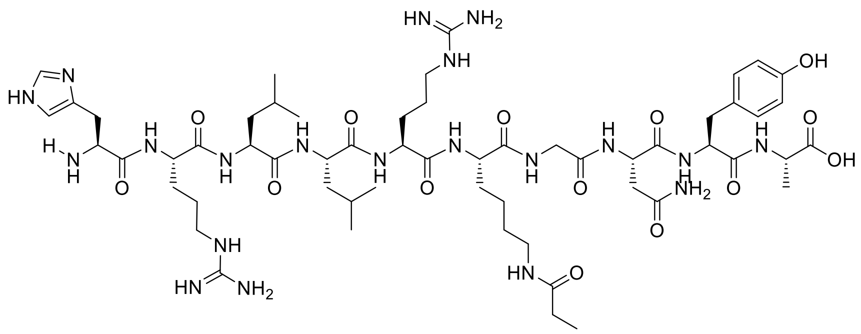

Peptide **29** was synthesised following the general conjugation protocol B (set up 1) using H-HRLLRCGNYA-NH2 (**23** 2 mg, 1.66 µmol) and *N*-allyl propionamide (**19**, 37 mg, 0.33 mmol). After analysis the remainder of material (1.64 µmol) was purified using purified using semi-preparative HPLC (5-40% B over 30 minutes) to yield the desired conjugated peptide (**29**, 1.2 mg, 0.94 µmol, 57% yield).

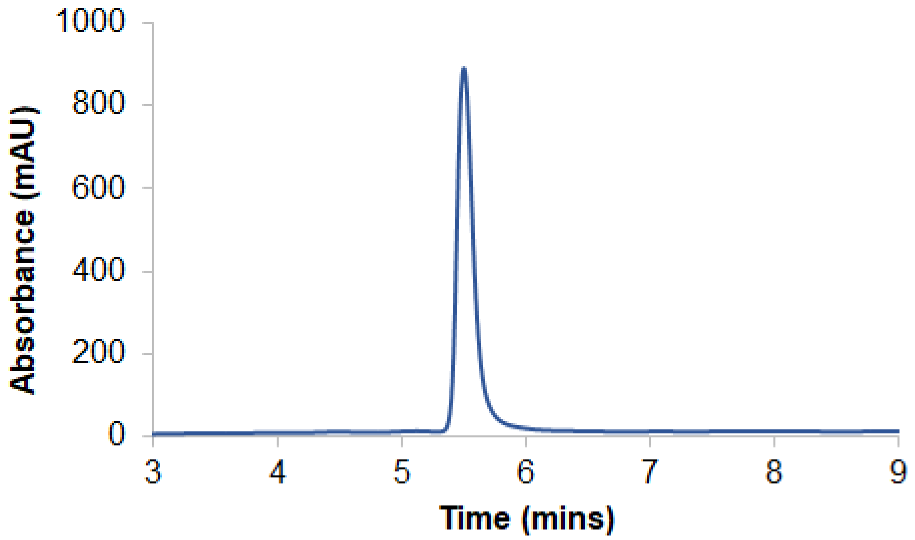

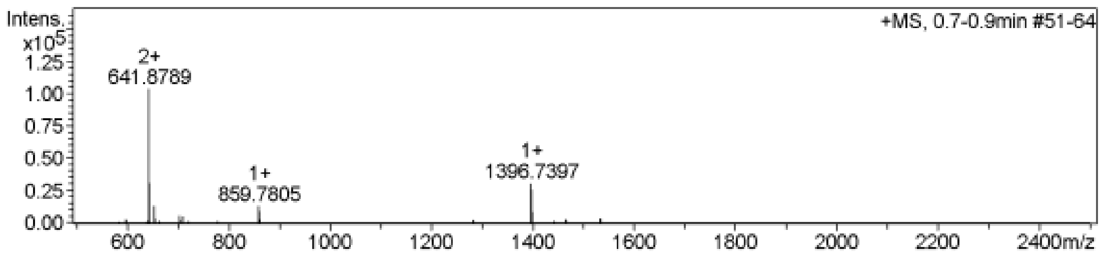

Figure S40 - Analytical HPLC trace of pure product **29**. Analytical gradient 10-30% B over 10 min, 210 nm. Calculated Mass [M+H+TFA]$^{+}$: 1396.71, [M+2H]$^{2+}$: 641.87. Observed Mass [M+H+TFA]$^{+}$: 1396.74, [M+2H]$^{2+}$: 641.88.

H-HRLLRCGNYA-NH2 (23) + *N*-allylpropionamide (19) – photochemistry set up 2

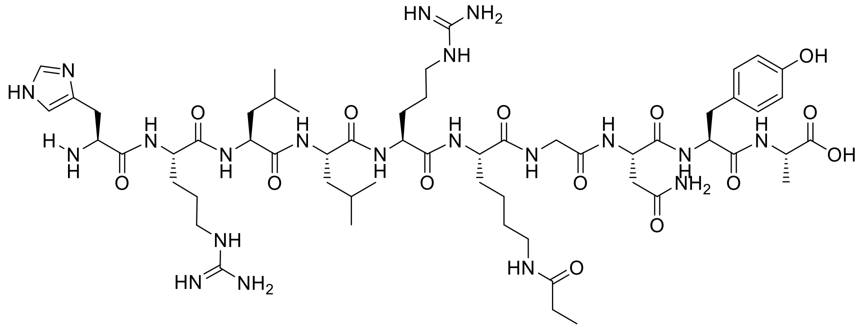

Peptide **29** was synthesised following the general conjugation protocol B (set up 2) for 30 min using H-HRLLRCGNYA-NH2 (**23** 2 mg, 1.66 µmol) and *N*-allyl propionamide (**19**, 37 mg, 0.33 mmol). After analysis the remainder of material (1.64 µmol) was purified using purified using semi-preparative HPLC (5-40% B over 30 minutes) to yield the desired conjugated peptide (**29**, 1.1 mg, 0.858 µmol, 52% yield).

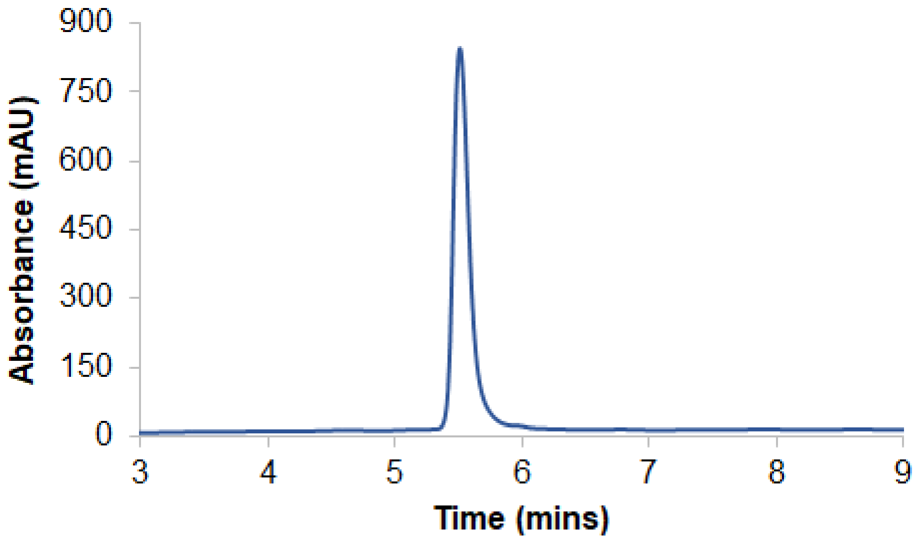

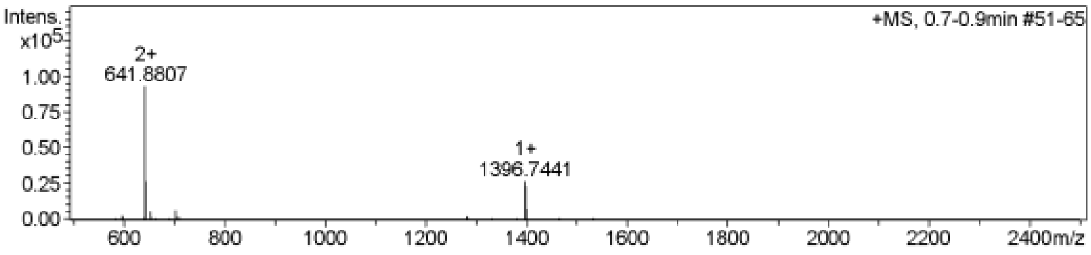

Figure S41 - Analytical HPLC trace of pure product **29**. Analytical gradient 10-30% B over 10 min, 210 nm. Calculated Mass $[M+H+TFA]^{+}$: 1396.72, $[M+2H]^{2+}$: 641.87. Observed Mass $[M+H+TFA]^{+}$: 1396.74, $[M+2H]^{2+}$: 641.88.

H-HRLLRCGNYA-NH2 (23) + *N*-allyl-2-hydroxy-2-methylpropanamide (20) – photochemistry set up 1

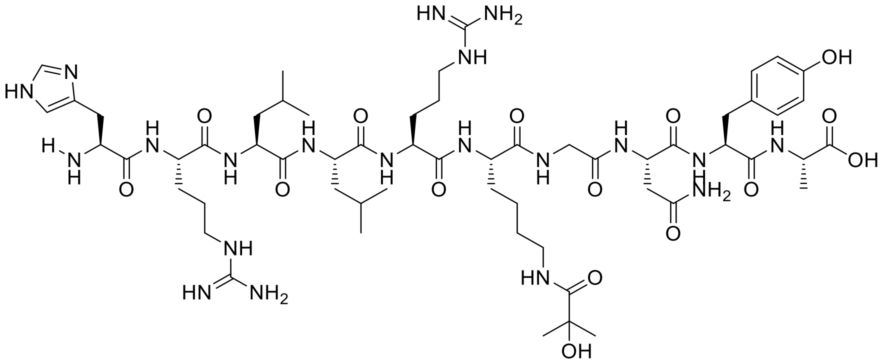

Peptide **30** was synthesised following the general conjugation protocol B (set up 1) using H-HRLLRCGNYA-NH2 (**23**, 2 mg, 1.66 µmol) and *N*-allyl-2-hydroxyl-2-methylpropanamide (**20**, 47 mg, 0.33 mmol). After analysis the remainder of material (1.565 µmol) was purified using purified using semi-preparative HPLC (5-40% B over 30 minutes) to yield the desired conjugated peptide (**30**, 1.1 mg, 0.833 µmol, 53% yield).

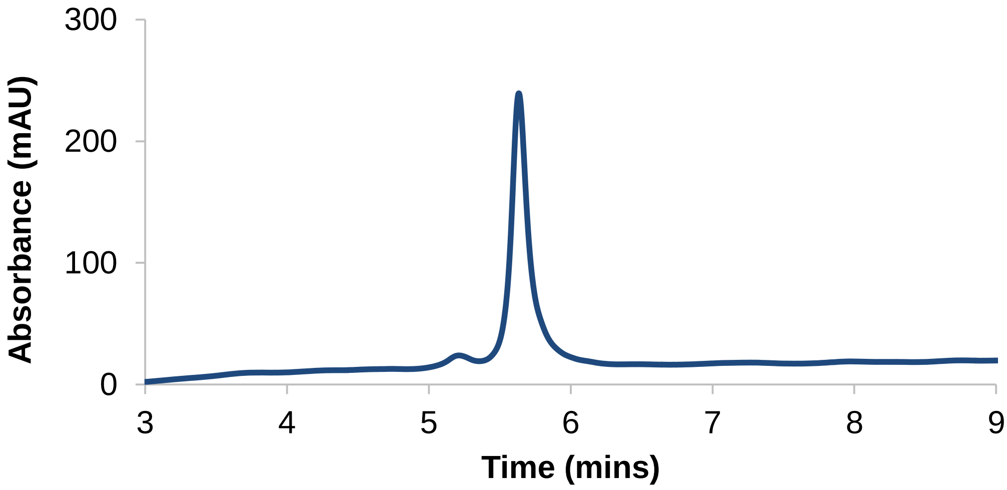

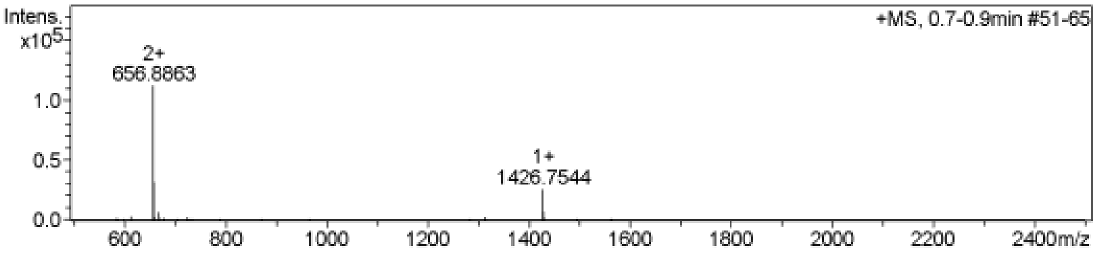

Figure S42 - Analytical HPLC trace of pure product **30**. Analytical gradient 10-30% B over 10 min, 210 nm. Calculated Mass $[M+H+TFA]^{+}$: 1426.73, $[M+2H]^{2+}$: 656.87. Observed Mass $[M+H+TFA]^{+}$: 1426.75, $[M+2H]^{2+}$: 656.89.

H-HRLLRCGNYA-NH2 (23) + *N*-allyl-2-hydroxy-2-methylpropanamide (20) – photochemistry set up 2

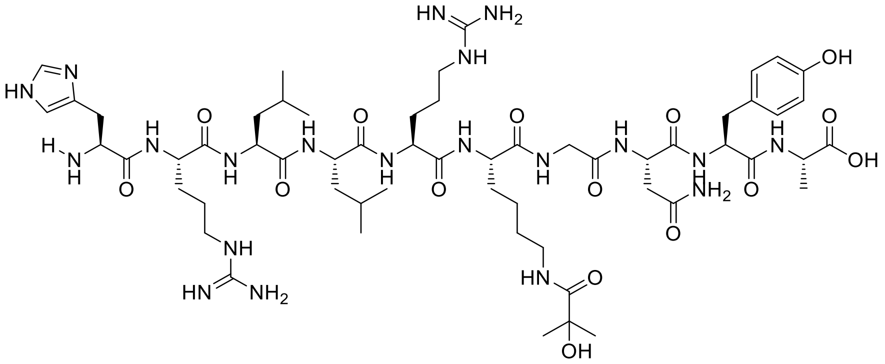

Peptide **30** was synthesised following the general conjugation protocol B (set up 2) using H-HRLLRCGNYA-NH2 (**23**, 2 mg, 1.66 µmol) and *N*-allyl-2-hydroxyl-2-methylpropanamide (**20**, 47 mg, 0.33 mmol). After analysis the remainder of material (1.565 µmol) was purified using purified using semi-preparative HPLC (5-40% B over 30 minutes) to yield the desired conjugated peptide (**30**, 1.3 mg, 0.833 µmol, 63% yield).

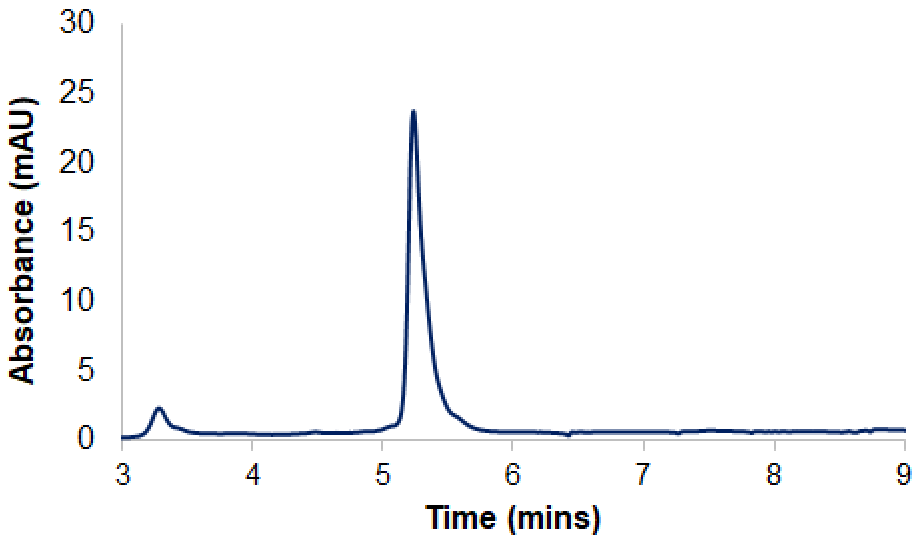

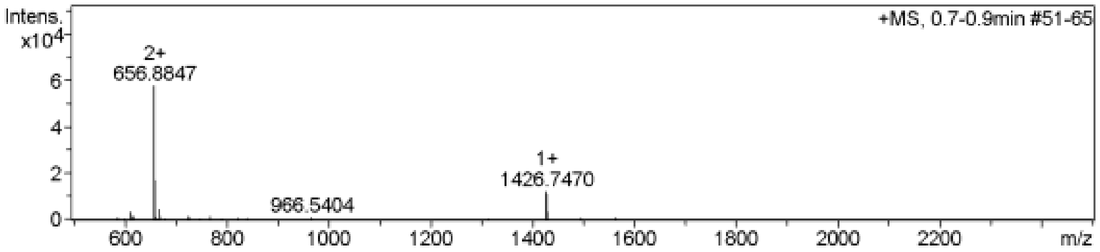

Figure S43 - Analytical HPLC trace of pure product **30**. Analytical gradient 10-30% B over 10 min, 210 nm. Calculated Mass $[M+H+TFA]^{+}$: 1426.73, $[M+2H]^{2+}$: 656.87. Observed Mass $[M+H+TFA]^{+}$: 1426.75, $[M+2H]^{2+}$: 656.88.

H-HRLLRCGNYA-NH2 (23) + 4-(allylamino)-4-oxobutanoic acid (21) – photochemistry set up 1

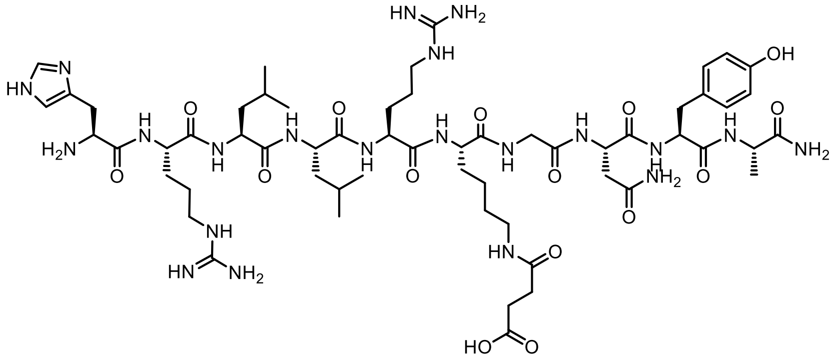

Peptide **31** was synthesised following the general conjugation protocol B (set up 1) using H-HRLLRCGNYA-NH2 (**23**, 2 mg, 1.66 µmol) and 4-(allylamin)-4-oxobutanoic acid (**21,** 52 mg, 0.33 mmol). After analysis the remainder of material (1.565 µmol) was purified using purified using semi-preparative HPLC (5-40% B over 30 minutes) to yield the desired conjugated peptide (**31**, 1.1 mg, 0.829 µmol, 53% yield).

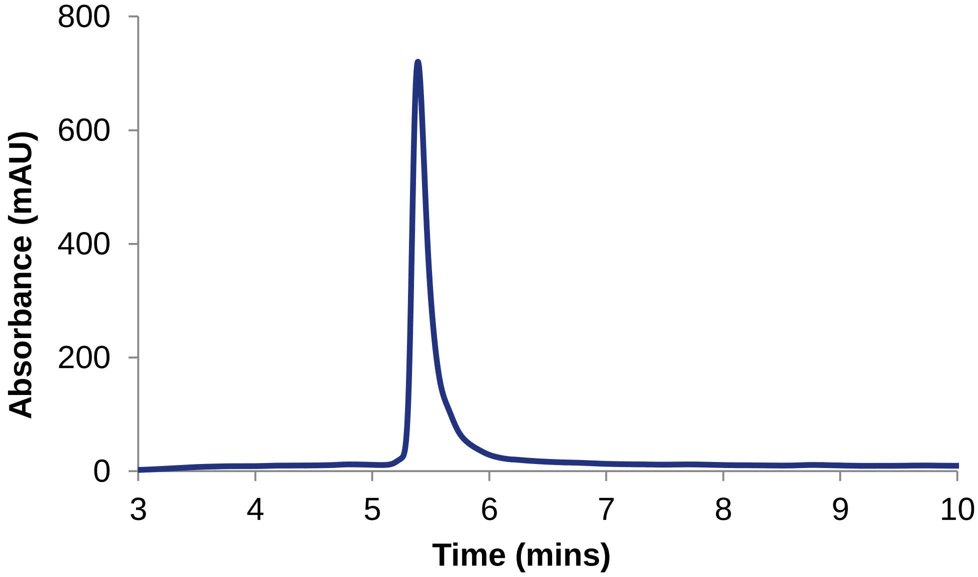

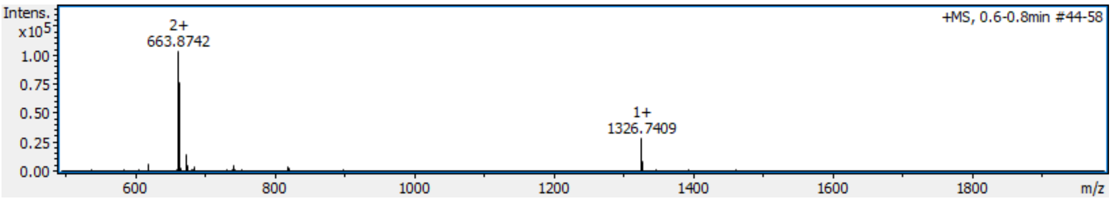

Figure S44 - Analytical HPLC trace of pure product **31**. Analytical gradient 10-30% B over 10 min, 210 nm. Calculated Mass $[M+H]^+$: 1326.74, $[M+2H]^{2+}$: 663.87. Observed Mass $[M+H]^+$: 1326.74, $[M+2H]^{2+}$: 663.87.

H-HRLLRCGNYA-NH2 (23) + 4-(allylamino)-4-oxobutanoic acid (21) – photochemistry set up 2

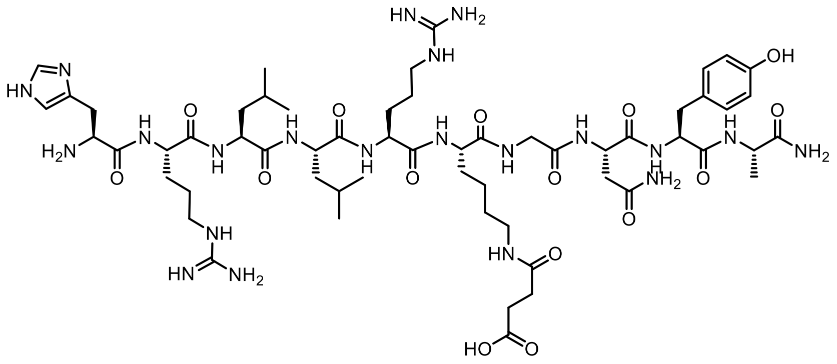

Peptide **31** was synthesised following the general conjugation protocol B (set up 2) using H-HRLLRCGNYA-NH2 (**23**, 2 mg, 1.66 µmol) and 4-(allylamin)-4-oxobutanoic acid (**21,** 52 mg, 0.33 mmol). After analysis the remainder of material (1.565 µmol) was purified using purified using semi-preparative HPLC (5-40% B over 30 minutes) to yield the desired conjugated peptide (**31**, 1.0 mg, 0.754 µmol, 48% yield).

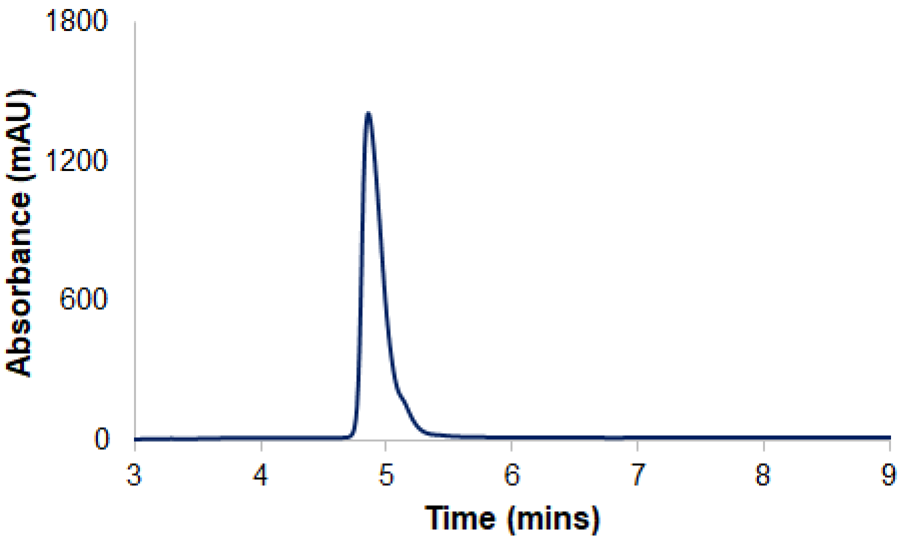

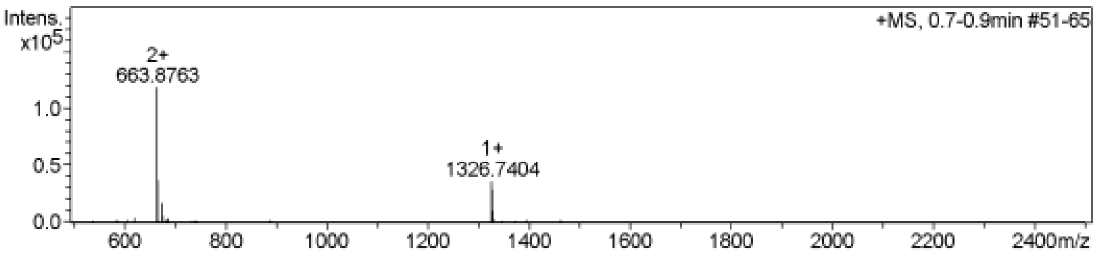

Figure S45 - Analytical HPLC trace pure product **31**. Analytical gradient 10-30% B over 10 min, 210 nm. Calculated Mass $[M+H]^+$: 1326.74, $[M+2H]^{2+}$: 663.87. Observed Mass $[M+H]^+$: 1326.74, $[M+2H]^{2+}$: 663.88.

H-HRLLRCGNYA-NH2 (23) + *N*-allylbenzenamide (22) – photochemistry set up 1

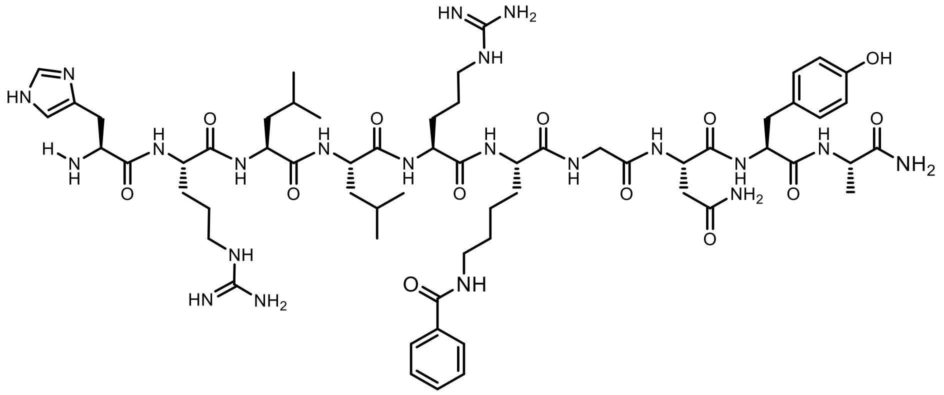

Peptide **32** was synthesised following the general conjugation protocol B (set up 1) using H-HRLLRCGNYA-NH2 (**23**, 2 mg, 1.66 µmol) and *N*-allylbenzenamide (**22**, 53 mg, 0.33 mmol). After analysis the remainder of material (1.565 µmol) was purified using purified using semi-preparative HPLC (5-40% B over 30 minutes) to yield the desired conjugated peptide (**32**, 1.3 mg, 0.977 µmol, 62% yield).

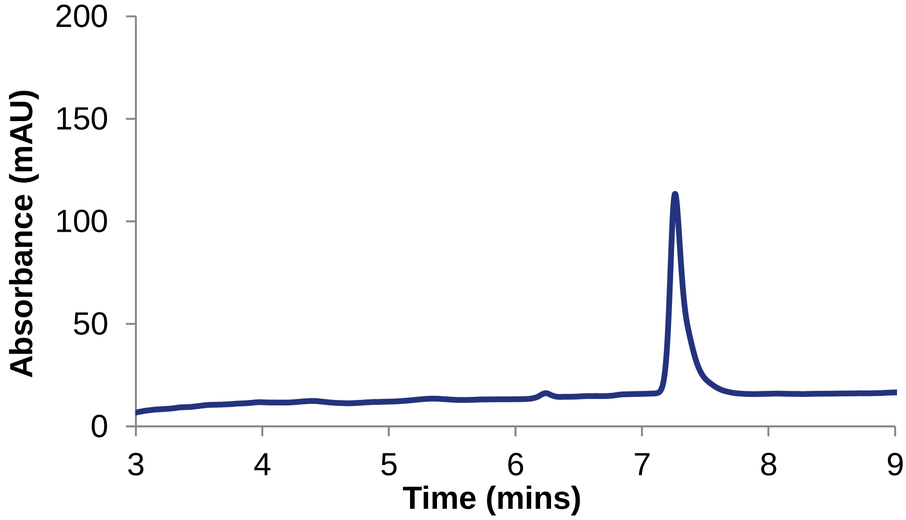

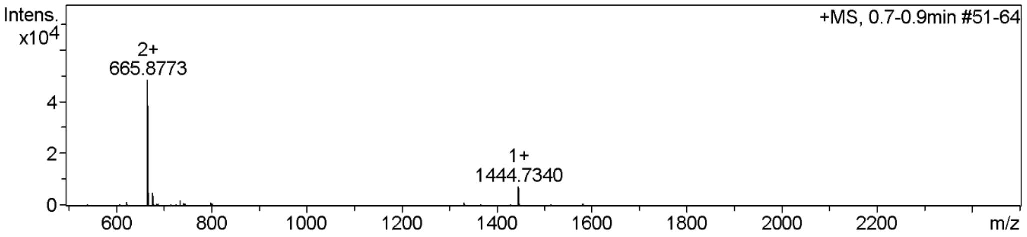

Figure S46 - Analytical HPLC trace of pure product **32**. Analytical gradient 10-30% B over 10 min, 210 nm. Calculated Mass [M+H+TFA]$^{+}$: 1444.74, [M+2H]$^{2+}$: 665.88. Observed Mass [M+H+TFA]$^{+}$: 1444.73, [M+2H]$^{2+}$: 665.88.

H-HRLLRCGNYA-NH2 (23) + *N*-allylbenzenamide (22) – photochemistry set up 2

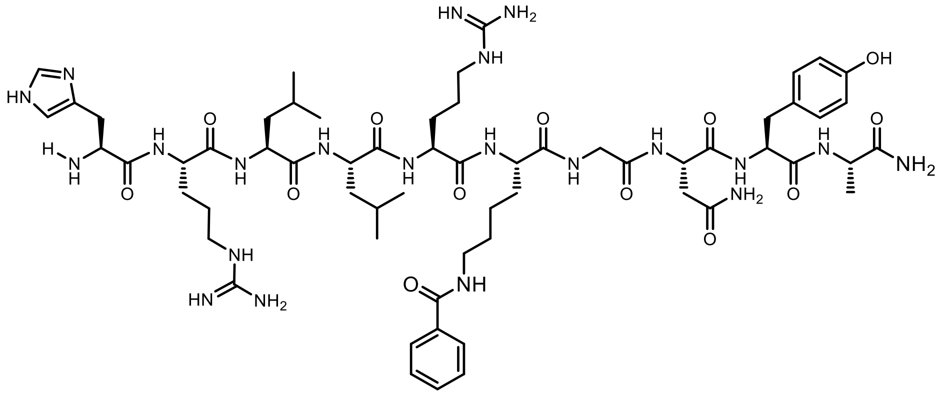

Peptide **32** was synthesised following the general conjugation protocol B (set up 2) using H-HRLLRCGNYA-NH2 (**23**, 2 mg, 1.66 µmol) and *N*-allylbenzenamide (**22**, 53 mg, 0.33 mmol). After analysis the remainder of material (1.565 µmol) was purified using purified using semi-preparative HPLC (5-40% B over 30 minutes) to yield the desired conjugated peptide (**32**, 1.2 mg, 0.902 µmol, 58% yield).

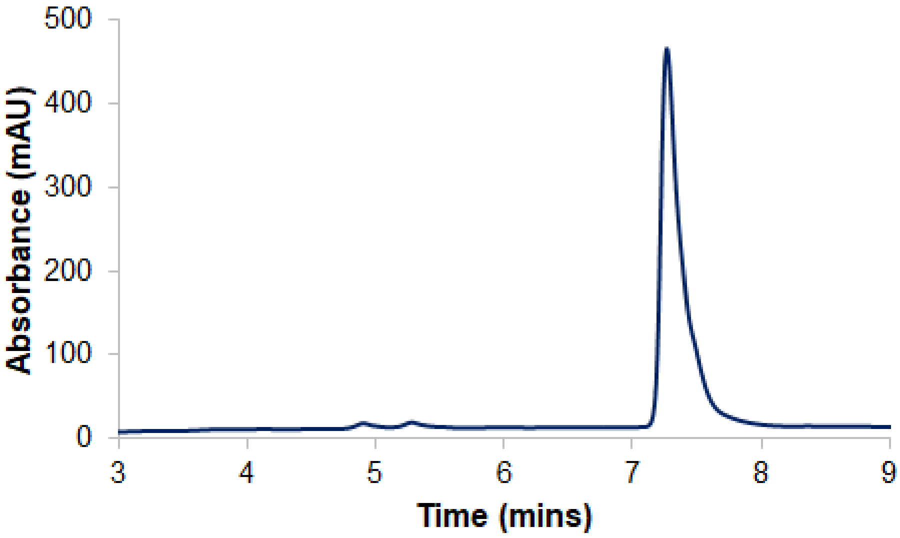

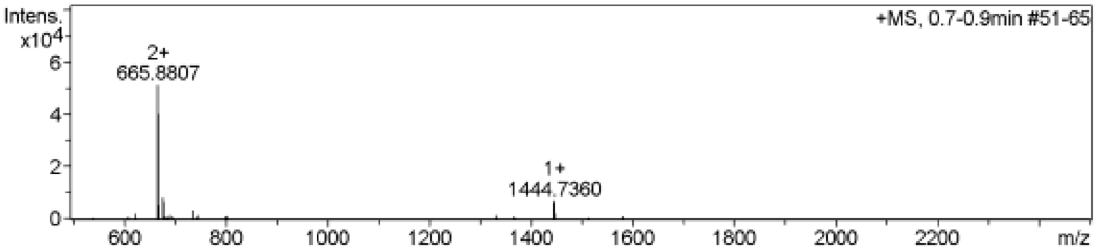

Figure S47 - Analytical HPLC trace of pure product **32.** Analytical gradient 10-30% B over 10 min, 210 nm. Calculated Mass [M+H+TFA]$^{+}$: 1444.74, [M+2H]$^{2+}$: 665.88. Observed Mass [M+H+TFA]$^{+}$: 1444.74, [M+2H]$^{2+}$: 665.88.

Site-selective modification of a cyclic peptide

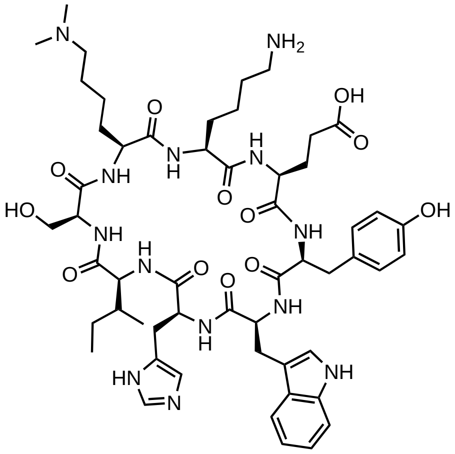

Peptide **34** was synthesized following the general conjugation protocol B (set up 2) using c[WHISCKEY] (**33**, 3 mg, 2.87 µmol) and dimethylallylamine (**16**, 66 mg, 0.78 mmol). After analysis the remainder of material (2.84 µmol) was purified using semi-preparative HPLC (15-60% B over 30 minutes) to yield the desired conjugated peptide (**34**, 1.45 mg, 1.32 µmol, 46% yield).

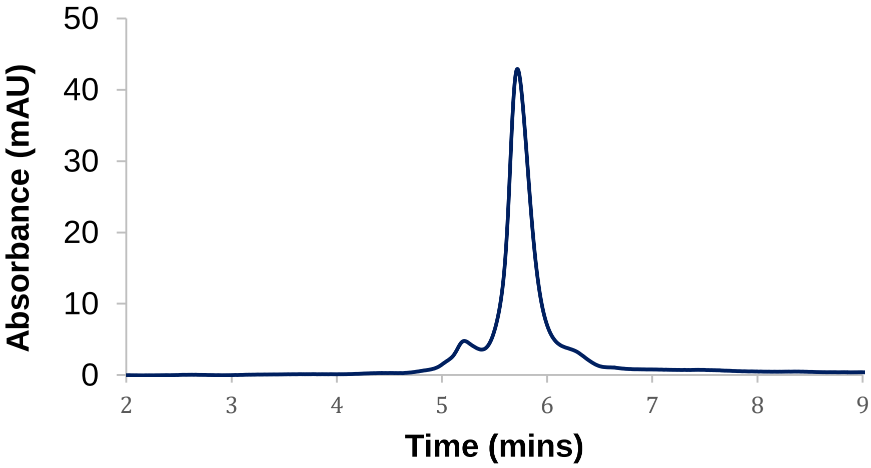

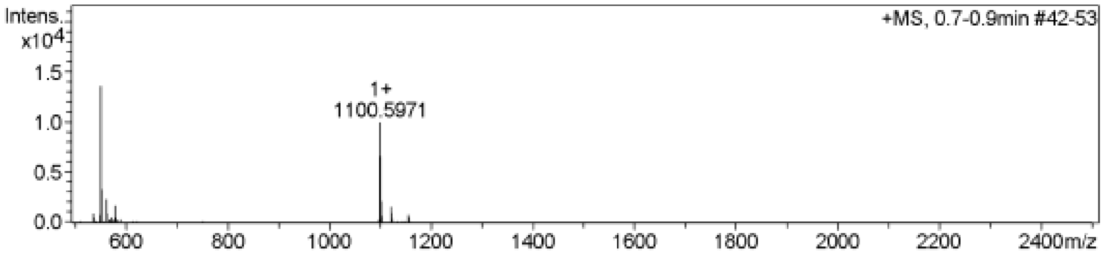

Figure S48 - Analytical HPLC trace of pure product **34**. Analytical gradient 5-60% B over 10 min, 280 nm. Calculated Mass $[M+H]^+$: 1100.58. Observed Mass $[M+H]^+$: 1100.59.

Site-selective installation of N^{ε}Ac Lys into K48C ubiquitin

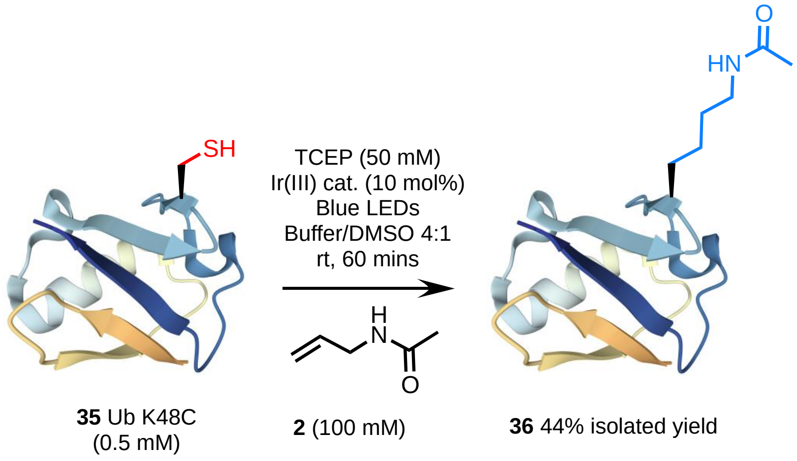

Ub K48C **(35)**

H-MQIFVKTLTGKTITLEVESSDTIDNVKSKIQDKEGIPPDQQRLIFAG**C**QL
EDGRTLSDYNIQKESTLHLVLRLRGG-OH

N^{ε}Ac-Ub (**36**) was synthesised following the general conjugation protocol C (set up 1) using K48C ubiquitin (**35**, 4 mg, 0.469 µmol) and *N*-allyl acetamide (**2**, 9 mg, 94 µmol). After analysis the remainder of material (0.444 µmol) was purified using purified using semi-preparative HPLC (25-35% B over 60 minutes) to yield the desired conjugated peptide (**36**, 1.7 mg, 0.197 µmol, 44% yield).

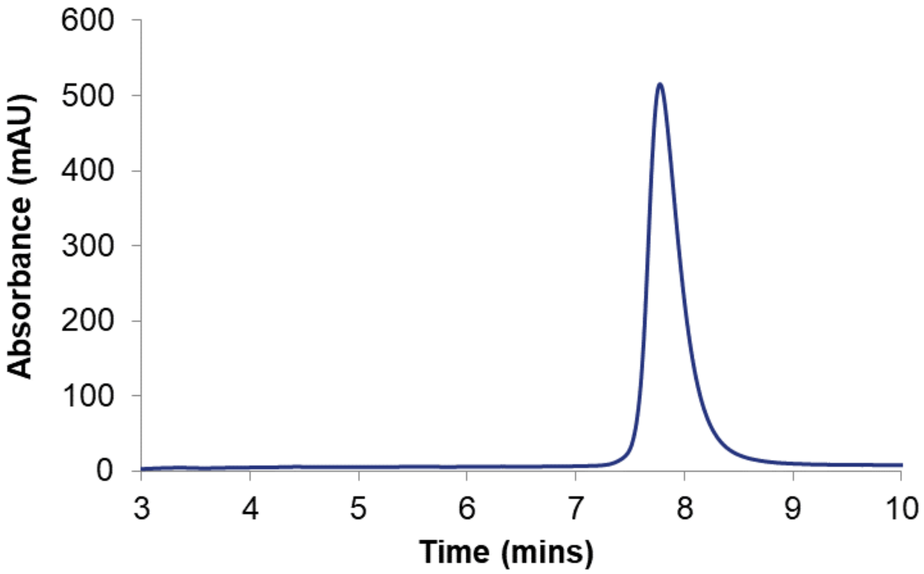

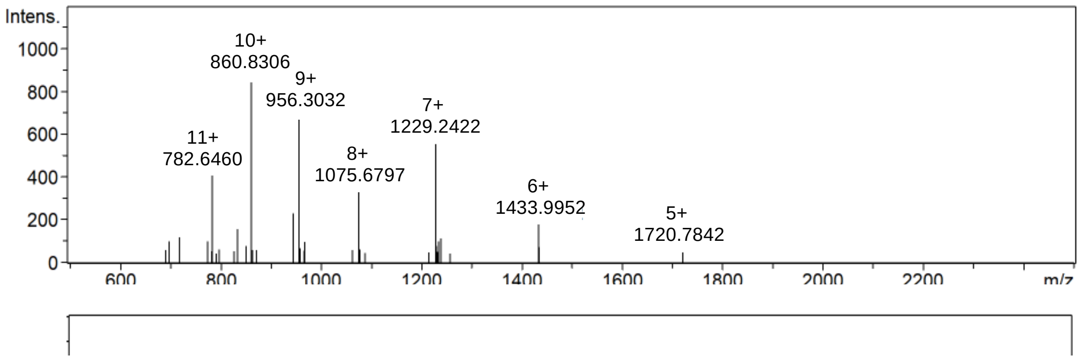

Figure S49 - Analytical HPLC trace of pure product **36**. Analytical gradient 25-35% B over 10 min, 210 nm. Calculated Mass $[M+6H]^{6+}$: 1433.27, $[M+7H]^{7+}$: 1228.66, $[M+8H]^{8+}$: 1075.21, $[M+9H]^{9+}$: 955.85, $[M+10H]^{10+}$: 860.37, $[M+11H]^{11+}$: 782.24. Observed Mass $[M+6H]^{6+}$: 1433.99, $[M+7H]^{7+}$: 1229.24, $[M+8H]^{8+}$: 1075.68, $[M+9H]^{9+}$: 956.30, $[M+10H]^{10+}$: 860.83, $[M+11H]^{11+}$: 782.65.

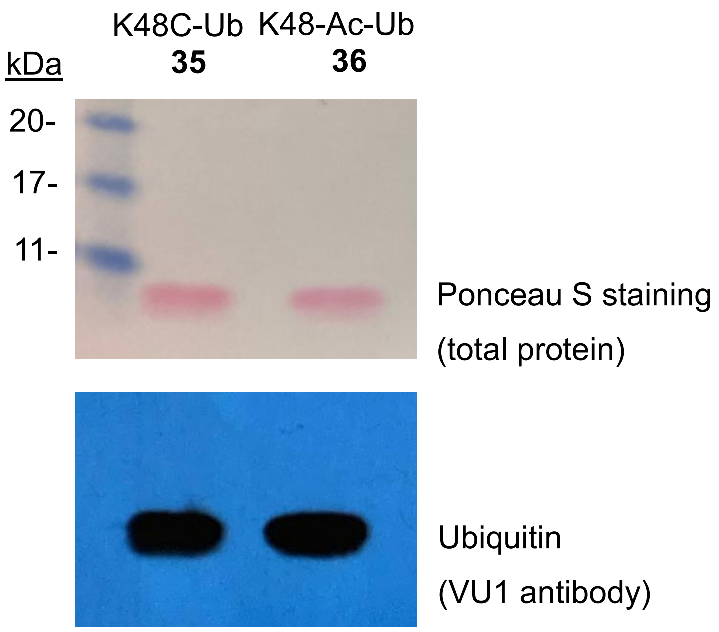

Figure S50 – SDS-PAGE Western Blot analysis of K48C Ub **35** and NAc-Ub **36**; total protein stain and recognition using anti-Ub antibody (VU1, 06-933 Sigma-Aldrich) (Scott, D. *et al.*, *Proteomics* **2016** 16(14):1961-9).

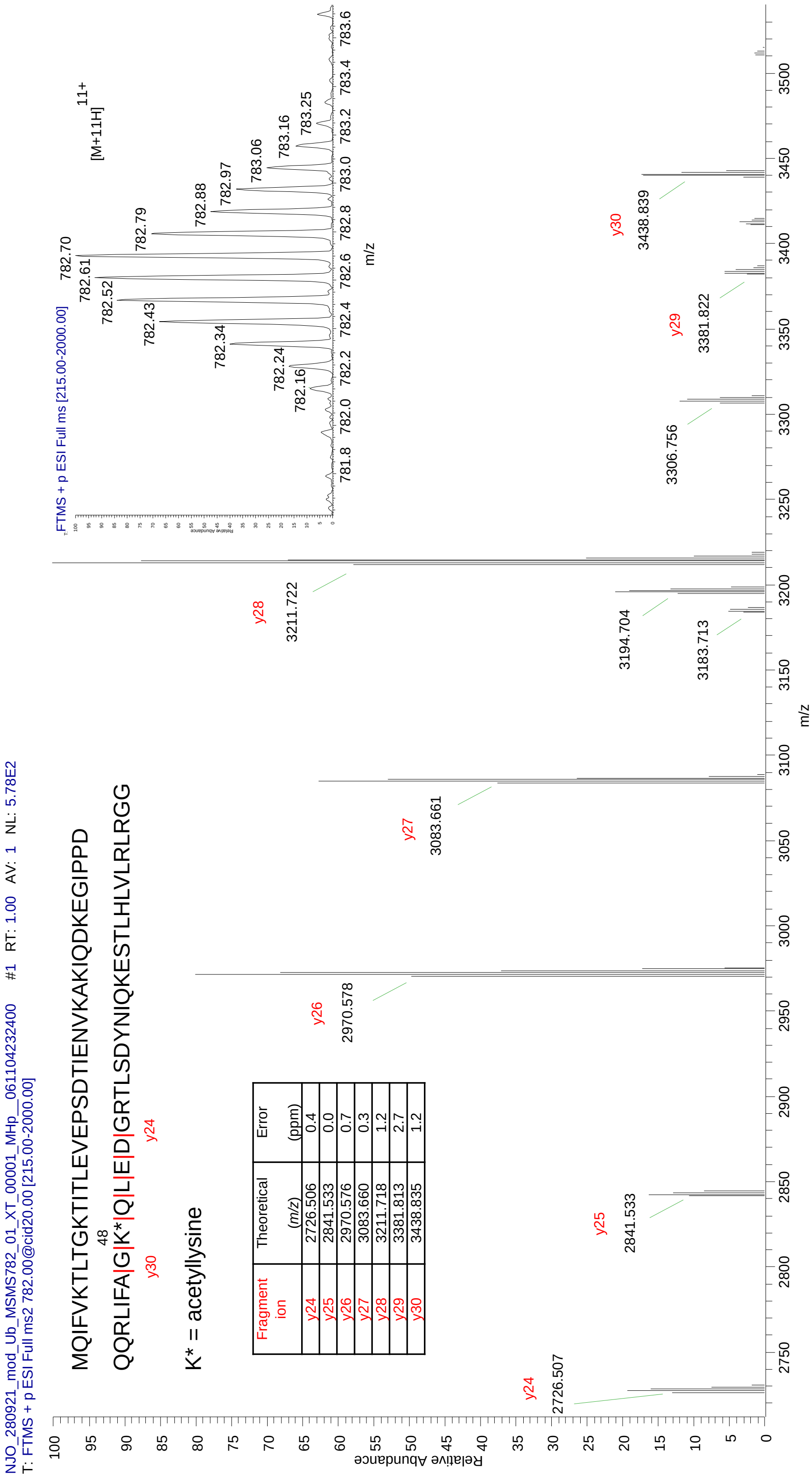

Figure S51 – Deconvoluted ESI-MS/MS spectrum of the $[M+11H]^{11+}$ ion of N^{ε}Ac-Ub (**36**) (*m/z* 782) showing the y-ion fragments y24 to y30, which locate acetylation to residue K48. Insert shows the $[M+11H]^{11+}$ ion of **36** used as the precursor for the MS/MS experiment. Deconvoluted ions are shown as singly-charged $[M+H]^{+}$.

^{1}H NMR analysis of folded N^{ε}Ac-Ub

Purified N^{ε}Ac-Ub **36** was dissolved in 6 M Gdn•HCl, 0.1 M Na_2HPO_4, pH 7 and dialysed into 25 mM Na_2PO_4, 100 mM NaCl, pH 7.0 overnight. 10 vol% D_2O was added to the sample which was analyzed on a Bruker 600 MHz Avance III NMR system using excitation sculpting for solvent suppression. Data were zero-filled and a 2 Hz line broadening applied prior to Fourier Transform and baseline correction. Data were referenced indirectly using the internal D_2O signal that was calibrated to give 0 ppm for TMS, following the methods detailed by IUPAC.

The extended NH region (6.5 - 9.5 ppm, figure S30) is consistent with folded Ub (BMRB 68 and 4769). The upfield Me signals (figure S32) confirm hydrophobic packing.

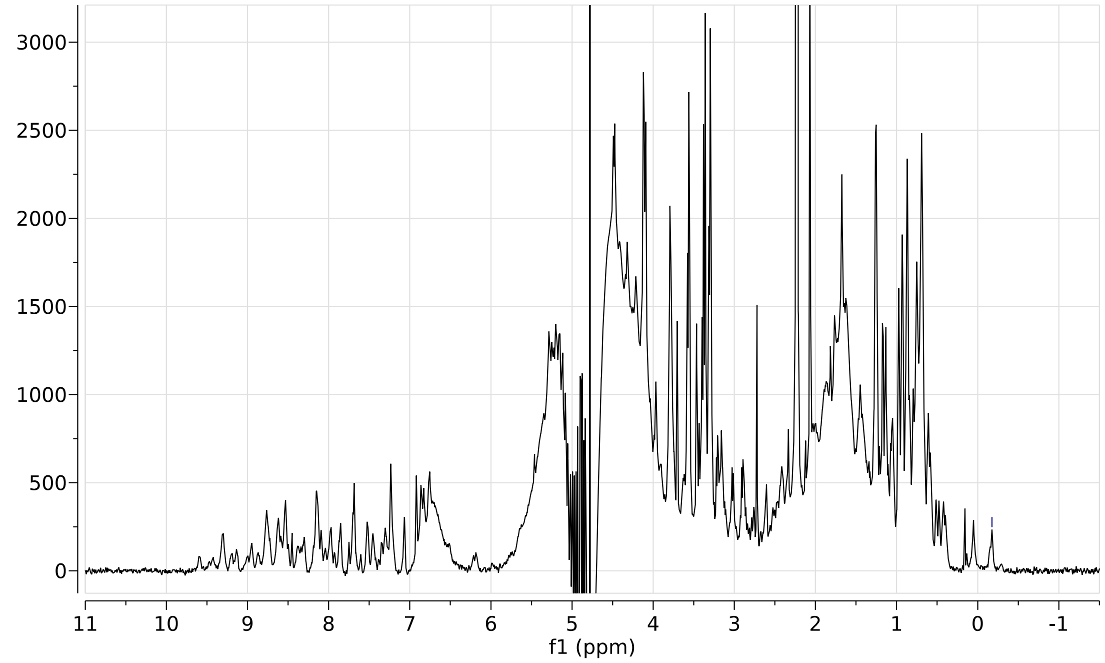

Figure S52 – ^{1}H NMR spectra for N^{ε}Ac-Ub (**36**); NH region extended across 6.5-9.5 ppm indicates a tertiary fold.

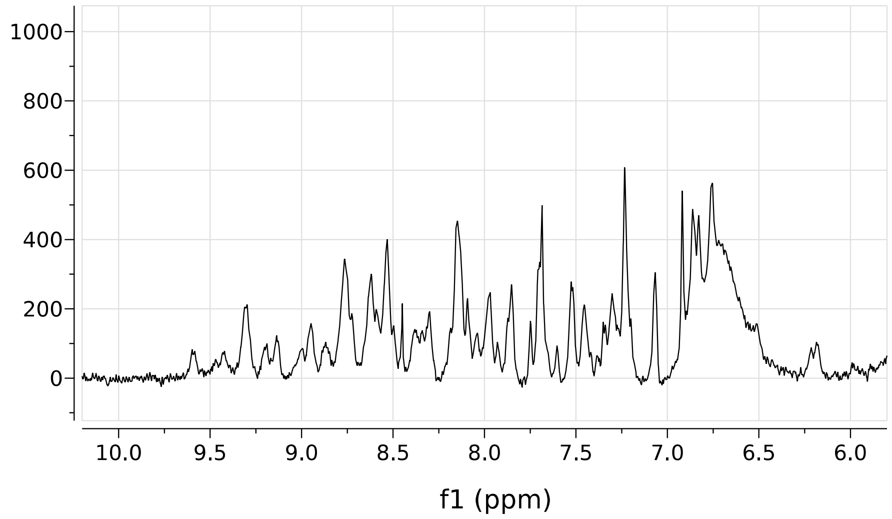

Figure S53 – Expansion of the NH region; extension of these signals from 6.5-9.5 is indicative of a tertiary fold.

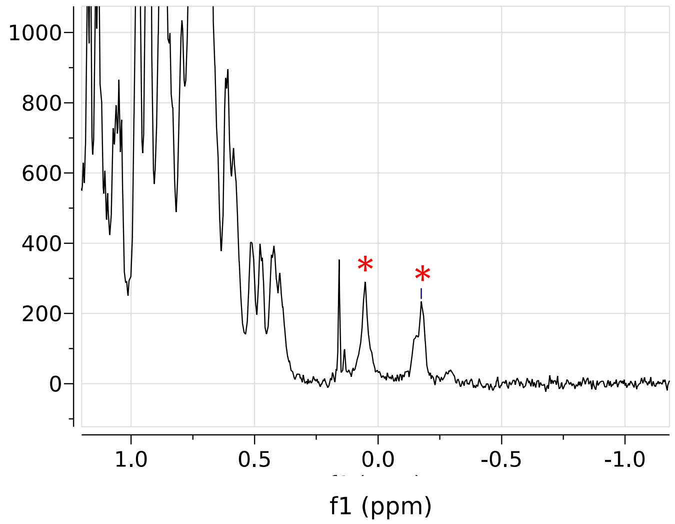

Figure S54 – Expansion of the upfield region – shifted Me signals indicate hydrophobic packing consistent with a tertiary fold.

Appendix

N-allylacetamide (**2**)

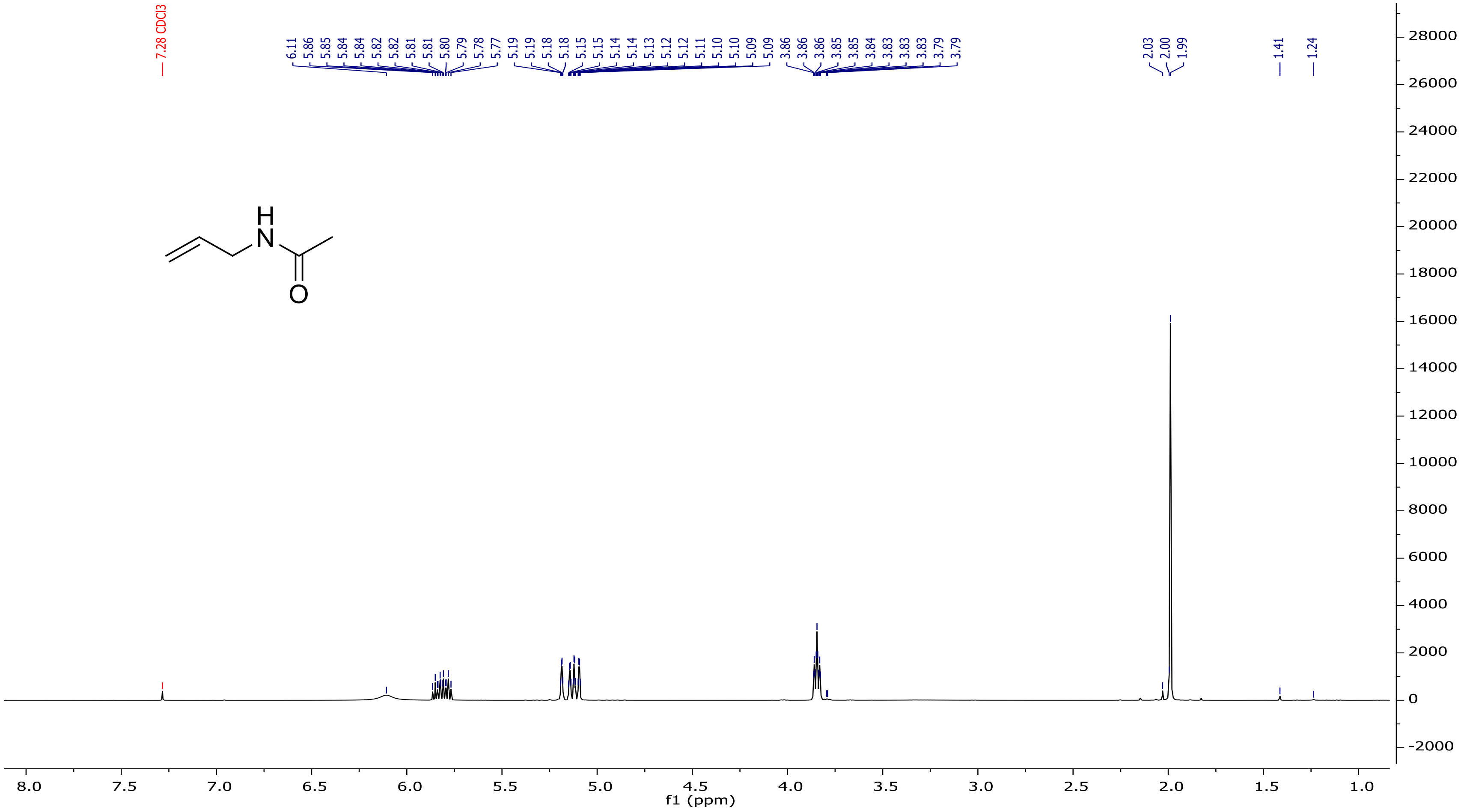

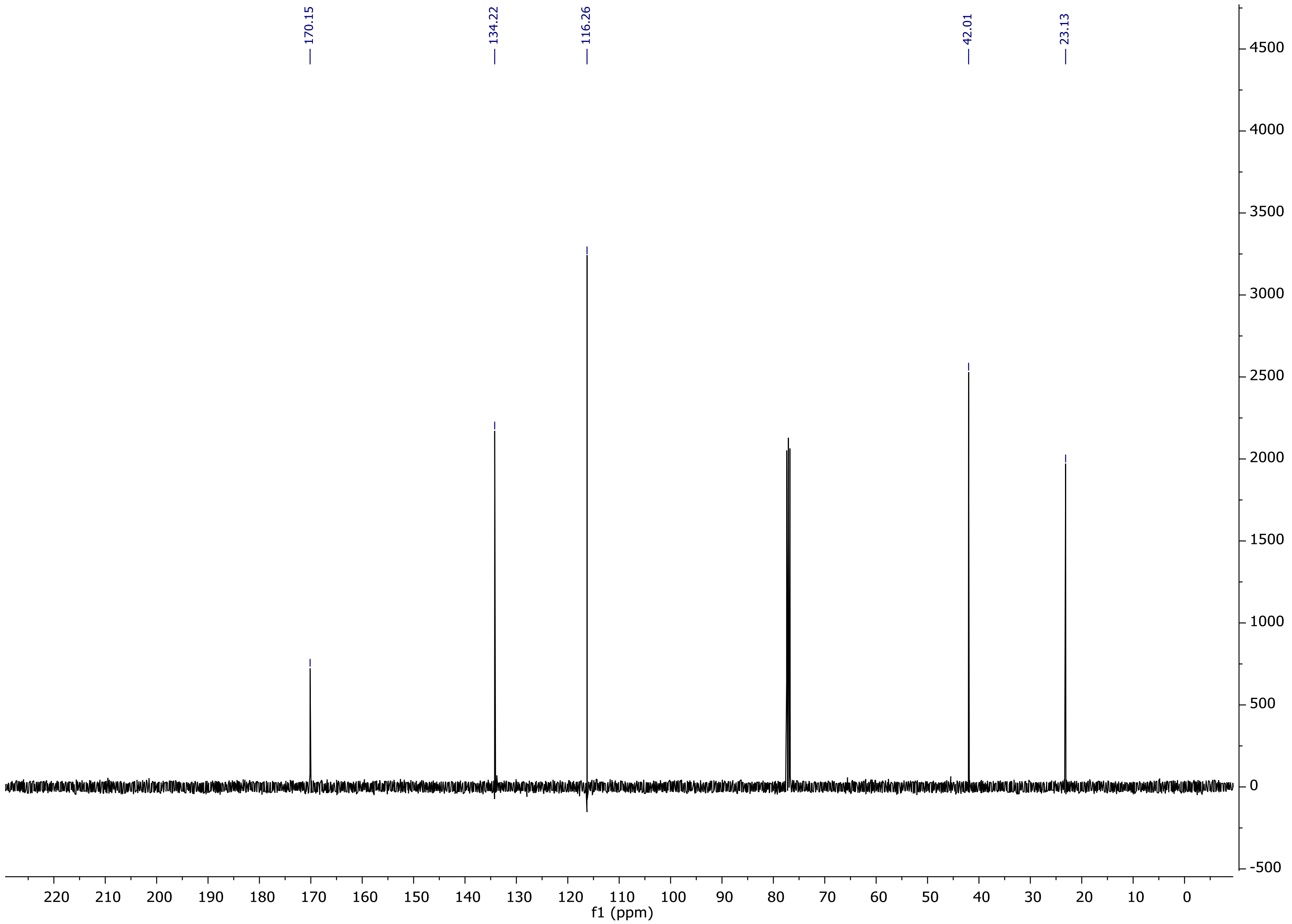
170.15
134.22
116.26
42.01
23.13
4500
4000
3500
3000
2500
2000
1500
1000
500
0
-500
220 210 200 190 180 170 160 150 140 130 120 110 100 90 80 70 60 50 40 30 20 10 0
f1 (ppm)

Allyltrimethylammonium chloride (**17**)

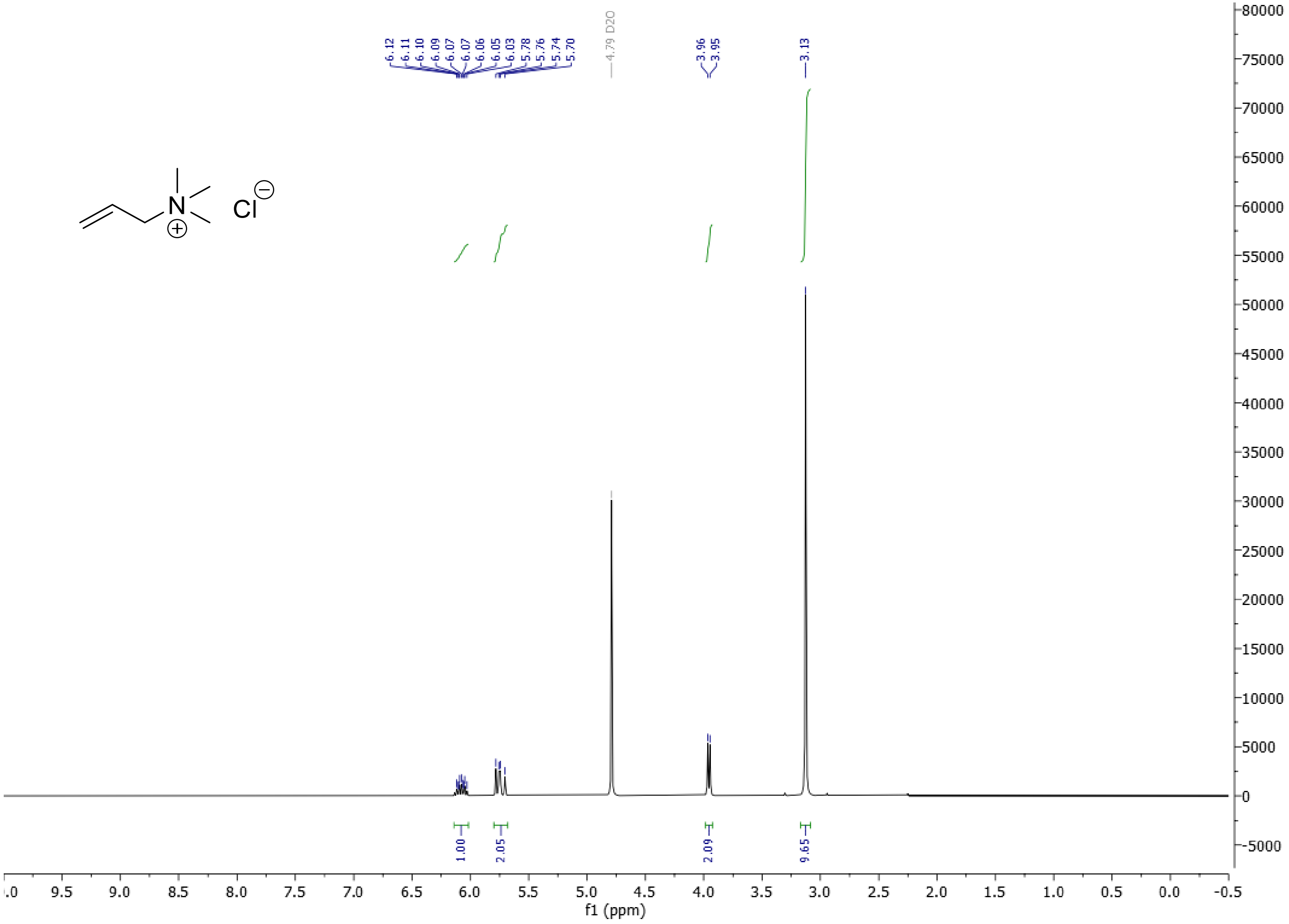

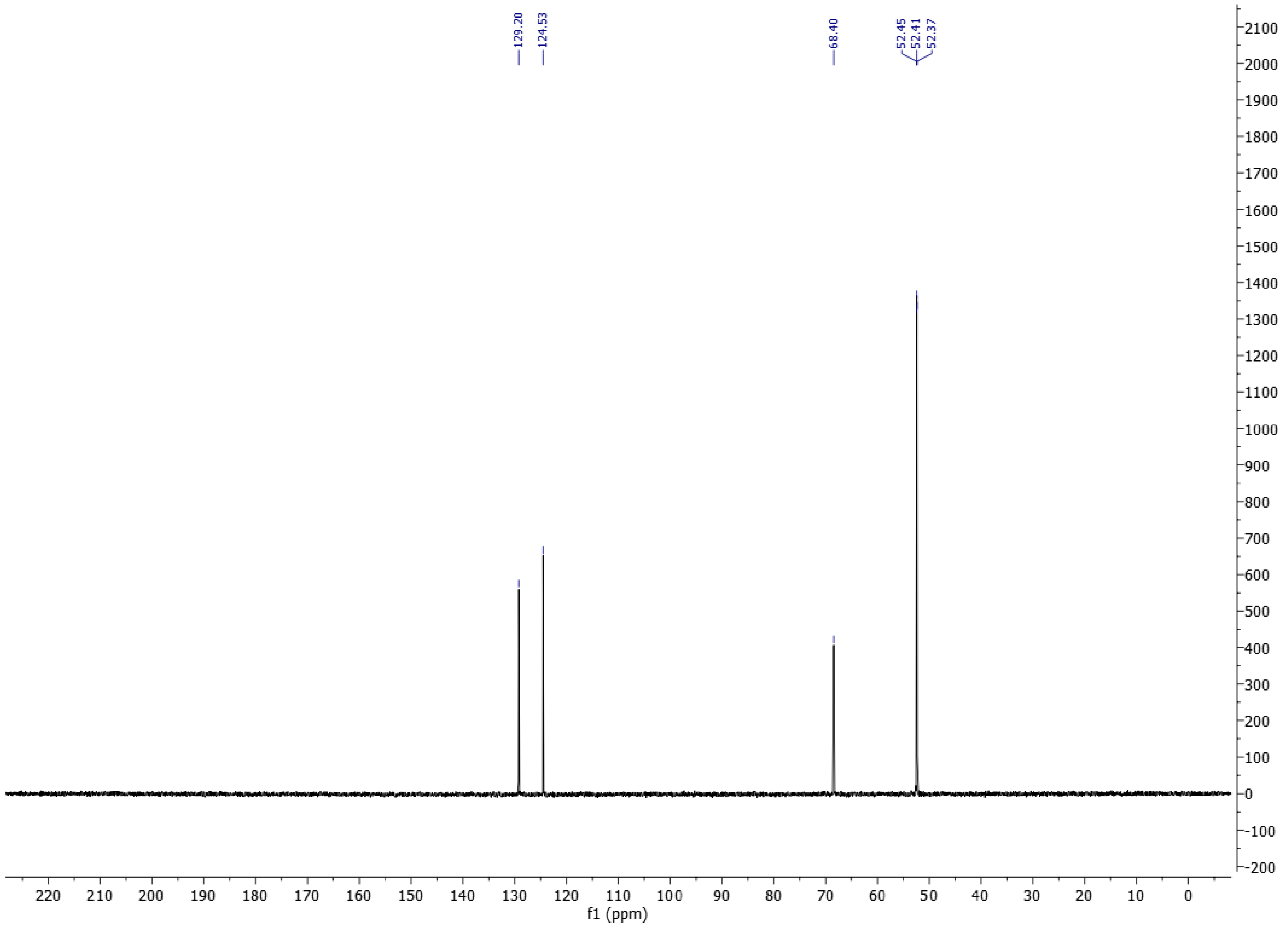
129.20
124.53
68.40
52.45
52.41
52.37
f1 (ppm)

N-allylformamide (**18**)

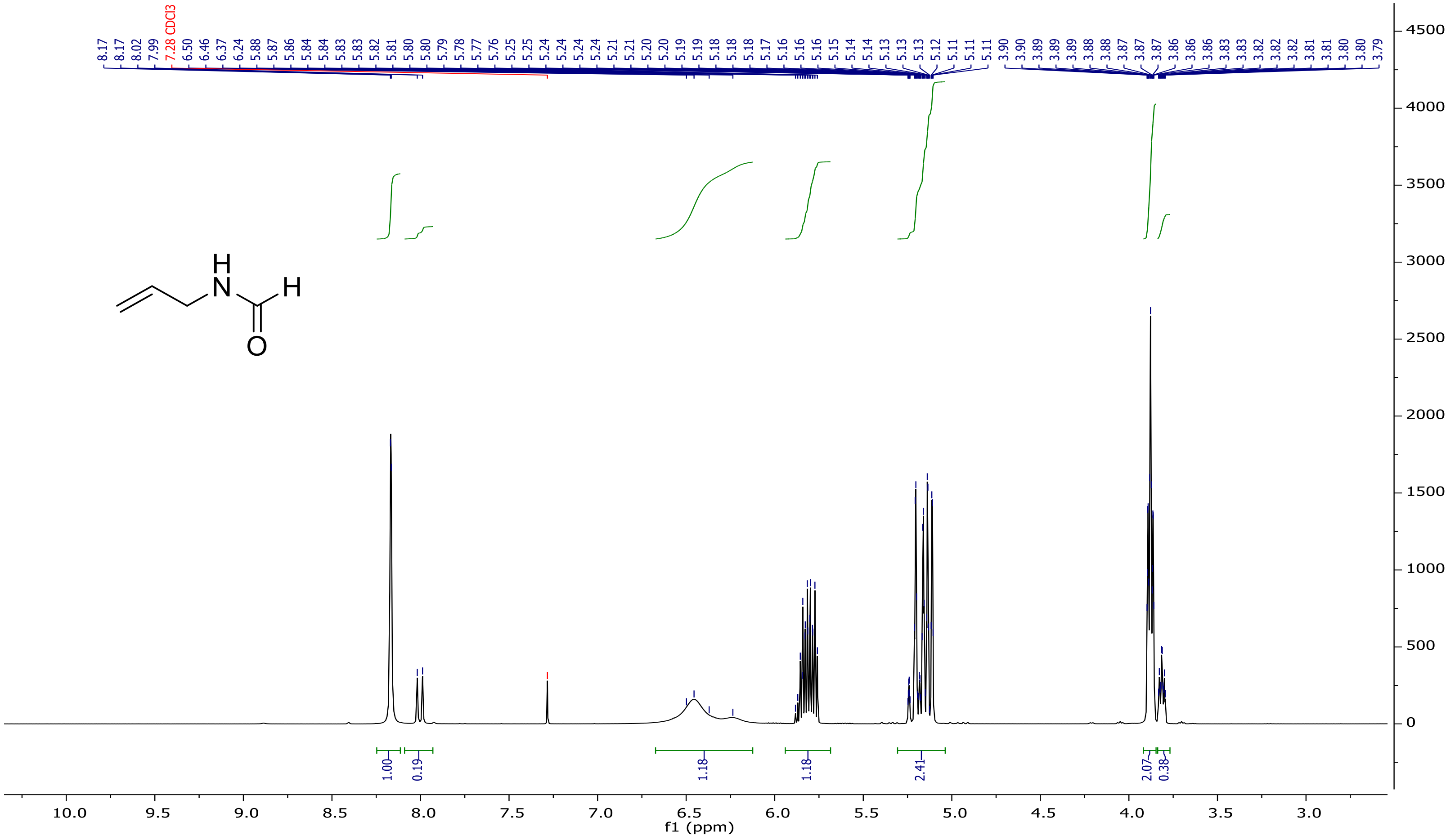

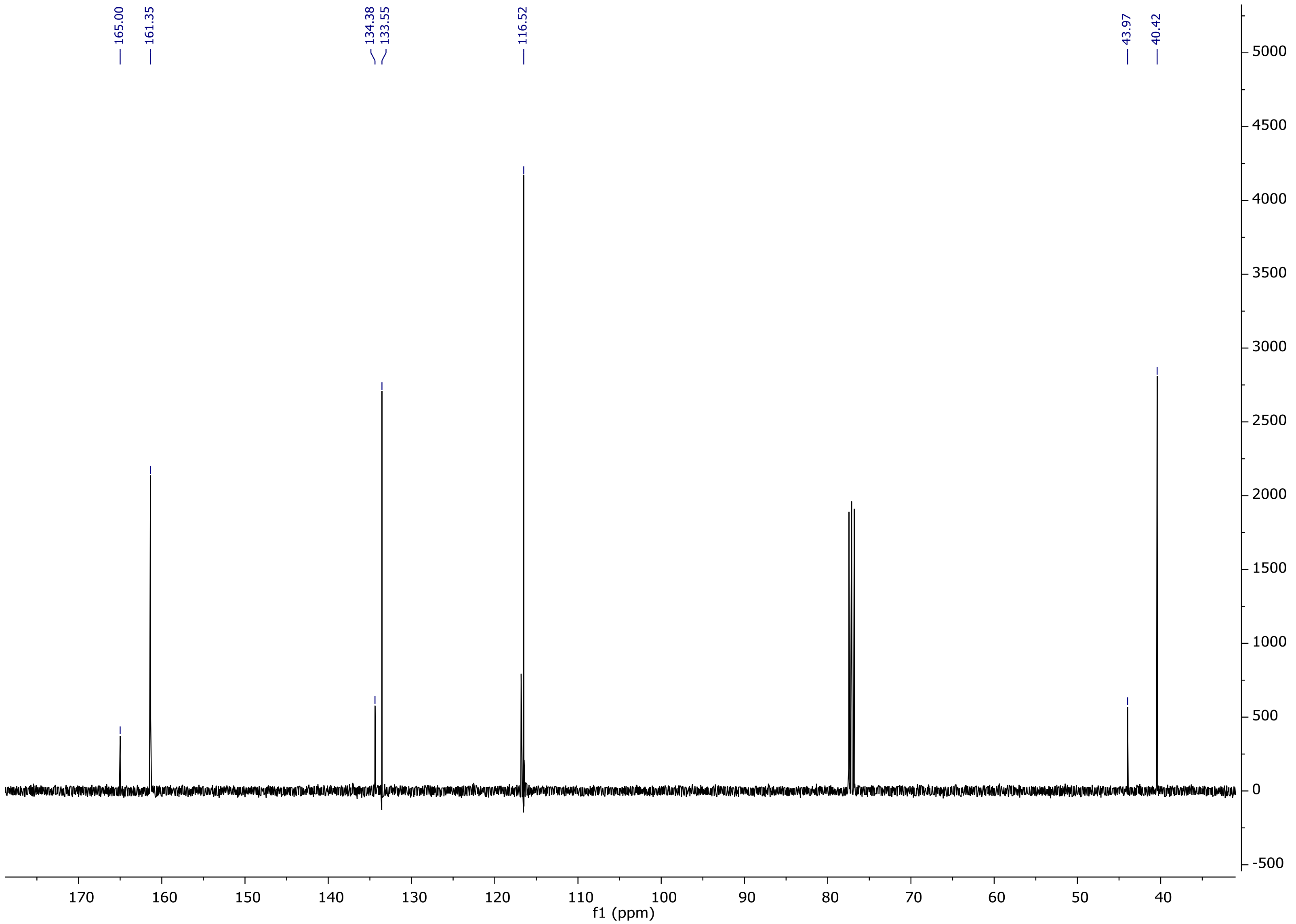
165.00
161.35
134.38
133.55
116.52
43.97
40.42
5000
4500
4000
3500
3000
2500
2000
1500
1000
500
0
-500
170
160
150
140
130
120
110
100
90
80
70
60
50
40
f1 (ppm)

N-Allylpropionamide (**19**)

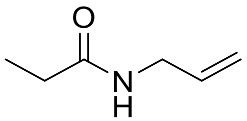

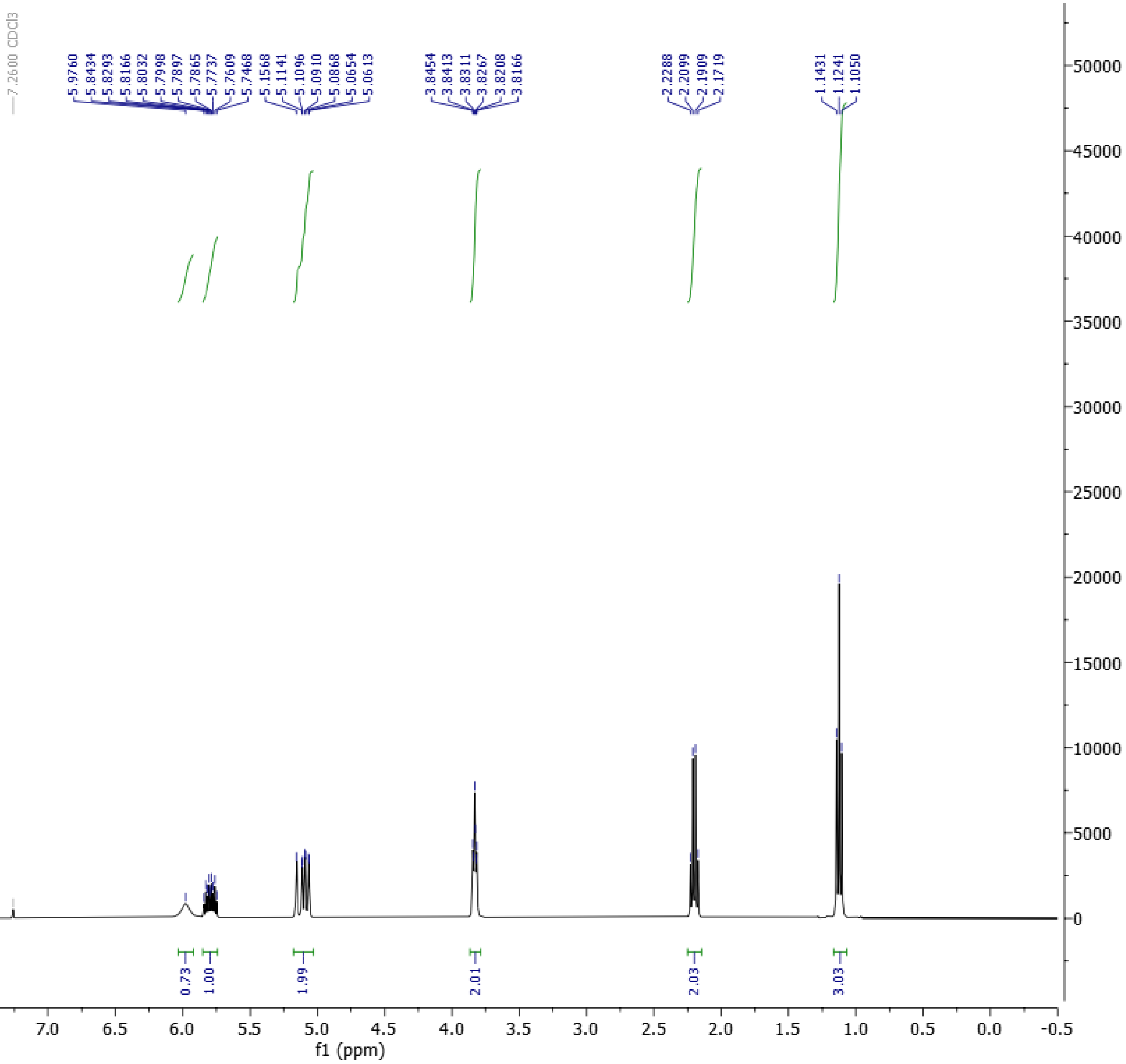

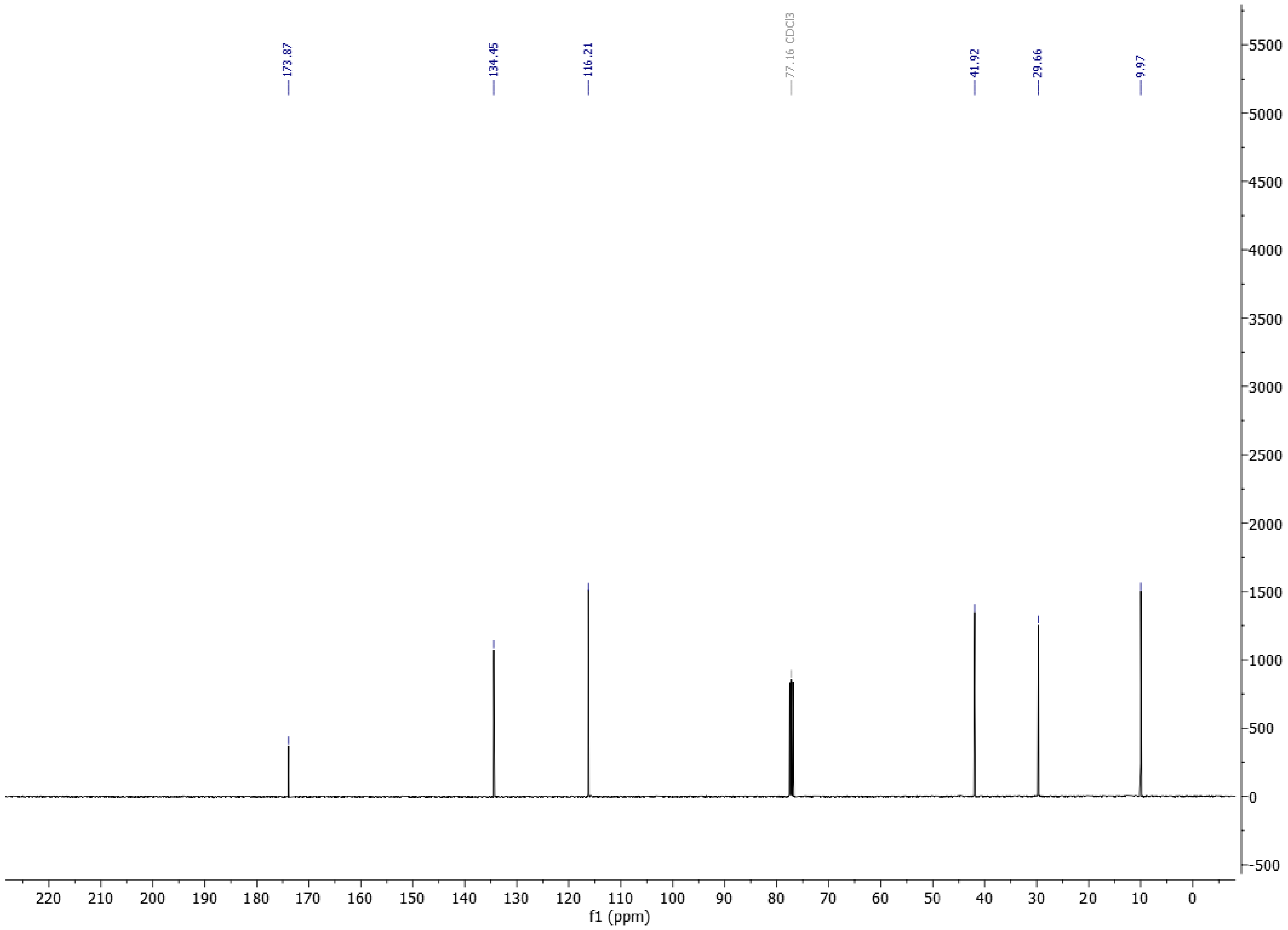
173.87
134.45
116.21
77.16 CDCl3
41.92
29.66
9.97
f1 (ppm)

N-Allyl-2-hydroxy-2-methylpropanamide (**20**)

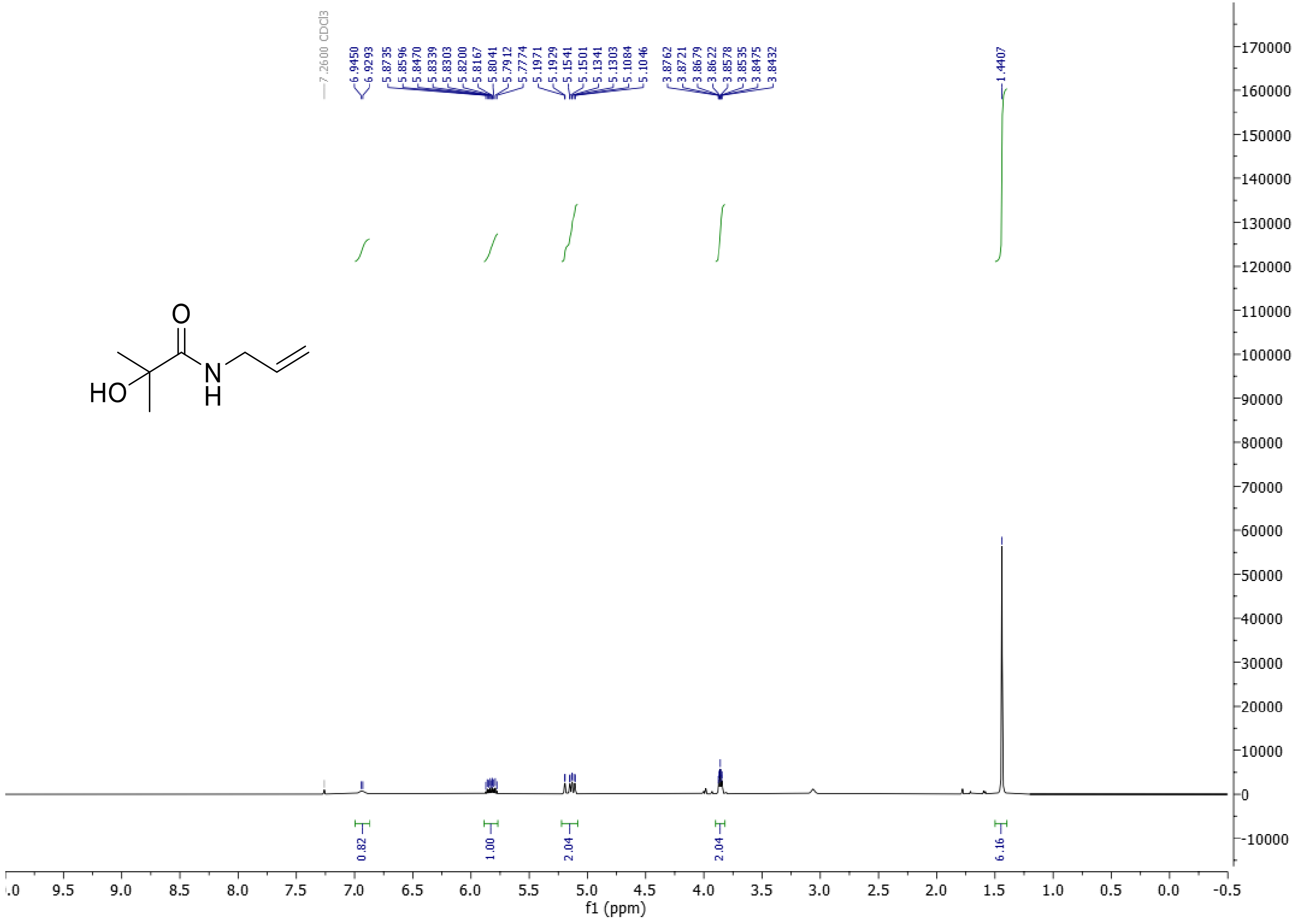

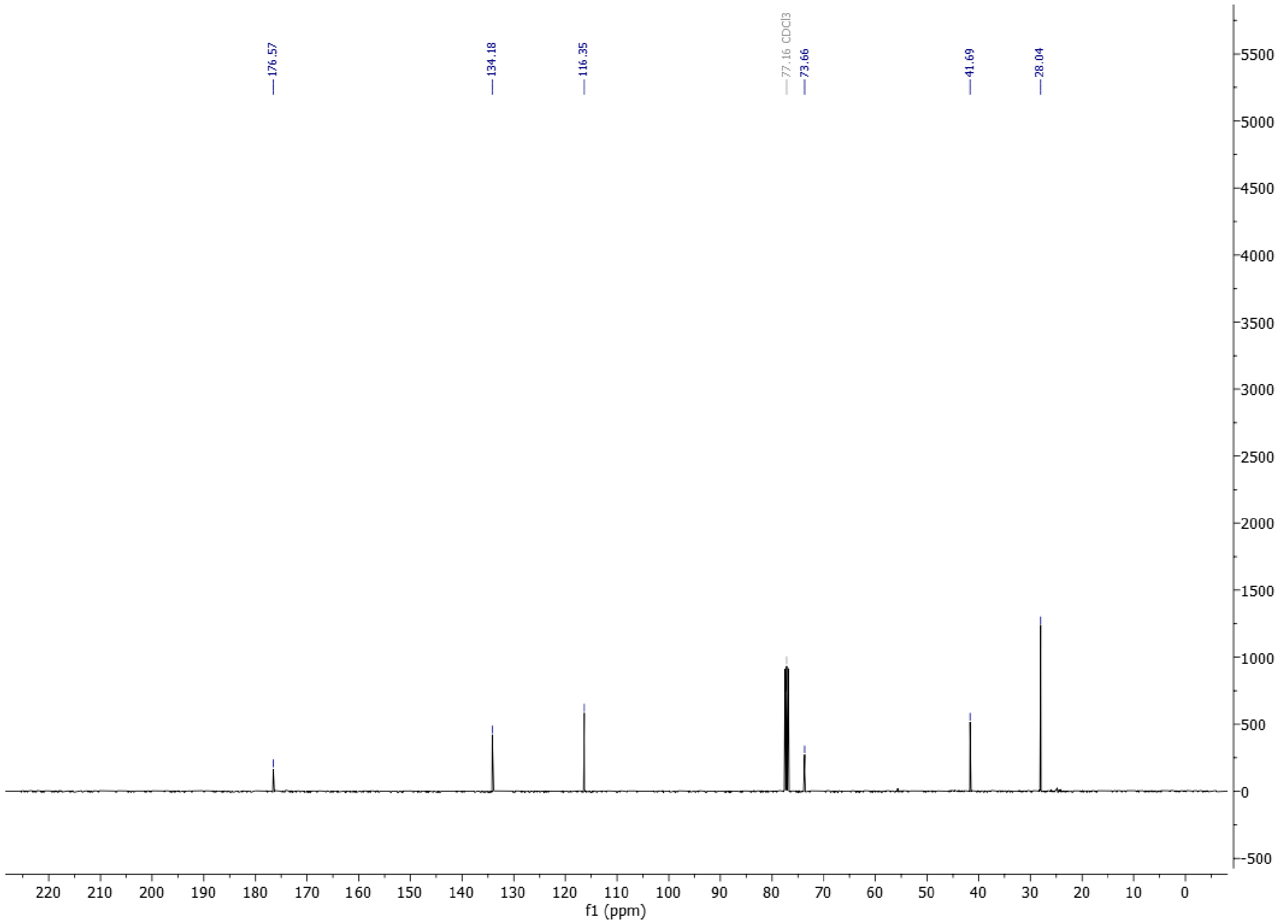
176.57
134.18
116.35
77.16 CDCl3
73.66
41.69
28.04
f1 (ppm)

4-(allylamino)-4-oxobutanoic acid (**21**)

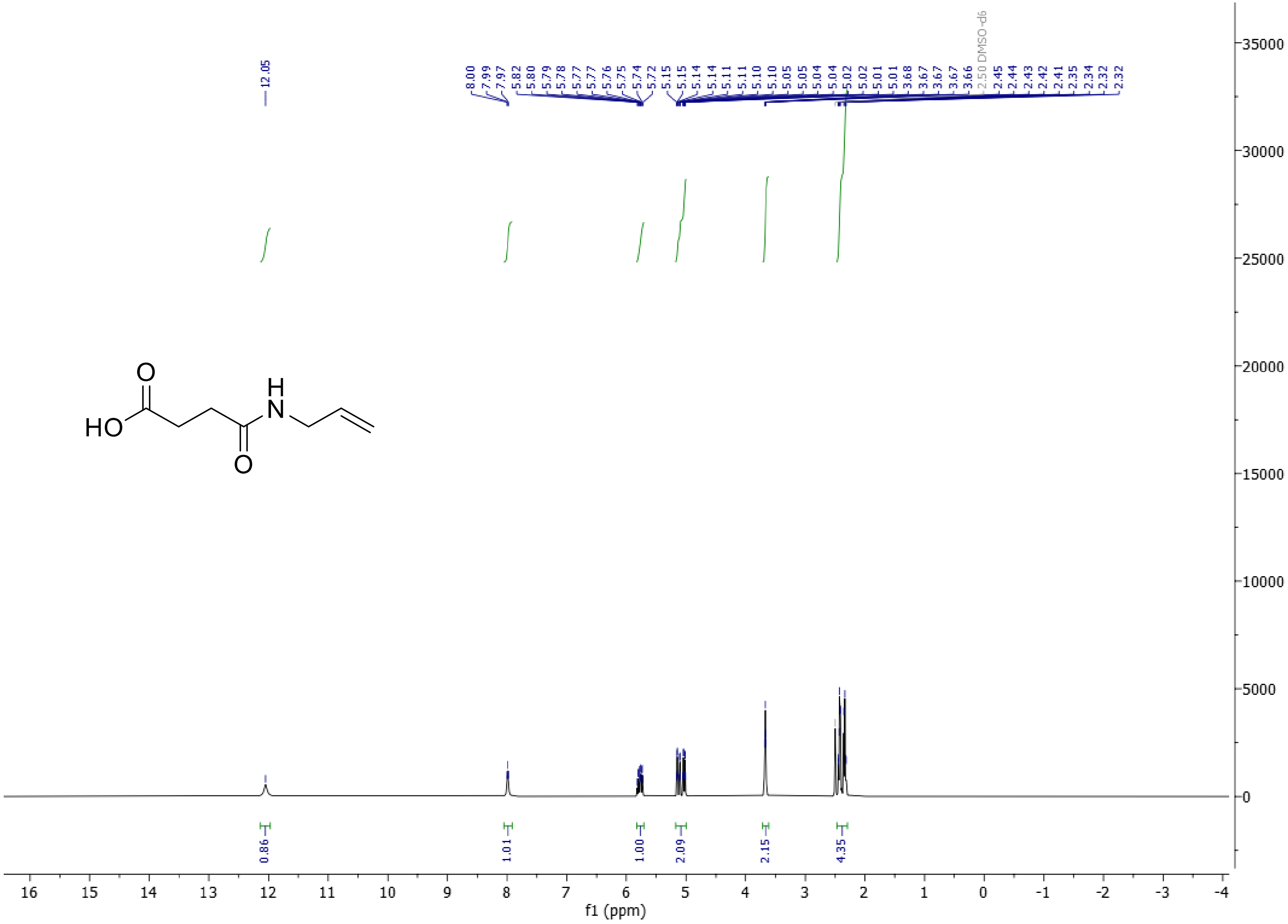

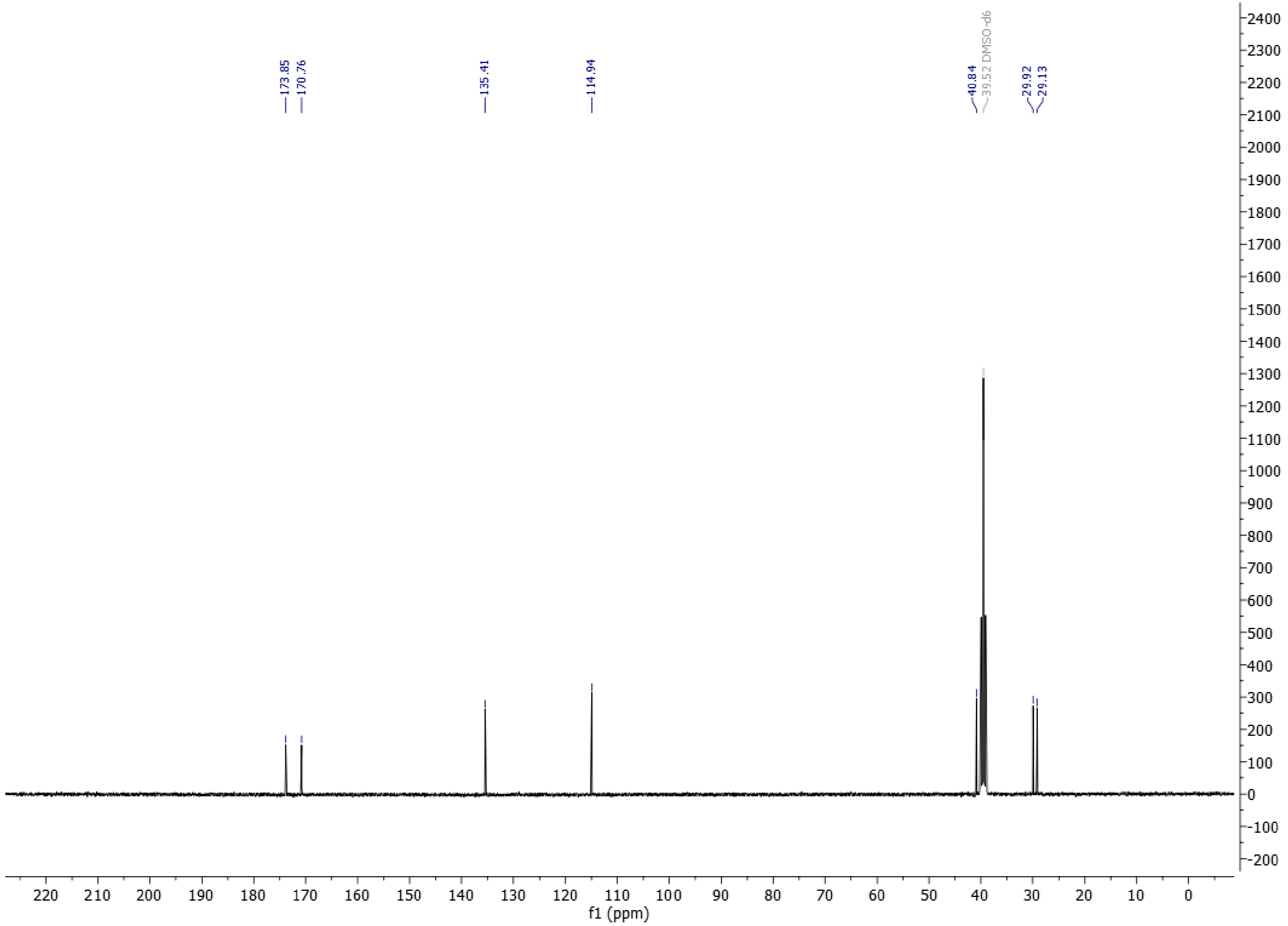

173.85
170.76
135.41
114.94
40.84
39.52 DMSO-d6
29.92
29.13
f1 (ppm)

N-allylbenzamide (**22**)

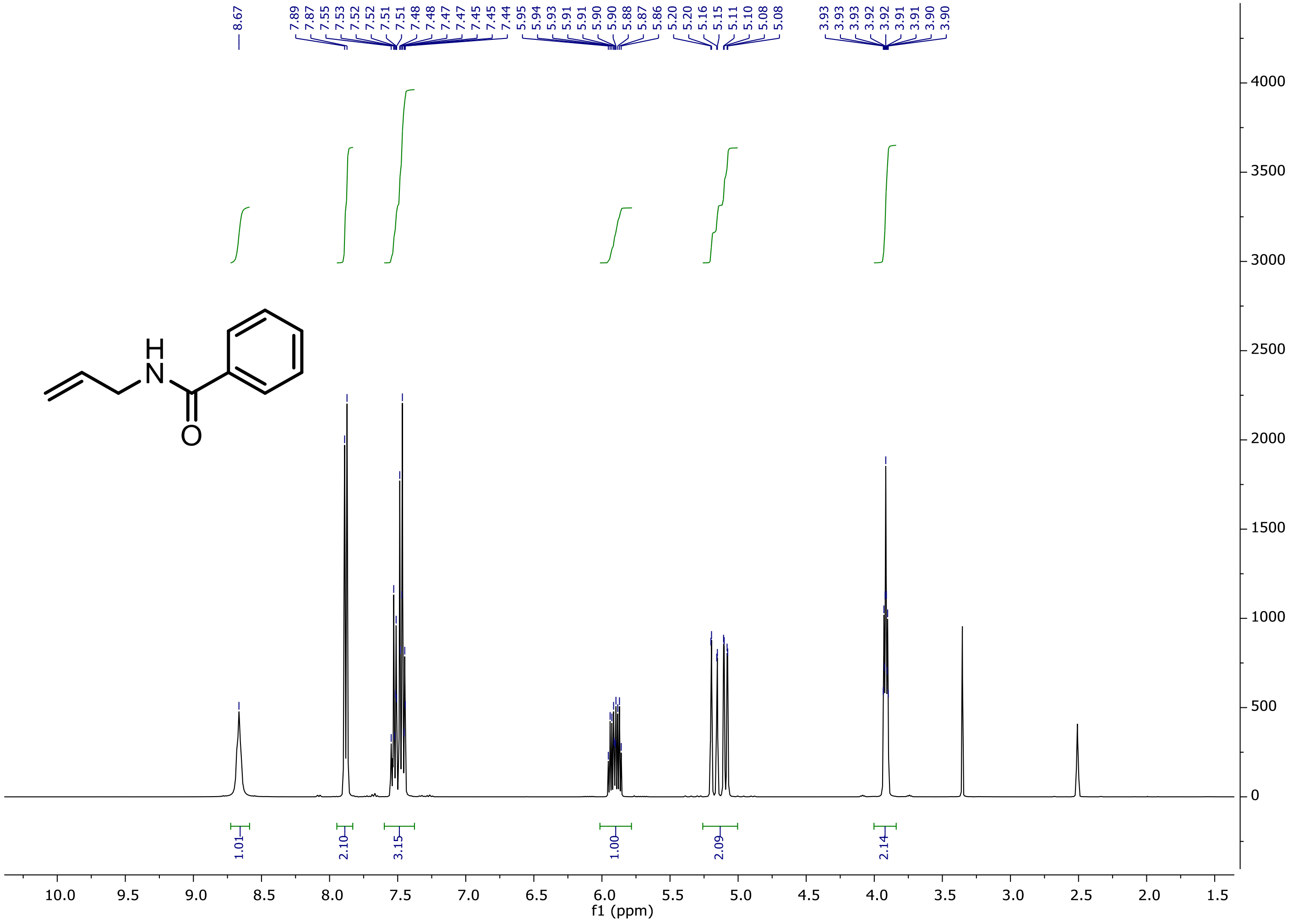

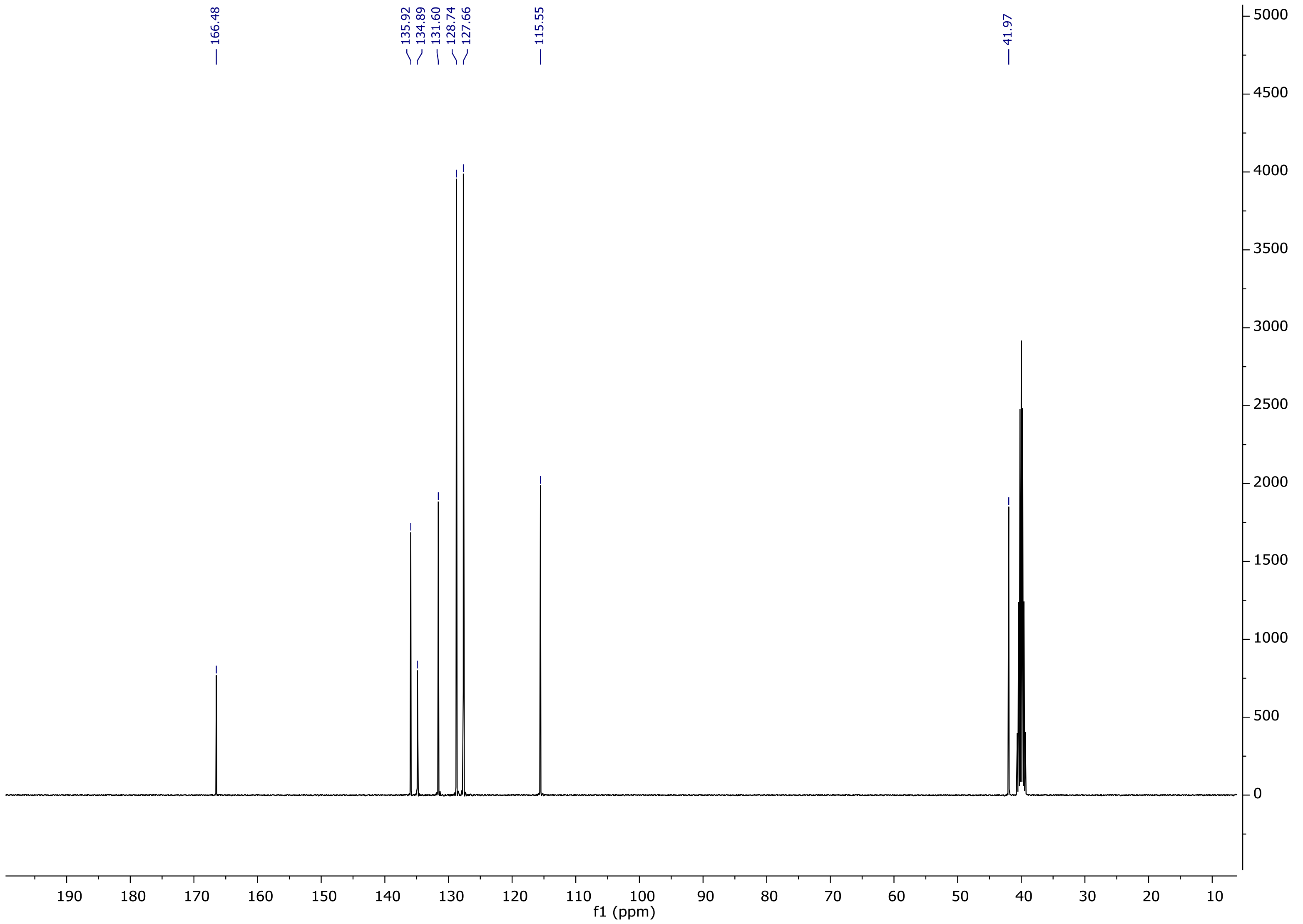
166.48
135.92
134.89
131.60
128.74
127.66
115.55
41.97
5000
4500
4000
3500
3000
2500
2000
1500
1000
500
0
190
180
170
160
150
140
130
120
110
100
90
80
70
60
50
40
30
20
10
f1 (ppm)

Ac-L-K^{Ac}AY-NH$_2$ (**5a**)

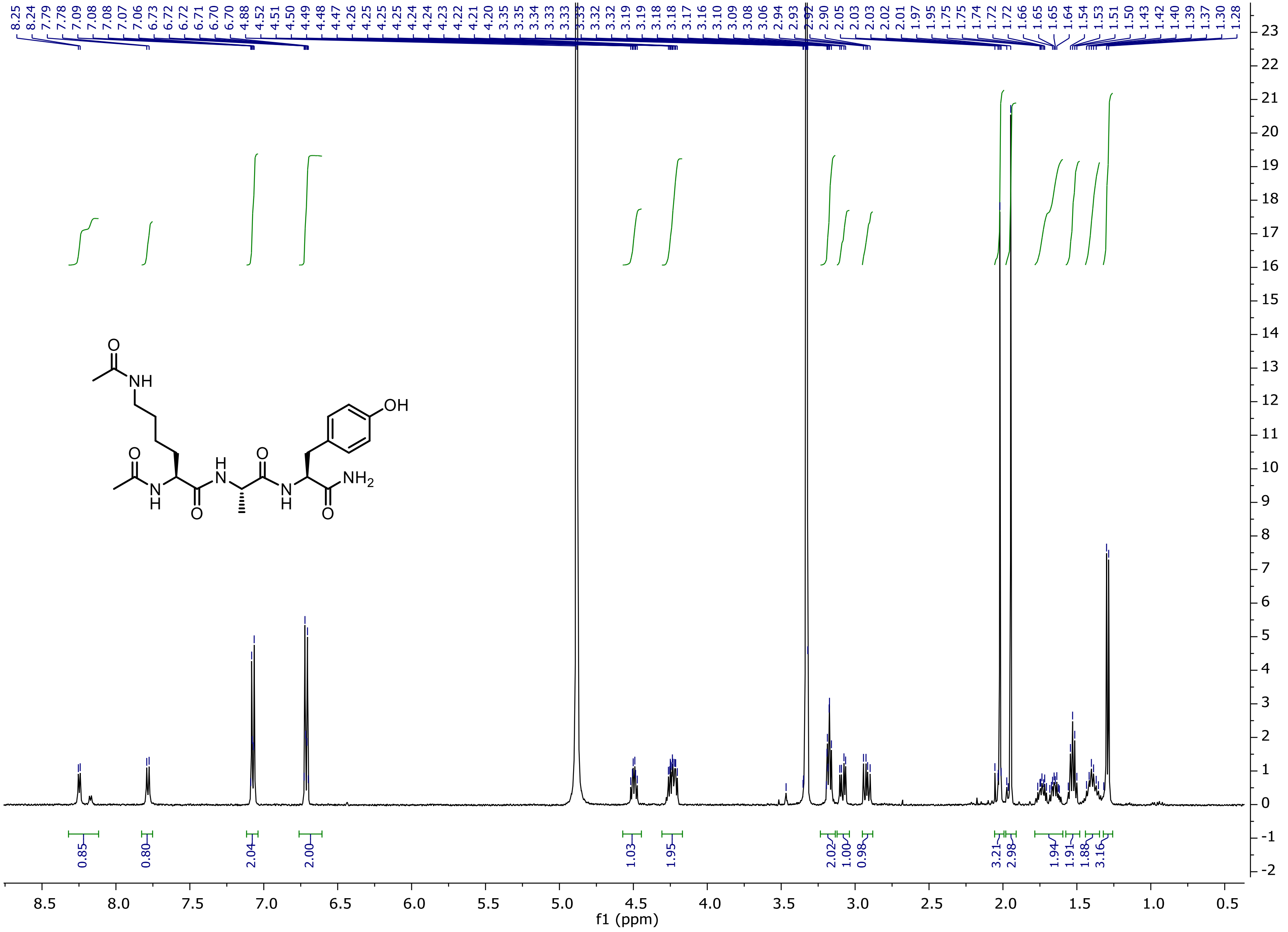

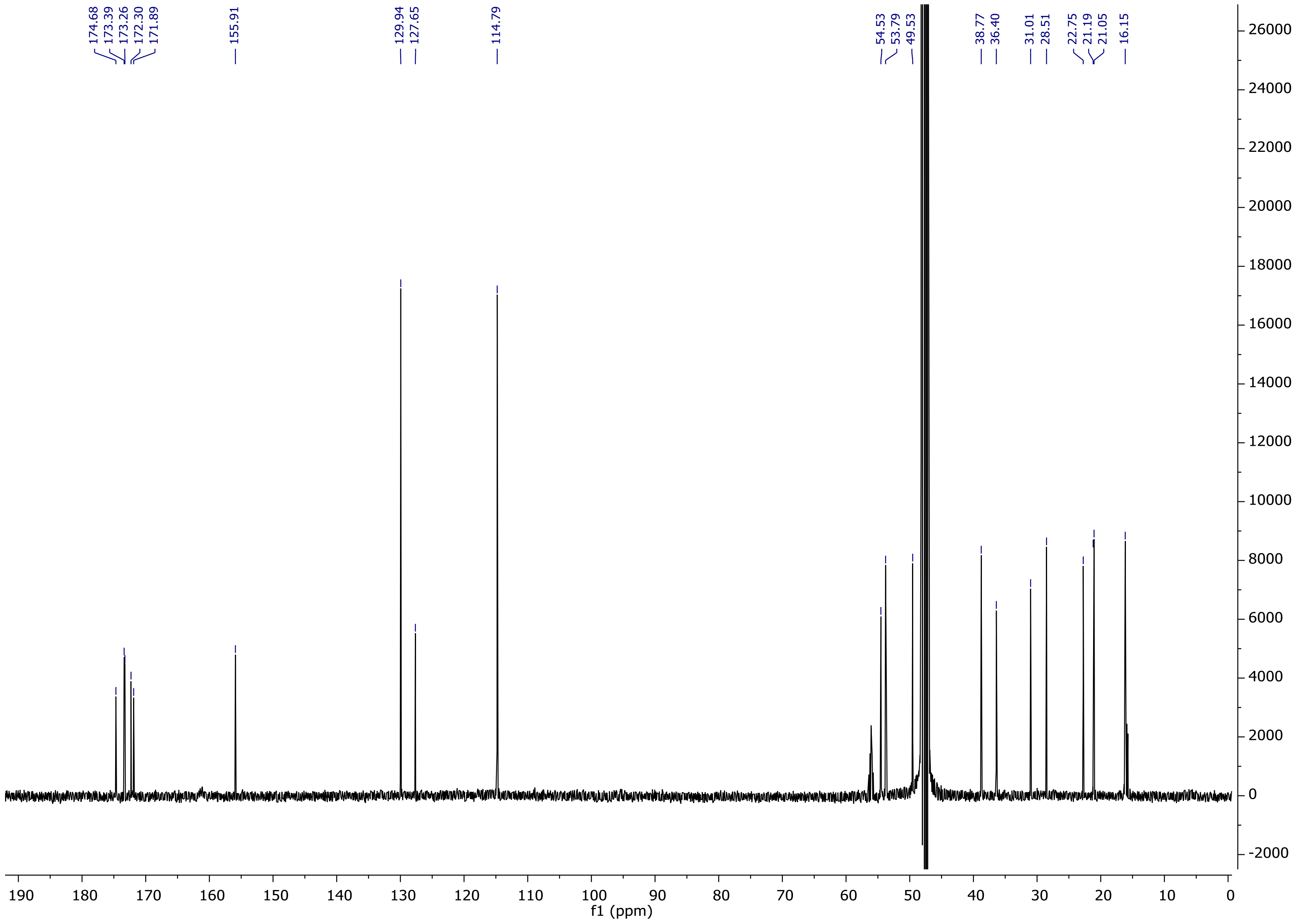

174.68
173.39
173.26
172.30
171.89
155.91
129.94
127.65
114.79
54.53
53.79
49.53
38.77
36.40
31.01
28.51
22.75
21.19
21.05
16.15
f1 (ppm)

Ac-D-K^{Ac}AY-NH$_2$ (**5b**)

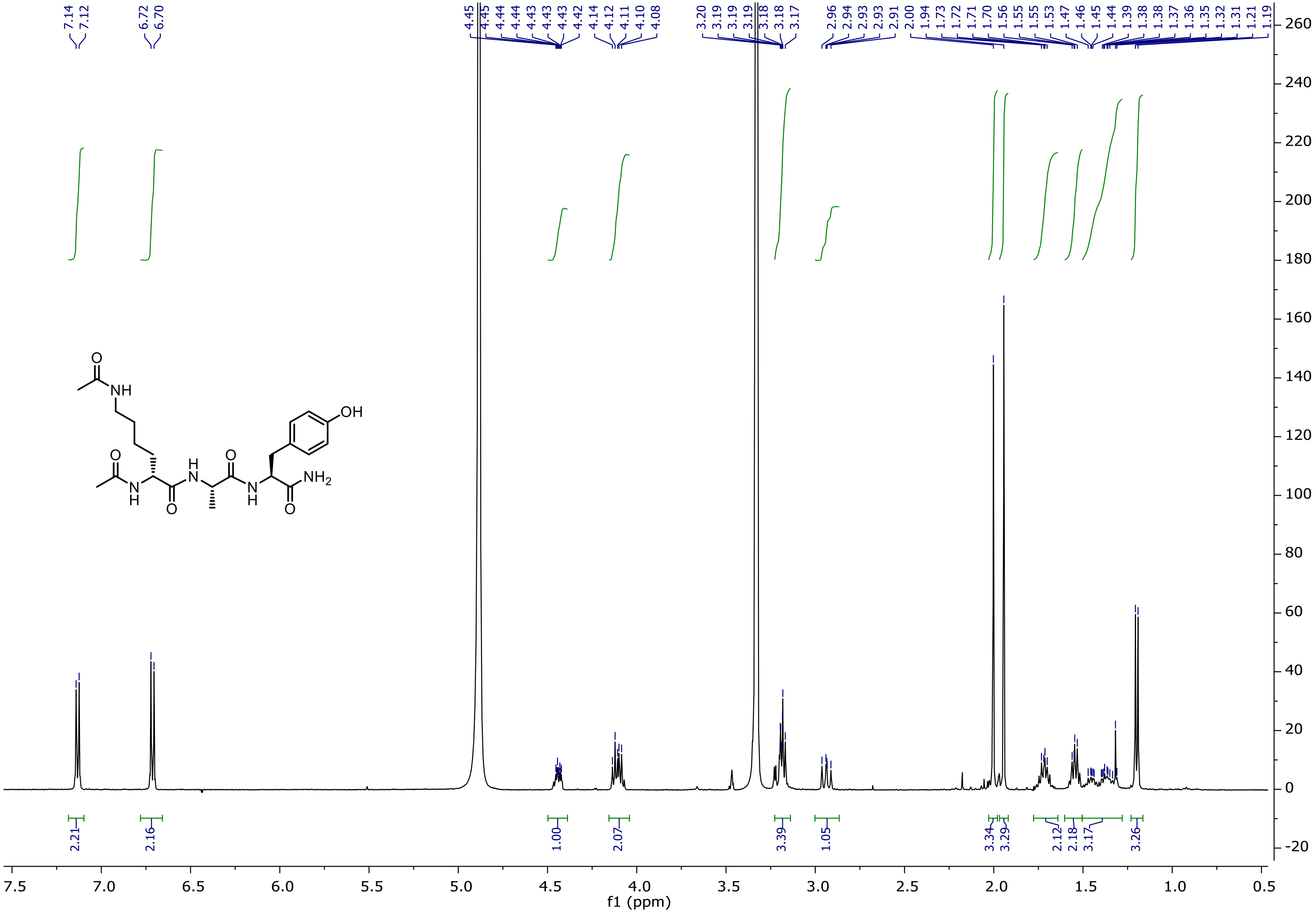

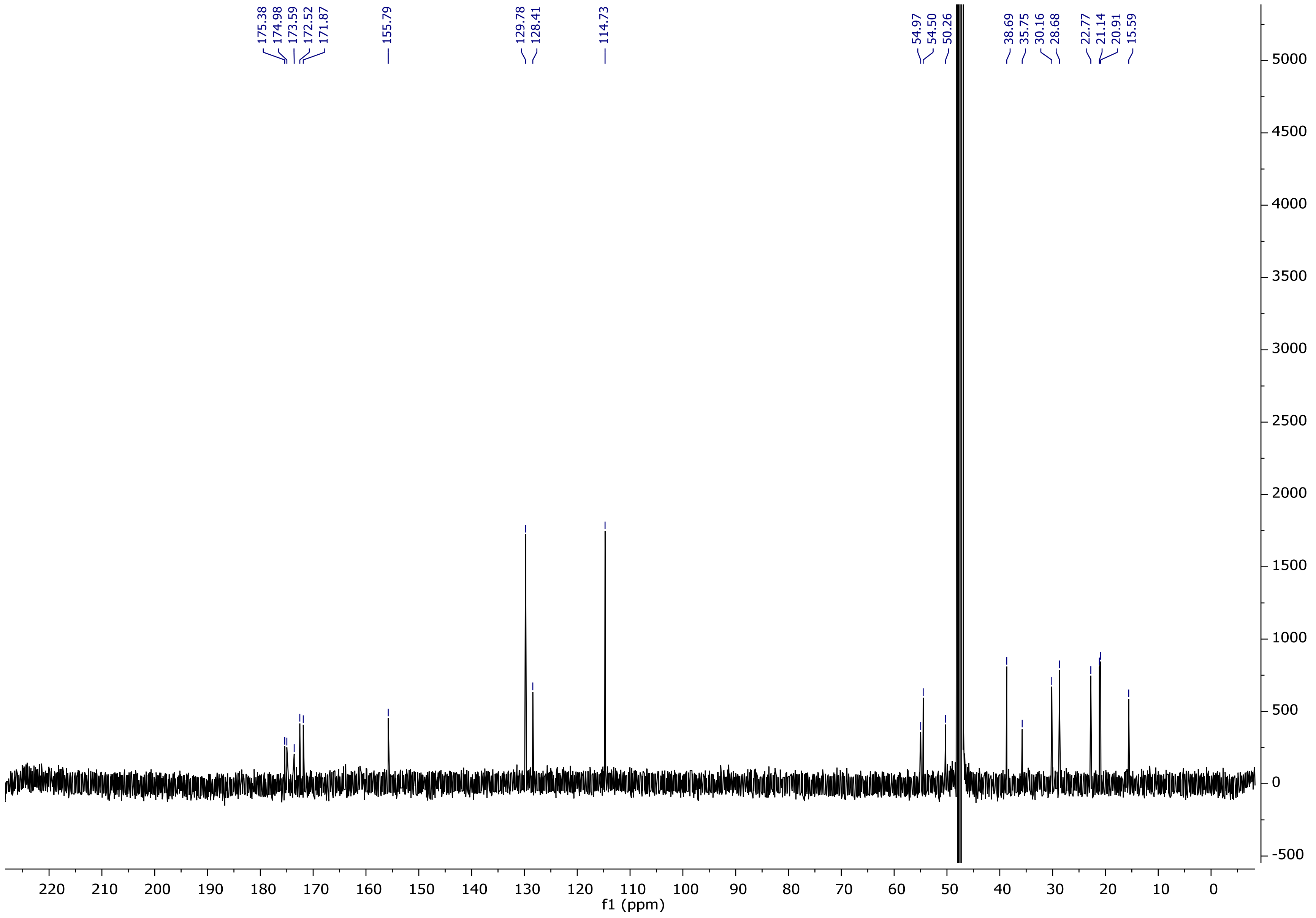
175.38
174.98
173.59
172.52
171.87
155.79
129.78
128.41
114.73
54.97
54.50
50.26
38.69
35.75
30.16
28.68
22.77
21.14
20.91
15.59
f1 (ppm)
